# Trapping or tethering stones (TS): A multifunctional device in the Pastoral Neolithic of the Sahara

**DOI:** 10.1371/journal.pone.0191765

**Published:** 2018-01-25

**Authors:** Marina Gallinaro, Savino di Lernia

**Affiliations:** 1 Dipartimento di Scienze dell’Antichità, Sapienza Università di Roma, Rome, Italy; 2 GAES, University of Witwatersrand, Johannesburg, South Africa; Institucio Catalana de Recerca i Estudis Avancats, SPAIN

## Abstract

The *Pierres de Ben Barour*, also known as trapping or tethering stones (TS), are stone artefacts with notches or grooves usually interpreted as hunting devices on the basis of rock art engravings. Though their presence is a peculiar feature of desert landscapes from the Sahara to the Arabian Peninsula, we know little about their age, context and function. Here we present a new approach to the study of these artefacts based on a large dataset (837 items) recorded in the Messak plateau (SW Libya). A statistically-based geoarchaeological survey carried out between 2007 and 2011 in Libya, alongside landscape and intra-site analyses of specific archaeological features (such as rock art, settlement and ceremonial contexts), reveal that these artefacts were used for a prolonged period, probably from the early Holocene. This was followed by a multifunctional use of these devices, particularly during the Pastoral Neolithic phase (ca. 6400–3000 cal BC), with the highest concentrations being found near ceremonial contexts related to cattle burials.

## Introduction

Trapping/tethering stones (TS) are a feature commonly found scattered in desert landscapes, and particularly across the Sahara. Their principal characteristic is the presence of one or more opposing notches on the longer axis and/or of an encircling groove suited to blocking a rope, whereas they are highly variable in terms of size, weight and raw material. In some regions they have come to represent a sort of iconic element of the landscape, but their functional interpretation and archaeological significance remains an open question.

The first reports of these artefacts quote an oral Arab legend recorded by the end of the 19th century in Algeria and later shared throughout the central Sahara e.g. [1–5]. According to this legend, these artefacts were used by a mythical figure known as Ben Barour as tools to mark the principal caravan routes crossing the Saharan desert. The stones were tied to a rope and dragged by a camel. Since then, such stones have frequently been recorded either as scattered artefacts or as representations in rock art scenes in different areas of the Sahara, from Algeria to Egypt. There are also sporadic reports of their presence in the Sahel [6], the Arabian Peninsula and the Near East e.g. [7–9].

In 1991, Pachur published a broad overview summarizing all the data known from the western to eastern Sahara, listing and debating the multiple functions of TS, and emphasising their role as palaeoenvironmental indicators of a greener Sahara. Traditionally, TS have been interpreted as hunting devices, mainly on the basis of a large rock art repertoire depicting these artefacts, mostly in association with wild game. Several authors have also proposed that TS were used to tame wild animals e.g. [10–16]. These interpretations are supported by a few ethnographic sources reported from different African contexts e.g. [17,18], including the Tuareg people inhabiting the central Saharan massifs e.g. [19]. However, since the earliest studies, their use for tethering domestic animals has also been considered. Pachur [20,21] himself, based on the archaeological data collected during the work he undertook with Gabriel in the Eastern Sahara [22], suggested that TS might have been used as a *‘fetter of grazing animals*, *especially cattle’* [21], a practice that still exists today among herders. Tying a TS to one leg allowed the animal to move but prevented it from straying.

A further use of TS as building materials was also suggested, for stone structures or as counterweights for tents, though this is commonly considered a secondary re-use see e.g. [21,23–25].

In recent years only a few scholars have paid any significant attention to TS, either in environmental or archaeological interpretations [25–29]. This is due mainly to the difficulty of dating the TS and of correlating them to any true archaeological context; these artefacts mainly occur on the surface and are often isolated, and are rarely found in contexts that can be accurately dated. As such, they have traditionally been ascribed to the long period spanning from at least 7500 BP until recent times [21].

**Fig 1:**
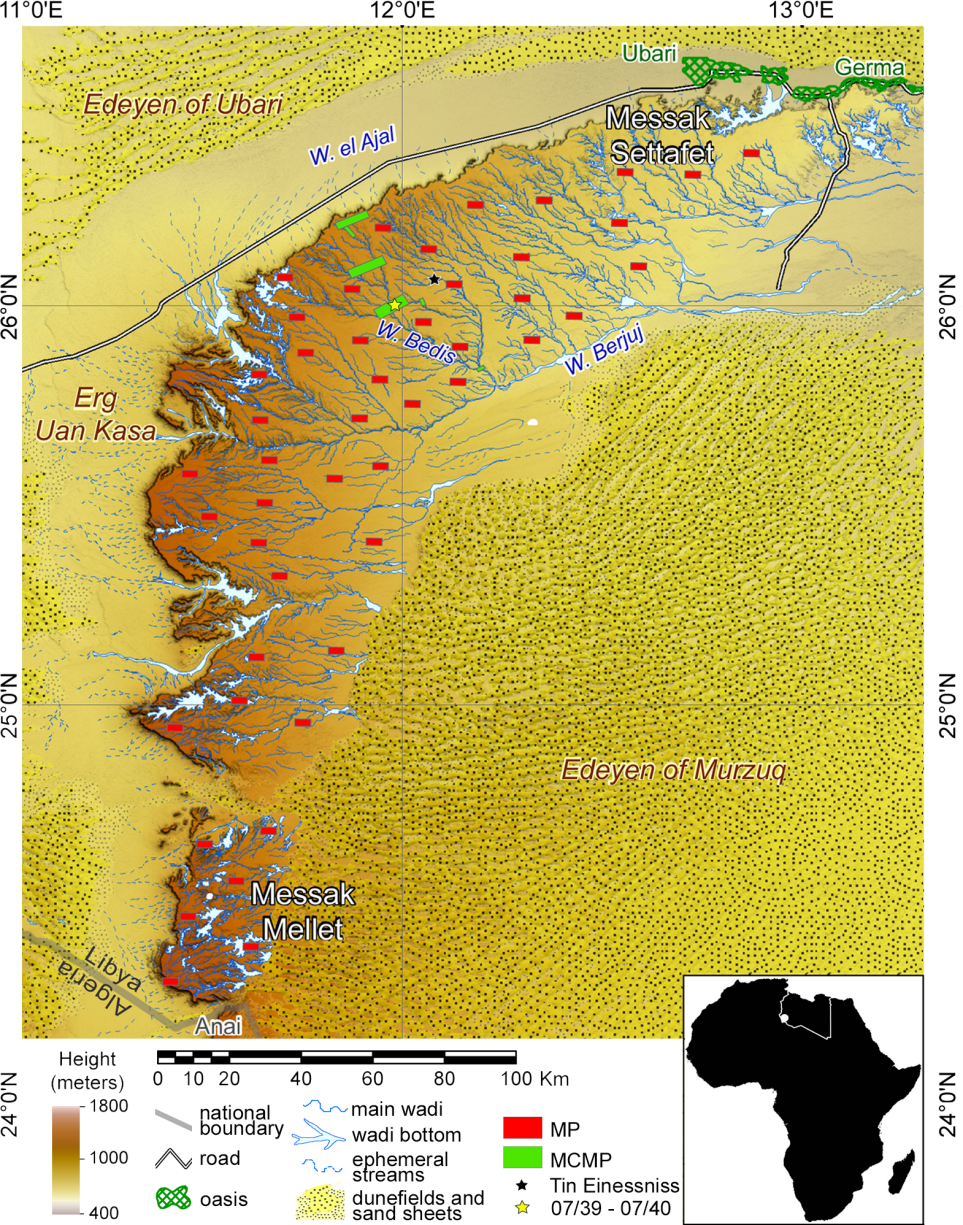
Overall map of the Messak plateau with the areas surveyed. (Key: MP, Messak Project; MCMP, Messak Ceremonial Monuments Project).

In this paper, we propose the first in-depth analysis of a large dataset of TS systematically recorded in a single region—the Messak plateau in southern Libya (Fig 1)—with the aim of investigating their potential uses in a well-defined archaeological and environmental framework. The descriptive and quantitative analysis of the principal characteristics of the TS, and of their relations with the region’s geormorphological and archaeological features, allowed us to demonstrate the multifunctional use of TS–rather than the exclusive use as hunting devices commonly proposed–, particularly during the Pastoral Neolithic phase (ca. 6400–3000 cal BC).

## The study area

### Environment and climate

The study area is the Messak Plateau in southwestern Libya (Fig 1). The Messak is a cuesta-type relief running N-S that becomes ENE-WSW oriented in its northern part [30], covering over 15,000 km^2^ and reaching a maximum altitude of 1200 m asl. A broad corridor divides the massif into two parts–named the Messak Settafet and Messak Mellet respectively–and a dry river network of dendritic wadis now cuts deep into its surface. It is therefore characterised by the presence of different physiographic units, consisting mainly of residual surfaces (mostly desert pavement), occasional outcrops of the sandstone bedrock, solutional depressions (also known as endorheic depressions), slope deposits, wadi valleys and a complex stepped escarpment on the northern and western sides [31]. Two major wadis mark the north-eastern (Wadi al Ajal) and south-eastern (Wadi Berjuj) edges, with different dune fields surrounding the plateau.

The massif now lies in a hyper-arid climatic region, where mean annual rainfall is between 0 and 10 mm [28], and the mean annual temperature ranges from 22° to 25°C. Evidence of wetter conditions have been identified for both the Middle and Late Pleistocene interglacials [30,32–36], and for the Early and Middle Holocene [37,38].

The flora includes the species described for the central Sahara, with Acacias (*Faidherbia albida*, *Acacia tortilis* and *A*. *nilotica*), shrubs of *Cornulaca monacantha*, *Pulicaria crispa*, *Panicum turgidum* and *Spipagrostis pungens* [25]. Desert savannah and Saharo-montane vegetation, typical of the Saharan Transitional zone [39], is prevalent in the wadis.

### Archaeological background

To contextualize these materials and narrow down the chronological interval to which they belong as much as possible, we should first summarize the main cultural evidence known for the area. There is a wealth of archaeological traces, widespread throughout the massif, including a heterogeneous set of remains spanning from the Early Pleistocene to historic times e.g. [40,41], whereas stratified archaeological deposits are almost absent due to the severe erosion typical of hyper-arid climates and the small number of caves and rock-shelters. The principal archaeological remains can be grouped into three general categories:

i. an almost uninterrupted palimpsest of lithic artefacts lying on the deflated surface, mainly reflecting different Pleistocene occupations, probably dating from the Early Stone Age (ESA) onwards with a preponderance of Middle Stone Age (MSA) artefacts [27,36,41–46];

ii. thousands of stone structures, ranging from funerary and ceremonial monuments to ephemeral and scarcely recognizable dwellings, e.g. [14,25,40,47–51]. These are the principal traces of the repeated Holocene occupations of the region from the Pastoral Neolithic to the Garamantian and post-Garamantian phases (radiocarbon dated from at least the beginning of the 6^th^ mill. cal BC to the 4^th^ century AD–[25] to the historical period (ca. 7th– 19th centuries AD, [27,50,52]) until the present Tuareg occupation [41]);

iii. a widespread rock art archive with thousands of isolated subjects, scenes or palimpsests -almost exclusively engraved- located on the steep banks of the complex network of wadis and on the exposed bedrock on the surface of the plateau e.g. [12,14,16,17,53–55]. There is heated debate over the chronologies and interpretations of rock art, but scholars generally agree that the Pastoral Neolithic phase, and in particular the Middle Pastoral (ca. 5200–3800 cal BC), was the most intense period of rock art production, given the extraordinary frequency of cattle depictions and the presence of specific stylistic traits [56] (with references).

The main occupation dynamics of the area, as reconstructed from the analysis of the archaeological features, are in keeping with the regional framework as defined in the nearby Acacus massif. However, in this context it is important to stress the archaeological invisibility of Early Holocene hunters and gatherers, a significant presence in the adjacent dune fields of the Edeyen of Murzuq [57–59]; this may reflect a very light, task-specific and/or ephemeral occupation.

## Materials and methods

All necessary permits were obtained for the study described, which complied with all the applicable regulations. The institution that granted the field permission is the Department of Archaeology, Tripoli (Libya). The materials discussed were recorded during two major projects carried out in the Messak massif between 2007 and 2011 (Fig 1). About 450 km^2^ were surveyed with two different aims and recording strategies that will be explained below; in both cases they included a combination of extensive survey by car, intensive survey on foot, detailed mapping and selective excavations.

The first project—the *Messak Ceremonial Monuments Project* (MCMP [25])—undertaken between 2007 and 2010, aimed to investigate ceremonial sites linked to the ritual deposition of cattle in monumental stone structures, attested in the area from at least the end of the 6^th^ mill. cal BC [25], with references]. Six sampling areas of different sizes–chosen thanks to a detailed desktop study of satellite imagery–lay along a N–S transect, located in the central area of the Messak Settafet.

The second project- *Messak Project*. *Cultural and Natural Preservation and Sustainable Tourism*, undertaken between 2010 and 2011 (MP, [41,60,61]), was aimed at increasing our overall knowledge of the environmental and cultural heritage of the plateau. In an area severely affected by oil exploitation e.g. [62–64], the MP aimed to assess the damage and future potential risks to the region, with a view to proposing specific management plans for its restoration, conservation and sustainable development. The plateau was systematically surveyed, with 46 sample transects (4x2 km each) on average 10 km apart, placed to form a regular grid of surveyed areas, including unexplored or little known areas and intersecting different geomorphological features. This dataset will be used here as the baseline for the first stage of analysis, as it can be assumed to constitute the statistically most reliable territorial dataset, covering the whole Messak region.

Other data from a previous rescue survey carried out in the central part of the massif, in connection with oil exploitation activities (*Messak Settafet Road Survey* -MSRS, [27]), will not be discussed in detail here, as the survey methods were too different for direct comparison. Only one specific area with a significant concentration of TS (Tin Einessniss) will be briefly considered in the discussion.

### The dataset

A total of 837 TS were identified and recorded throughout the Messak during the MCMP and MP projects (Fig 1). This constitutes the largest dataset of TS ever recorded in a single region. The main characteristics of TS in terms of size, weight, shape and find spot will be analysed and discussed here, both from a landscape perspective and on a local scale. As concerns the raw material, all the TS were made by exploiting the exposed sandstone outcrops and this allows us to calculate their weight based on size and the specific gravity of sandstone.

For each TS the following information was recorded and classified (Table 1, S1 Appendix): i. geographical coordinates and topography of its location, including information about the geographical and geomorphological contexts recorded in the field and on the geomorphological map of the plateau [31]; ii. TS features, including size, calculated weight, shape type and surface varnish; iii. intra-site features, including information about the archaeological context of the TS.

**Table 1:** Structure of the TS database.

**i. Position and Topography**							** **	
ID_TS	site name	transect no.	project	lat.	long.	topography	geomorphology	
**ii. TS features**								
length (cm)	width (cm)	thickness (cm)	no. of notches	groove	weight*	weight class	rock varnish	shape
**iii. intra-site info**								
context	position							

* the weight was calculated based on size and the specific gravity of sandstone, ca. 2.3 kg/dm^3^.

## Results

The analysis of the data confirms the high variability of the TS in terms of size, weight and shape. However, we can isolate and discuss some significant trends in their principal features. The weights, calculated using the recorded dimensions, vary widely from a few kilos (3.3 kg) to hundreds of kilos (294 kg): they were grouped into 6 classes, defined on the basis of the distribution of frequencies. Two categories accounted for a majority of TS (10–26 kg = 34%; 26–50 kg = 30%), with low percentages of the lightest (1–10 kg = 9%) and heaviest (85–146 kg = 7%; 146–294 kg = ca. 2%) classes (Table 2). In the discussion that follows, these weight classes are reduced to three, given the limited numbers in the lightest and heaviest classes, in order to match their statistical significance. We will therefore refer simply to *light* (≤1–26 kg), *medium* (26–85 kg) and *heavy* (≥ 85 kg) TS (Table 2).

**Table 2:** Frequencies of weight classes*.

Weight classes	No. of TS	%	Simplified weight classes	No. of TS	%
**1 = 1–10**	66	9.1%	Light	315	43.2%
**2 = 10–26**	249	34.2%			
**3 = 26–50**	220	30.2%	Medium	351	48.1%
**4 = 50–85**	131	18.0%			
**5 = 85–146**	50	6.9%	Heavy	63	8.6%
**6 = 146–295**	13	1.8%			
**Total**	729	100.0%		729	100.0%

* TS presenting incomplete recorded values are not included in the table.

**Fig 2:**
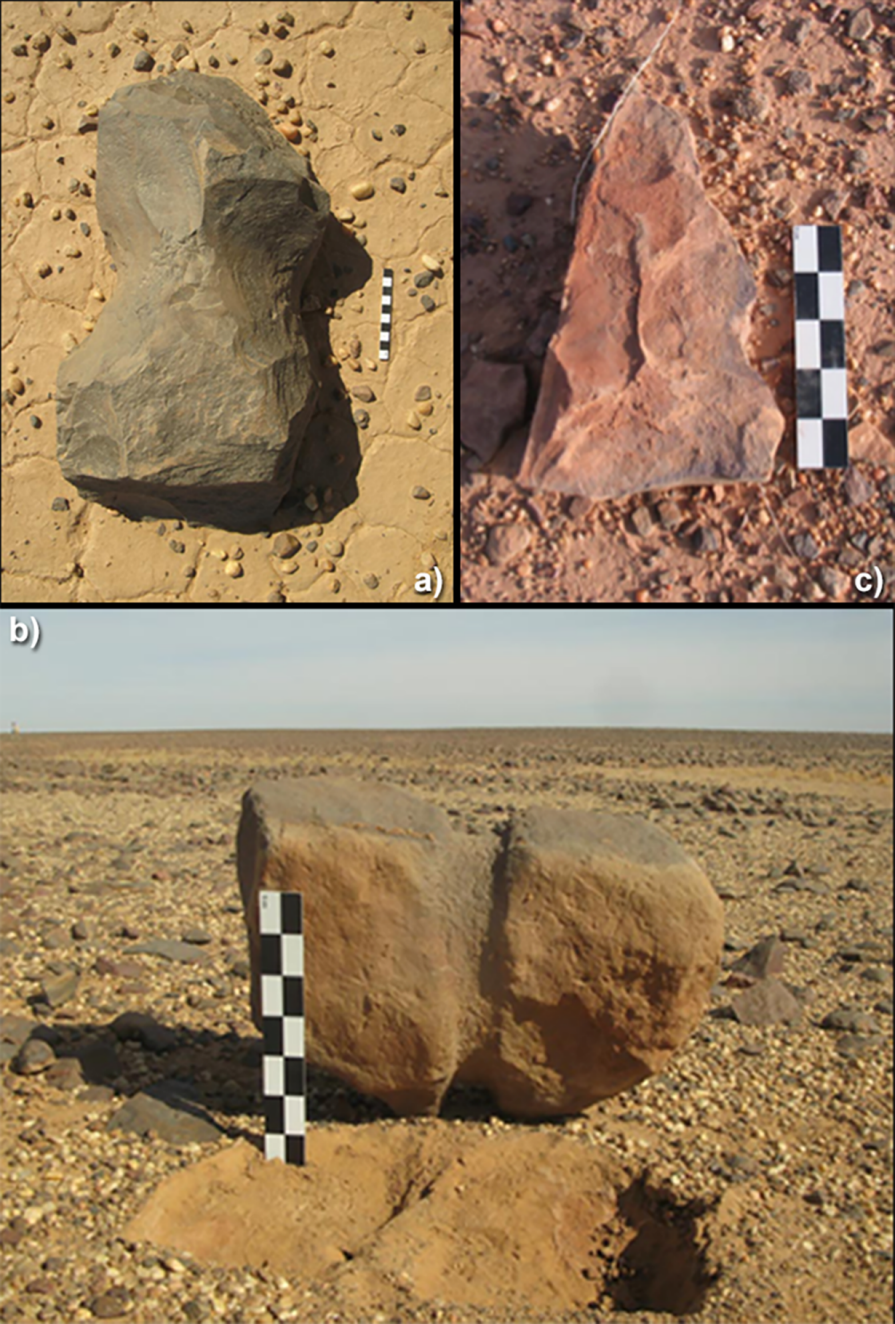
Simplified typology of TS. a) *shaped*; b) *heavy grooved*; c) *opportunistic*.

TS are solids with a remarkably high degree of morphological diversity that can potentially be approximated to known geometrical shapes (cylinders, parallelepipeds, etc.). However, all detailed formal typologies turned out to be unable to concretely represent this variability. Therefore, we adopted a morpho-technological approach emphasising the main feature of the TS: the notches and/or the circular groove. We thus isolated three classes (Fig 2). The first consists of *shaped* TS with two notches indicating a careful selection of the original stone and particular care in flaking to obtain a specific shape. This class is the most frequent, with almost 60% of the total. The second class (ca. 29%) was termed *opportunistic*: it consists of TS with one or two simple notches, which represent the only alteration to the original shape of the stones. A third category (ca. 12%), labelled *heavy grooved*, is represented by TS with a deep and carefully refined encircling groove; these TS often show signs of weathering and have more rounded shapes.

Another significant datum for the position and the determination of chronology is the rock varnish. This is a dark, Mn-rich weathering surface that coats rock surfaces exposed in arid lands. In the central Sahara, according to the geomorphological and archaeological data [56,65,66], this varnish started to form from the mid-Holocene, under progressively more arid conditions, approximately between the end of the 5^th^ and the second half of the 3^rd^ mill. cal BC. In the cases recorded, ca. 60% of TS present the dark varnish only on the exposed side, with the surface in contact with the soil being orange-brown; the remaining 40% has a dark varnish on both sides. It is worth noting that unvarnished notches or grooves on fully black varnished TS are lacking; these must therefore have been made before the end of the varnishing period. These results, though based on an indirect and rather approximate chronology, suggest that the final position of the 60% of TS with a black/red surface dates to the Early/Middle Holocene and that the production of these artefacts ended in the second half of the 3^rd^ mill cal BC, though older TS may have been reclaimed and re-used.

### Landscape perspective

**Fig 3:**
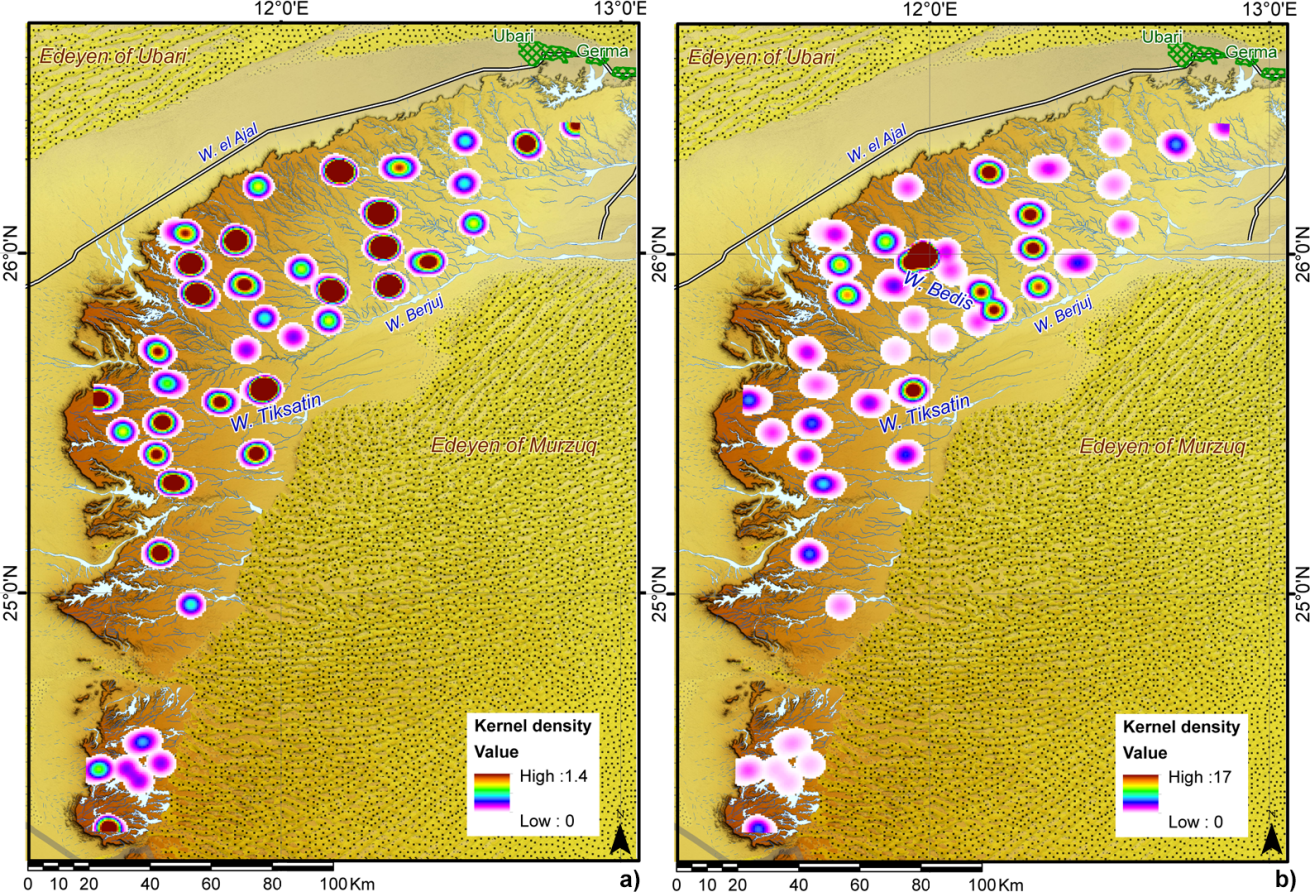
Kernel density analyses of TS. a) analysis on TS recorded during the Messak Project survey; b) analysis on the whole dataset.

As concerns the geographical location of TS, it is worth noting that none of these stones are located in the wadis; instead, all the recorded artefacts were found either on the surface of the hamada (86%) or to a lesser extent along the hamada edge (14%). However, their presence in different geomorphological contexts such as desert pavements (ca. 50% of the total TS), endorheic depressions (ca. 45%) and slopes (ca. 5%) suggests a differentiated use of the landscape (Table 3). The surface of the plateau is affected by strong erosion due to the hyper-arid climate. However, this process mainly results in deflation and the ensuing vertical erosion, with no substantial effect on the XY position of lithic artefacts. The current location of TS cannot be related to any post-depositional processes other than anthropic actions, as demonstrated by the geomorphological interpretation of the formation processes of the desert pavement of the plateau [31,35] and the development of rock varnish in the area [66]. Indeed, these frequencies take on a different significance if we compare the extension of each different geomorphological feature and match it exclusively with the data collected during the MP survey; the latter is the most reliable dataset given the systematic sampling strategy. Using the geomorphological map by Perego and colleagues [31], we can roughly estimate that endorheic depressions represent under 1% of the total surface of the plateau, whereas desert pavement constitutes ca. 67% and the slope ca. 21% of the area (the remaining 11% are wadis). Yet almost 80% of the TS recorded during the MP survey were found in endorheic depressions, almost double the percentage observed on the whole dataset; 15% occur on the desert pavement and only 4% on the slope. This association between TS and endorheic depressions seems extremely clear and holds true all over the massifs, as the landscape analysis confirms. The kernel density calculated on the MP survey dataset shows that TS were widespread throughout the massif, with a particularly high density in different portions of the central area of the plateau (Fig 3A), and another single major density area in the west, near Wadi Tiksatin.

**Table 3:** Cross tabulation of topography/geomorphology, by project.

Project				Geomorphology			Total
				desert pavement	endorheic depression	slope	
MCMP	Topography	hamada	no.	259	86	4	349
			%	55.5%	18.4%	0.9%	74.7%
		hamada edge	no.	99	0	19	118
			%	21.2%	0.0%	4.1%	25.3%
	Total		no.	358	86	23	467
			%	76.7%	18.4%	4.9%	100.0%
MP	Topography	hamada	no.	57	295	18	370
			%	15.4%	79.7%	4.9%	100.0%
	Total		no.	57	295	18	370
			%	15.4%	79.7%	4.9%	100.0%
Total	Topography	hamada	no.	316	381	22	719
			%	37.8%	45.5%	2.6%	85.9%
		hamada edge	no.	99	0	19	118
			%	11.8%	0.0%	2.3%	14.1%
	Total		no.	415	381	41	837
			%	49.6%	45.5%	4.9%	100.0%

To sum up, the overall distribution is mainly characterised by the high frequency of TS in endorheic depressions where TS occur in almost all the transects surveyed, either as isolated artefacts or in small groups of up to ten. Only in the smaller southern part of the massif, which is intersected by broad wadis, did we record a higher frequency of TS on the slope and an almost total absence in endorheic depressions. The distribution on the desert pavement is highly irregular, a fact which can be more accurately interpreted if we also consider the data collected by the MCMP Project.

The MCMP data provide distribution and density patterns that partially differ from those of the systematic MP survey. Indeed, this dataset shows an almost inverse ratio of frequency by geomorphology (19% in endorheic depressions *vs* 77% on desert pavements, while the slope value remains constant, i.e. around 5%—Table 3), and significant clustering in a few main spots that include endorheic depressions and above all a specific area of the desert pavement along the main meander of Wadi Bedis (Fig 3B). The latter is home to a cluster of at least two major ceremonial contexts (07/59-07/68 and 07/39-07/40) that have been connected to the Middle Pastoral ritual centred on the deposition of disarticulated animal remains in stone structures of the *corbeille*-type (i.e. ‘basket’-shaped: these are circular platforms with slabs set obliquely around their external perimeter often with an associated standing stone) and tumuli [24,25]. A more detailed Point Density Analysis run on the whole dataset, assuming a range area of 100 m, allowed us to compare the density value of TS/ha in the whole recorded area (Table 4). The highest densities were recorded in the ceremonial contexts 07/59-07/68 and 07/39-07/40, reaching peaks of 86 and 147 TS/ha respectively. No other area recorded anywhere in the Messak presents such a high density, except Tin Einessniss 1: interestingly, this is another ceremonial area rich in rock art, *corbeille*-structures and tumuli with remains of disarticulated cattle, investigated during the MSRS project, along the wadi of the same name and located ca 12 km NE of Wadi Bedis [25–27,56]. In the endorheic depressions, the highest density recorded is 41 TS/ha, about 1 km SE of the Wadi Bedis meander (Site 07/34); elsewhere, on the plateau as a whole, the density value ranges from 3 to 5, and up to 10 (Table 4). In other words, the density in ceremonial areas is up to three times the highest recorded value in endorheic depressions.

**Table 4:** Results of point density analyses.

Density value (TS/ha)	frequency			
	total	geomorphology		
		desert pavement	endorheic depression	slope
1	174	44	116	14
2	35	13	56	1
3	19	6	51	0
4	7	4	16	8
5	11	5	45	5
6	3	12	6	0
7	4	14	14	7
8	1	0	8	0
10	2	10	10	0
14	1	1	0	0
18	1	0	18	0
27	1	27	0	0
41	1	0	41	0
47	1	47	0	0
86	1	73	0	13
146	1	146	0	0

**Fig 4:**
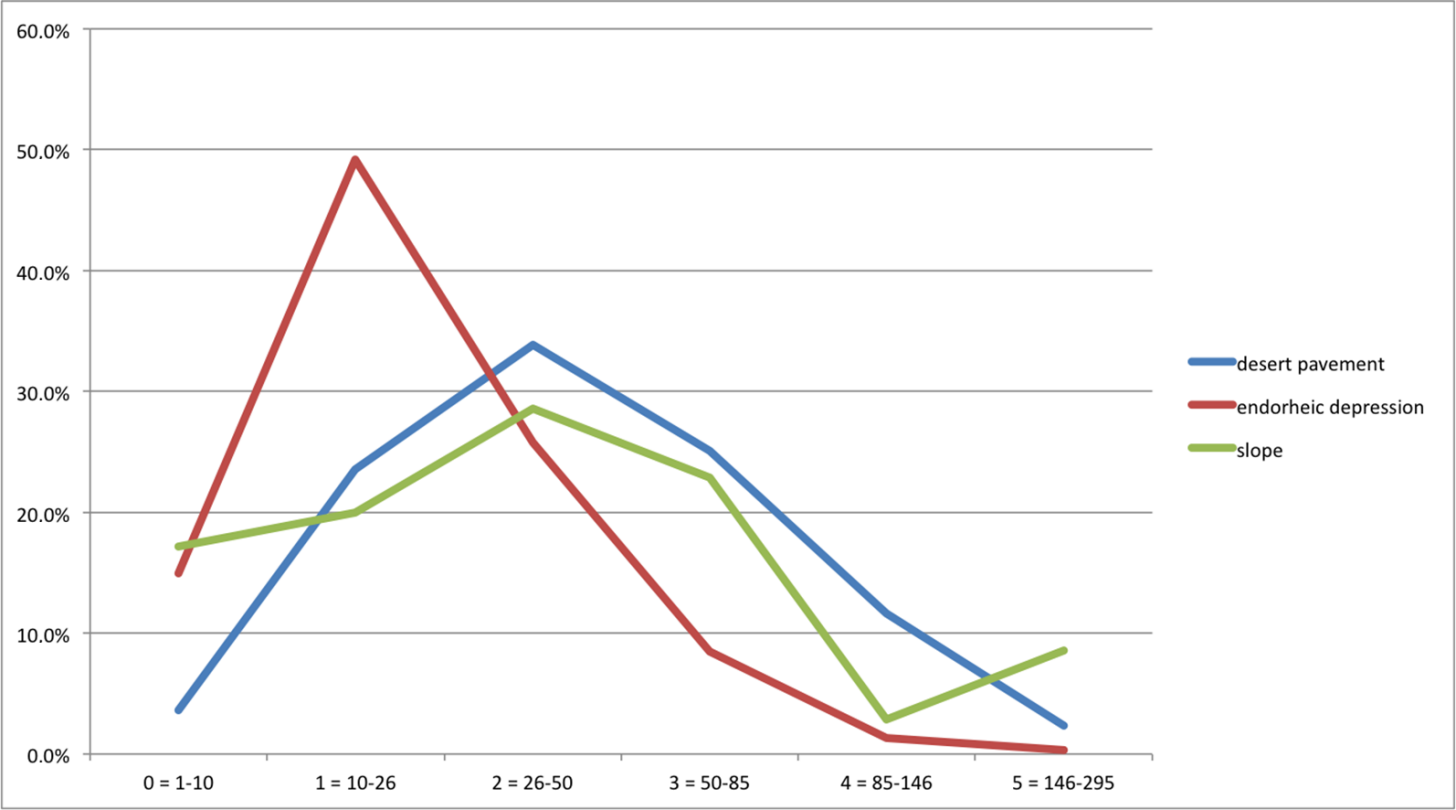
Graph showing the weight class frequency for the whole TS dataset.

The distributions obtained appear to correspond to a differentiated use of the landscape, closely connected to geomorphological features and therefore to the varied palaeo-ecological environments, likely implying that the TS had different functions. These diversified functions are also suggested by the different patterns resulting from the analysis of weight frequencies as compared to the distribution of TS shapes by geomorphology. The endorheic depressions contain a high proportion of light stones (about 64% of the TS are lighter than 26 kg—Fig 4) and more defined shapes are recorded (*heavy grooved* and *shaped* TS represent over 2/3 of the sample—Table 5). By contrast, on the desert pavement and on the slope, the medium and heavy weight classes prevail (73% and 64% of TS respectively weigh over 26 kg), and the shapes are more variable, with a high proportion of *opportunistic* TS (Table 5). Chi-square tests (χ^2^) confirm that the correlations between the features of TS (weight and shape) and their geomorphological context are statistically significant, refuting the null hypotheses that TS are distributed by weight (χ^2^: 100.38, degree of freedom: 4, ρ: 0) and shape (χ^2^: 49.571, degree of freedom: 4, ρ: 0) according to the (random) proportional extension of each geomorphological unit.

**Table 5:** Cross tabulation of geomorphology/shape, by project*.

Project				shape			Total
				heavy grooved	opportunistic	shaped	
MCMP	Geomorphology	desert pavement	no.	6	147	191	344
			%	1.4%	33.8%	43.9%	79.1%
		endorheic depression	no.	3	23	43	69
			%	0.7%	5.3%	9.9%	15.9%
		slope	no.	1	13	8	22
			%	0.2%	3.0%	1.8%	5.1%
	Total		no.	10	183	242	435
			%	2.3%	42.1%	55.6%	100.0%
MP	Geomorphology	desert pavement	no.	23	5	29	57
			%	7.4%	1.6%	9.4%	18.4%
		endorheic depression	no.	49	30	162	241
			%	15.8%	9.7%	52.3%	77.7%
		slope	no.	6	1	5	12
			%	1.9%	0.3%	1.6%	3.9%
	Total		no.	78	36	196	310
			%	25.2%	11.6%	63.2%	100.0%
Total	Geomorphology	desert pavement	no.	29	152	220	401
			%	3.9%	20.4%	29.5%	53.8%
		endorheic depression	no.	52	53	205	310
			%	7.0%	7.1%	27.5%	41.6%
		slope	no.	7	14	13	34
			%	0.9%	1.9%	1.7%	4.6%
	Total		no.	88	219	438	745
			%	11.8%	29.4%	58.8%	100.0%

* TS presenting incomplete recorded values are not included in the table.

### Archaeological contexts and intra-site data

**Fig 5:**
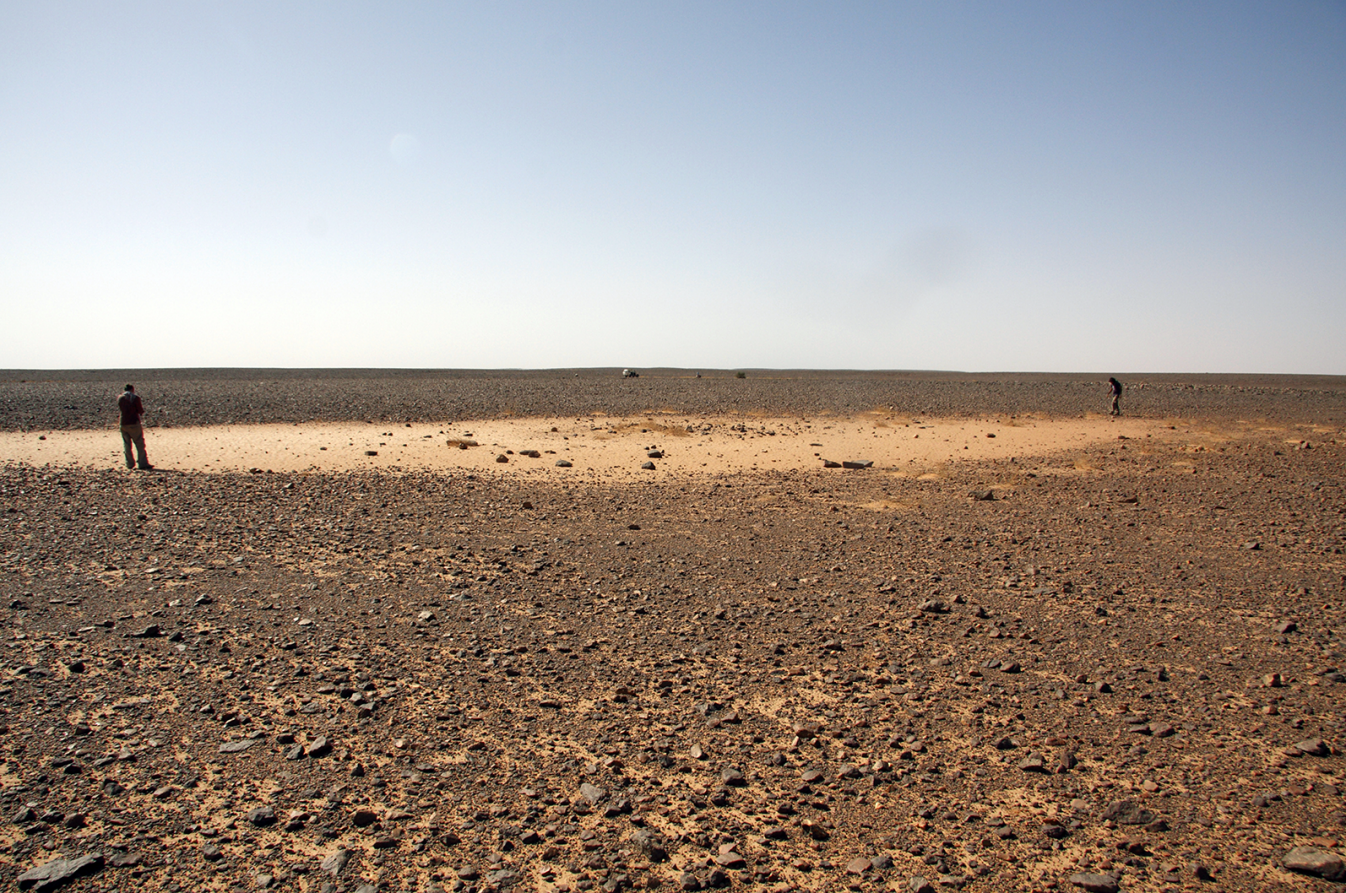
Example of a Pastoral Neolithic campsite in an endorheic depression. Site 10/699 located in the Messak Mellet.

Almost 1/5 of the TS (146) occur as isolated finds in each of the geomorphological features; there is no evidence on the basis of which to date these and they presumably reflect a widespread use throughout the landscape. The highest percentage of these isolated TS occurs in the endorheic depressions, and they are mainly of the *shaped* and *heavy grooved* types (Table 6). These depressions are geomorphological features particularly suited to retaining moisture and are characterized, even today, by the presence of a denser vegetation cover, i.e., discontinuous grass cover. Although it is difficult to say for certain, they might reflect task-oriented activities, likely including hunting. Though re-cycling and/or post-depositional effects may have significantly affected the distribution of TS, the principal concentrations occur in well-defined archaeological contexts that chronologically point to the Pastoral phases. In the endorheic depressions, TS occur in probable association with the remains of temporary campsites (Fig 5). These are characterized by the presence of a peculiar kind of deflated fireplaces, signalled by pebbles or fragments of fire-cracked rocks forming small mounds known as *hearth-mounds*, after [67] or *Steinplätze*, after [68,69], and sometimes of other deflated stone structures, as well as grinding equipment, flaked lithic artefacts and a very few fragments of pottery. In these contexts, TS are almost exclusively scattered on the surface and are only very occasionally used (or re-used) as building materials for stone structures. The chronology of these contexts is particularly difficult to ascertain due to the high degree of erosion of the fireplaces. As already mentioned, these areas were repeatedly occupied from the ESA to modern times, but none of the recorded TS can at this stage be positively correlated with remains of phases other than those of the Pastoral period. The presence of the black varnish exclusively on the exposed side of the TS, as on old varnished stones, and their coexistence with a specific type of fireplace structures (*Steinplätze*) traditionally associated with temporary Pastoral Neolithic campsites e.g. [22,70], both cautiously support this chronological attribution. The Pastoral Neolithic date and the intra-site location suggest that they were mainly used as tethering tools for domestic grazing livestock.

**Table 6:** Cross tabulation of geomorphology/shape of isolated TS.

			shape			Total
			heavy grooved	opportunistic	shaped	
**Geomorphology**	**desert pavement**	no.	19	4	11	34
		%	13.0%	2.7%	7.5%	23.3%
	**endorheic depression**	no.	23	13	69	105
		%	15.8%	8.9%	47.3%	71.9%
	**slope**	no.	3	0	4	7
		%	2.1%	0.0%	2.7%	4.8%
**Total**		no.	45	17	84	146
		%	30.8%	11.6%	57.5%	100.0%

**Fig 6:**
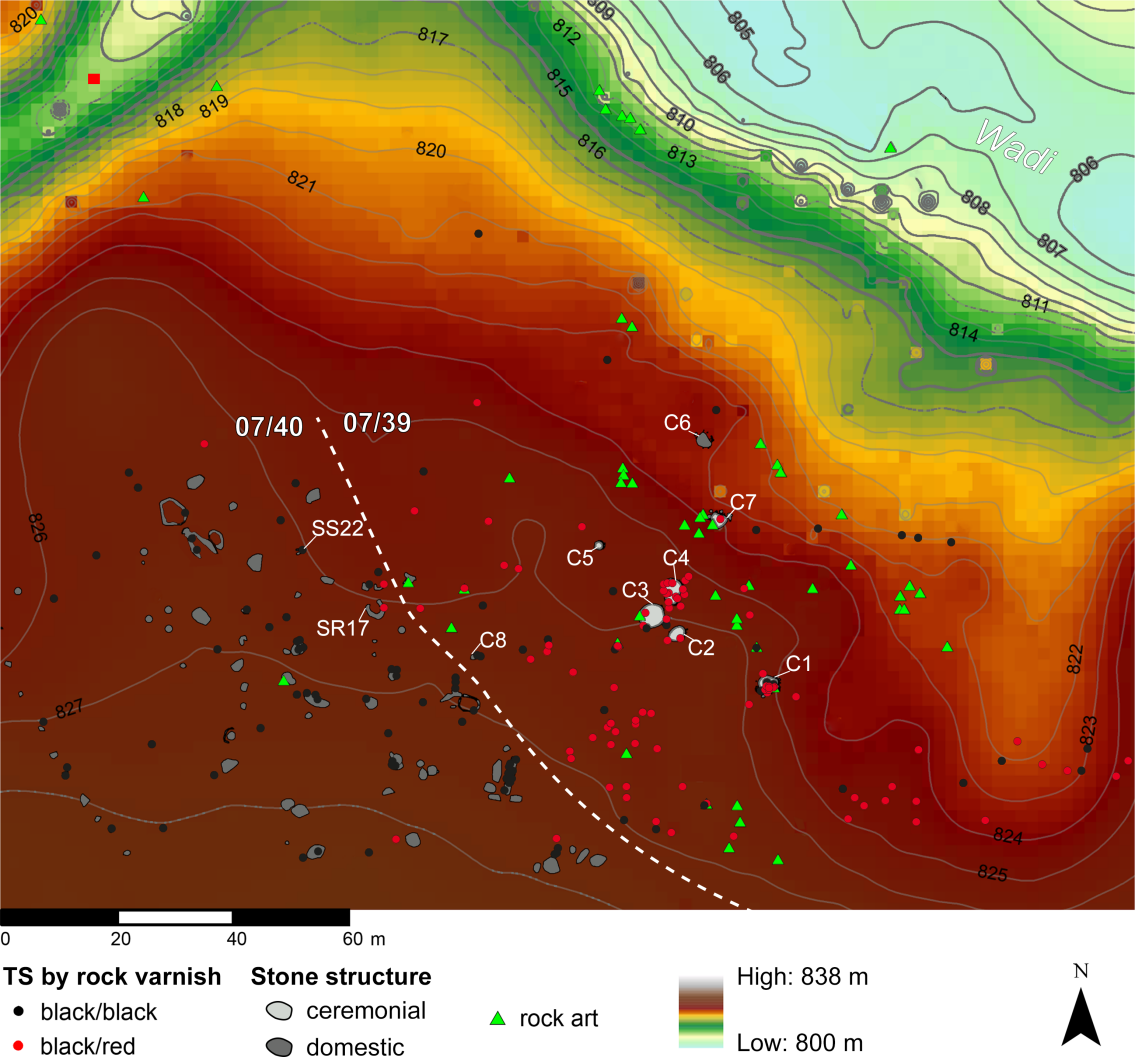
Overall map of sites 07/39 and 07/40. The white dashed line indicates the edge of the site.

As noted above, the highest densities of TS on desert pavement occur in ceremonial contexts, inside which they may be located in different positions. A representative instance is the complex of sites 07/39 and 07/40 –also those with the highest density of TS. These are two adjacent sites, located on the right-hand bank of the main meander of Wadi Bedis. This area is particularly rich in rock art, funerary and ceremonial structures, as well as other stone features that can be ascribed to campsites (Fig 6). Indeed, Site 07/39 is one of the main Middle Pastoral ceremonial sites, with 8 stone structures (seven of which are of the *corbeille*-type). Site 07/40 comprises at least 65 stone structures of different types that have been interpreted as the remains of temporary campsites, repeatedly occupied by the human groups that re-used the area over several millennia, as attested by the two latest radiocarbon dates obtained from two of these structures, pointing to the Final Pastoral (07/40 SR17: 1306–1126 cal BC) and to the Garamantian period (07/40 SS22: 134–325 AD; [25]) (Table 7) This occupation has been interpreted as a form of revitalisation of the past pastoral and cultic landscape, whose importance is also attested by the 45 areas of rock art engraved on exposed outcrops or stones in between and on the structures of both sites [25,56].

**Table 7:** Table of radiocarbon datings from sites 07/39 and 07/40).

Site	Structure	Lab. Code	Material	Age uncal BP*	Cal BC/AD (95.4% conf.)	Cal BP (95.4% conf.)	d13C,%
07/39	07/39 C6	UGAMS 3760	charred bone	5660±30	4554–4375	6502–6323	10.19
07/39	07/39 C3	UGAMS-3758	charcoal	5520±30	4449–4331	6399–6280	-23.46
07/39	07/39 C4	UGAMS-2839	charred bone	5430±40	4354–4177	6304–6125	-16.74
07/39	07/39 C8**	UGAMS 3761	charcoal	5400±30	4338–4172	6288–6034	25.86
07/39	07/39 C5	UGAMS 3759	charred bone	5200±30	4047–3961	5997–5909	13.92
07/39	07/39 C1	UGAMS-3757	charred bone	5190±30	4044–3960	5993–5908	-13.79
07/40	07/40 SR17	UGAMS 5854	charcoal	2980±25	1277–1121	3255–3075	25.6
07/40	07/40 SS22	UGAMS 5857	charcoal	1790±25	136–326 AD	1816–1625	25.0

* The term 'BP' refers to uncalibrated years before present, according to Libby's half-life. Calibration using OxCal online version 4.3 using IntCal13 calibration curve

** published in [25] as 07/40-38

At Site 07/39, the highest percentage of TS was located on the surface (65%; Table 8); highly clustered distributions occur along a semi-circular line surrounding the central group of *corbeilles*. The remainder of the sample from Site 07/39 (35%) was recorded directly inside the *corbeilles*, with a particularly high proportion (up to 80% of the subtotal) in three out of seven: 15 TS in C4, 14 TS in C1 and 7 TS in C3. *Shaped* TS prevail both as building materials and outside the structures.

**Table 8:** Cross tabulation of position/shape, by site (07/39 and 07/40).

site				shape			Total
				heavy grooved	opportunistic	shaped	
**07/39**	**position**	**building material**	**no.**	0	19	26	45
			**%**	0.0%	14.6%	20.0%	34.6%
		**surface**	**no.**	3	33	49	85
			**%**	2.3%	25.4%	37.7%	65.4%
	**Total**		**no.**	3	52	75	130
			**%**	2.3%	40.0%	57.7%	100.0%
**07/40**	**position**	**building material**	**no.**	0	28	20	48
			**%**	0.0%	37.8%	27.0%	64.9%
		**surface**	**no.**	2	16	8	26
			**%**	2.7%	21.6%	10.8%	35.1%
	**Total**		**no.**	2	44	28	74
			**%**	2.7%	59.5%	37.8%	100.0%
**Total**	**position**	**building material**	**no.**	0	47	46	93
			**%**	0.0%	23.0%	22.5%	45.6%
		**surface**	**no.**	5	49	57	111
			**%**	2.5%	24.0%	27.9%	54.4%
	**Total**		**no.**	5	96	103	204
			**%**	2.5%	47.1%	50.5%	100.0%

**Fig 7:**
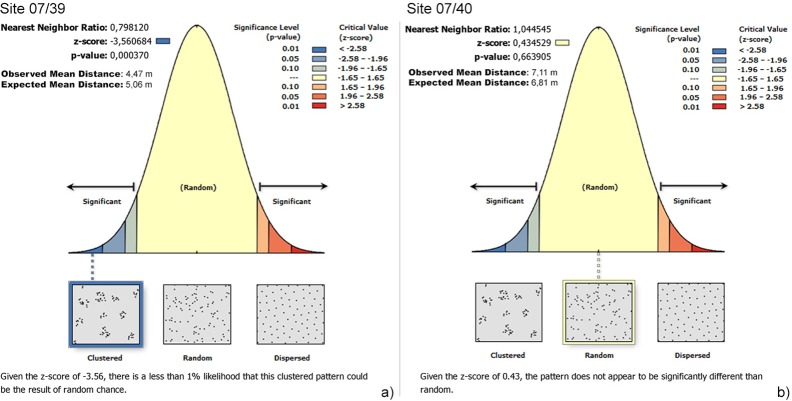
Results of Nearest Neighbour analyses of the distribution of TS on the surface. a) site 07/39; b) 07/40.

Site 07/40 presents a different and inverse ratio for the location of TS: 65% were found inside the stone structures (of non-cultic type) and in some cases had clearly been used as building materials (Table 8). The overall distribution pattern is less clustered than in 07/39; indeed, when we remove the TS found inside the structures from the sample, the remaining distribution is random all over the site (Fig 7). Furthermore, *opportunistic* TS prevail both for scattered items and for the TS found inside the stone structures.

**Fig 8:**
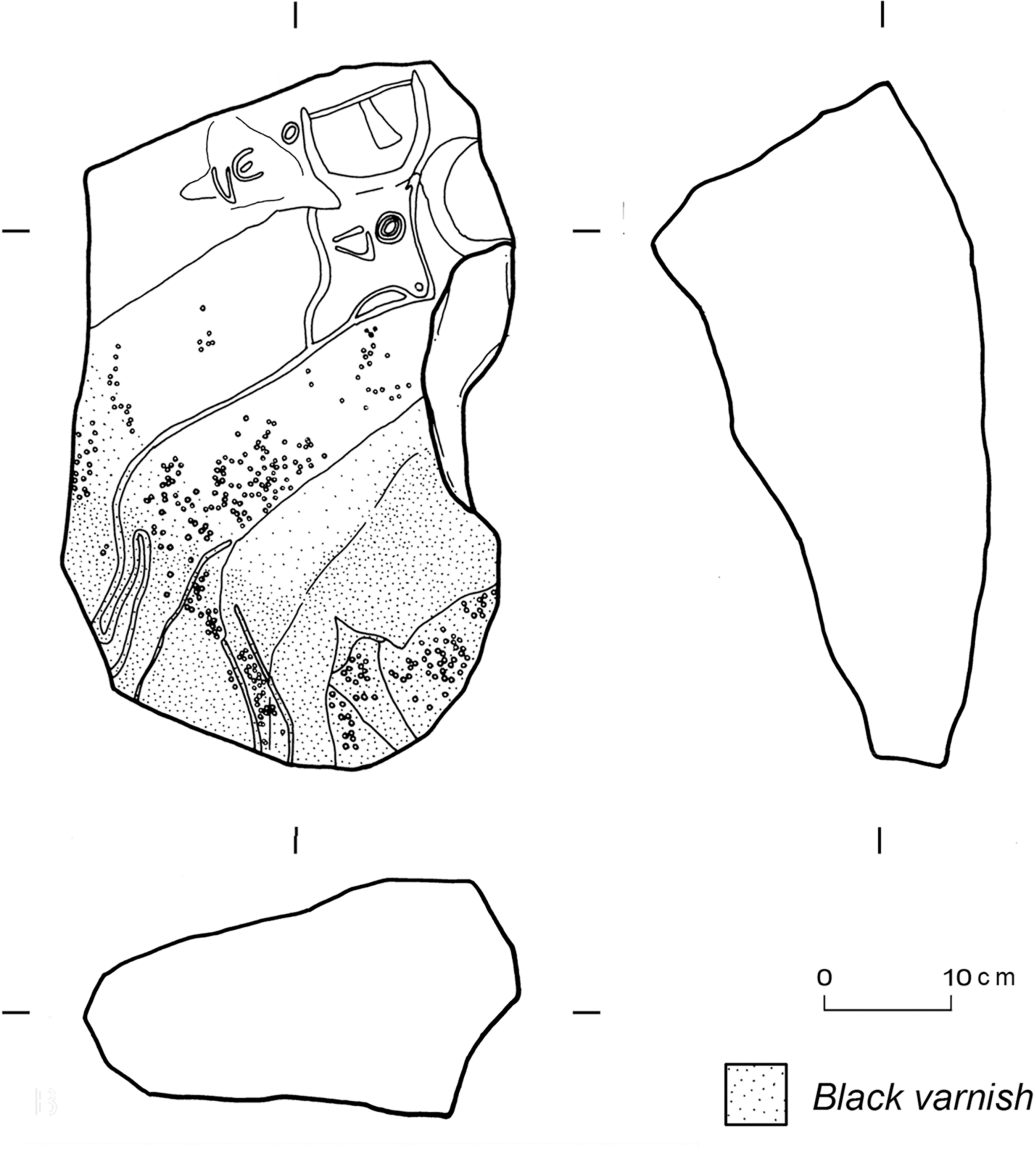
Example of TS with engravings from Site 07/39. The engraved tethering stone was found partially buried in *corbeille* C1 (modified, after [56]).

Comparing the TS embedded in the different types of stone structures, it is worth noting the high proportion of shaped TS in ceremonial structures (07/39), compared to domestic stone structures (07/40) where there is a high percentage of opportunistic TS. The prominent role of TS with a more defined shape as part of the *corbeille*-structures, where they are found in the perimeters and in the filling, seems to indicate a deliberate decision to use these artefacts in shaping the structure, as a building material but probably also for ritual or symbolic purposes. The presence of engraved TS in some of these structures supports this interpretation. This is true of a TS found in the filling of *corbeille* C1 (published as Feature C1/2 of Site 07/39 [56]). This TS was first decorated with a very fine three-dimensional representation of cattle engraved in profile using a bas-relief technique in a fully Pastoral style [56], then probably reused as a tethering stone—as suggested by the notch on the right-hand side cutting through the engraving of the heads of the two lower cows-, and finally incorporated into the monument as a building block with the upper part facing down, as is evident from the presence of black varnish only on the other exposed side (Fig 8).

There are also significant data on the TS located on the surface. The high frequencies and the high degree of clustering recorded in Site 07/39 suggest a use as a tethering tool for the cattle and other animals brought to the site for the ritual. This use is not visible in the sample from site 07/40, where a random distribution was recorded.

## Discussion

The data examined here represent the largest dataset of TS from a single region, the Messak Plateau, located in the heart of the Saharan desert.

**Fig 9:**
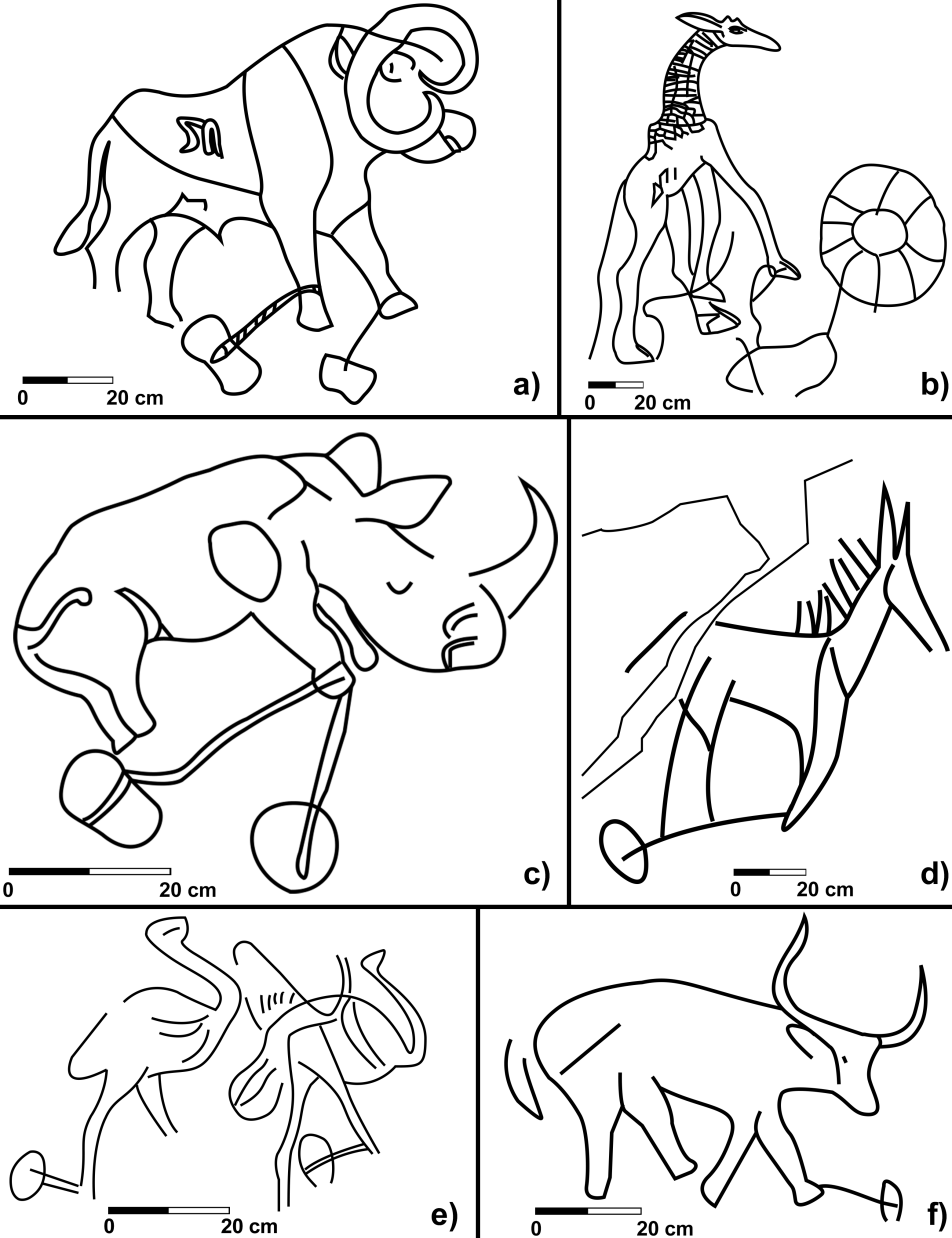
Rock art scenes with animals and TS. a) auroch; b) giraffe; c) rhino; d) equid; ostriches; domestic bovid.

Hitherto, overviews of these enigmatic features of the Saharan archaeological landscape have considered only limited data from a much larger region, encompassing most of the Sahara, and generally resulting from sporadic or unsystematic studies. Though previous studies have suggested a multifunctional use of TS (and the Ben Barour legend itself points to their use for tethering domestic camels…), their interpretation as hunting devices for large wild animals has prevailed. This interpretation is mainly based on their depiction in scenes interpreted as trap hunting in the art widespread throughout the Sahara; a significant concentration of such depictions is found precisely in the Messak plateau, and this use is supported by ethnographic sources. Here we do not discuss the iconographical repertoire in detail, as it will be the object of a specific paper; however, the rock art depictions already give us a visual indication of the multifunctional use of TS. In the Messak engravings, TS are associated with both wild game (and probably tamed animals) *and* domestic cattle (Fig 9).

The analysis performed on the TS recorded on the Messak plateau by a statistically-based survey, allows us: i. to identify a predominant distribution pattern of TS, indicating a significant correlation between these artefacts and some of the principal geomorphological features (endorheic depressions and specific areas of the desert pavement); ii. to suggest, on the basis of their varnish, the 3^rd^ mill. cal BC as the *final phase of production* of these artefacts, and, on the basis of their archaeological contexts, a widespread use during the Pastoral Neolithic; iii. to propose the coexistence of different functions. Indeed, our results do not rule out a use of TS as hunting devices, which still remains possible; a diversified use of TS during the Pastoral Neolithic is highly likely.

Endorheic depressions possibly led to a denser vegetation cover inside the rocky Holocene Messak that was suited both to hunting and to rearing cattle and small livestock. The widespread presence of TS likely associated with temporary Pastoral Neolithic campsites in endorheic areas supports an interpretation of TS basically as tethering tools for small and large grazing livestock. The prevalence of light and well-shaped TS perfectly fits this interpretation: the stones were carefully fashioned for repeated use and their weight may have served to tether animals rather than immobilize them.

The highest concentrations of TS occur in specific areas of the desert pavement, especially along the main meander of broad wadis, such as the Wadi Bedis in the centre of the Messak plateau. TS are found in ceremonial sites focused on the complex “cattle cult” rituals performed by herders residing in the Messak plateau during dry spells and dispersing towards the Murzuq lowlands in the wet season. The use of TS as hunting devices here must be ruled out. The numbers, density, features and locations of TS in sites 07/39 and 07/40 suggest three main uses: i. highly clustered concentrations of well-shaped TS used as tethering devices for domestic livestock, located in the vicinity of the ceremonial structures; ii. a functional use (or re-use) of TS as opportunistic building materials in everyday structures; iii. a possible ritual use of these artefacts in the context of ceremonial sites, as suggested by the careful selection of well-shaped TS as *corbeille* building materials, and by the presence of TS with engravings, embedded in the ceremonial structures, as in the case of *corbeille* 07/39-C1.

As noted for other ceremonial contexts in Egypt [71] and in our own research area [26], large numbers of people must have gathered for these feasting ceremonies and a very large quantity of meat must have been available. The congregation of different groups in the same ceremonial contexts is suggested by isotopic studies of the tooth enamel of slaughtered cattle, which come from very different places but are buried in the same monument [25]. The presence of large numbers of people and large numbers of animals in a specific place for a limited time may be mirrored precisely by the large number of TS, which, not coincidentally, are present in this type of location in the highest numbers and maximum concentrations.

Though we can probably assume a multifunctional use of TS in different chrono-cultural periods, for the Pastoral Neolithic we can tentatively suggest that we are dealing with a sort of *cultural exaptation* process. TS were probably made and used as hunting devices -as we can assume from the presence of isolated items, particularly of the *shaped* and *heavy grooved* types-, and then further modified and changed for other important purposes, such as the tethering of domestic cattle and small livestock, that represents the bulk of our dataset. This use also took on considerable symbolic significance, as the tethering of animals often appears to be closely related to the highly formalized ritual areas, where cattle were slaughtered, consumed and their remains deposited in stone ritual structures, like the *corbeilles*. The reuse of TS in *corbeilles* as building materials may be part of this ritual symbolism. Finally, TS were reused in rather late domestic stone structures, suggesting a basically opportunistic use despite a persistent marginal role as a hunting device that has persisted until the present e.g. [18,19].

## Conclusion

TS are an iconic feature of arid landscapes, particularly in the Saharan region, and constitute a set of widespread and still little investigated artefacts. A multifunctional use of TS during the Pastoral phases, mainly focused on the complex features associated with the management of herds and with Pastoral cultic activities, is supported by data collected throughout the Messak plateau. This interpretation suggests the need for a partial correction of their traditional identification as hunting devices. Our new interpretation is based on an integrated approach combining landscape and intra-site analyses: the correlations between specific attributes of TS and their location in the Messak environment and in different archaeological contexts allowed us to obtain a broader perspective on their changing function and ritual significance. The results may shed new light on these artefacts in other desert landscapes, in the Sahara as well as in Arabia, where TS have been recorded but not fully discussed. Indeed, recent data on the early Neolithic phases and the emergence of herding in Arabia revealing increasingly close connections with the Saharan archaeological landscape [72–75].

## Supporting information

S1 Appendix TS dataset. (PDF)
