# Supplementary material for: Trapping or tethering stones (TS): A multifunctional device in the Pastoral Neolithic of the Sahara
Source: PLoS One. 2018 Jan 25;13(1):e0191765. doi: 10.1371/journal.pone.0191765 (PMC5784975; doi:10.1371/journal.pone.0191765)
Supplement: S1 Appendix — (PDF) [file pone.0191765.s001.pdf]

i. Position and Topography								ii. TS features									iii. intra-site info	
ID_TS	name site	n. transect	project	lat.	long.	topography	geomorphology	lenght (cm)	width (cm)	thickness (cm)	n. notches	groove	weight (kg)	weight class*	rock varnish	shape	context	position
1	09/32	-	MCMP	25,9622	11,9452	hamada	desert pavement	42	20	17	2	no	33	3 = 26-50	black/black	shaped	isolated	isolated
2	07/34	-	MCMP	25,9904	11,9882	hamada	endorheic depression	37	32	13	2	no	35	3 = 26-50	black/red	shaped	campsite	surface
3	07/34	-	MCMP	25,9904	11,9881	hamada	endorheic depression	35	22	7	2	no	12	2 = 10-26	black/red	opportunistic	campsite	surface
4	07/34	-	MCMP	25,9903	11,9881	hamada	endorheic depression	32	20	6	1	no	9	1 =1-10	black/red	opportunistic	campsite	surface
5	07/34	-	MCMP	25,9903	11,9871	hamada	endorheic depression	32	25	18	2	no	33	3 = 26-50	black/red	shaped	campsite	surface
6	07/34	-	MCMP	25,9902	11,9872	hamada	endorheic depression	47	33	7	1	no	25	2 = 10-26	black/black	opportunistic	campsite	surface
7	07/34	-	MCMP	25,9902	11,9876	hamada	endorheic depression	48	29	16	1	no	51	4 = 50-85	black/red	opportunistic	campsite	surface
8	07/34	-	MCMP	25,9901	11,9880	hamada	endorheic depression	29	15	9	2	no	9	1 =1-10	black/red	shaped	campsite	surface
9	07/34	-	MCMP	25,9901	11,9884	hamada	endorheic depression	31	16	13	2	no	15	2 = 10-26	black/red	shaped	campsite	surface
10	07/34	-	MCMP	25,9901	11,9883	hamada	endorheic depression	38	22	12	1	light	23	2 = 10-26	black/red	shaped	campsite	surface
11	07/34	-	MCMP	25,9900	11,9880	hamada	endorheic depression	44	22	9	2	no	20	2 = 10-26	black/red	shaped	campsite	surface
12	07/34	-	MCMP	25,9900	11,9879	hamada	endorheic depression	50	27	7	1	no	22	2 = 10-26	black/red	shaped	campsite	surface
13	07/34	-	MCMP	25,9900	11,9879	hamada	endorheic depression	30	32	8	2	light	18	2 = 10-26	black/red	shaped	campsite	surface
14	07/34	-	MCMP	25,9900	11,9877	hamada	endorheic depression	40	30	23	2	light	63	4 = 50-85	black/red	shaped	campsite	surface
15	07/34	-	MCMP	25,9899	11,9882	hamada	endorheic depression	28	16	12	1	no	12	2 = 10-26	black/red	shaped	campsite	surface
16	07/34	-	MCMP	25,9899	11,9882	hamada	endorheic depression	30	19	9	2	no	12	2 = 10-26	black/red	shaped	campsite	surface
17	07/34	-	MCMP	25,9899	11,9877	hamada	endorheic depression	38	27	10	1	no	24	2 = 10-26	black/red	opportunistic	campsite	surface
18	07/34	-	MCMP	25,9899	11,9879	hamada	endorheic depression	37	12	5	1	no	5	1 =1-10	black/red	opportunistic	campsite	surface
19	07/34	-	MCMP	25,9899	11,9880	hamada	endorheic depression	50	22	13	2	no	33	3 = 26-50	black/red	opportunistic	campsite	surface
20	07/34	-	MCMP	25,9899	11,9880	hamada	endorheic depression	33	22	7	1	no	12	2 = 10-26	black/black	opportunistic	campsite	surface
21	07/34	-	MCMP	25,9899	11,9880	hamada	endorheic depression	36	24	12	1	no	24	2 = 10-26	black/red	opportunistic	campsite	surface
22	07/34	-	MCMP	25,9898	11,9878	hamada	endorheic depression	59	28	10	2	no	38	3 = 26-50	nd	shaped	campsite	surface
23	07/34	-	MCMP	25,9898	11,9878	hamada	endorheic depression	40	36	10	1	no	33	3 = 26-50	black/red	shaped	campsite	surface
24	07/34	-	MCMP	25,9898	11,9880	hamada	endorheic depression	35	12	6	1	no	6	1 =1-10	black/red	opportunistic	campsite	surface
25	07/34	-	MCMP	25,9898	11,9877	hamada	endorheic depression	46	42	16	2	no	71	4 = 50-85	black/red	shaped	campsite	surface
26	07/34	-	MCMP	25,9898	11,9879	hamada	endorheic depression	34	30	11	2	no	26	2 = 10-26	black/red	shaped	campsite	surface
27	07/34	-	MCMP	25,9898	11,9881	hamada	endorheic depression	54	30	14	2	no	52	4 = 50-85	black/red	opportunistic	campsite	surface
28	07/34	-	MCMP	25,9897	11,9879	hamada	endorheic depression	35	15	7	1	no	8	1 =1-10	black/red	opportunistic	campsite	surface
29	07/34	-	MCMP	25,9897	11,9879	hamada	endorheic depression	52	32	12	2	light	46	3 = 26-50	black/red	opportunistic	campsite	surface
30	07/34	-	MCMP	25,9897	11,9882	hamada	endorheic depression	25	18	7	2	no	7	1 =1-10	black/black	shaped	campsite	surface
31	07/34	-	MCMP	25,9897	11,9885	hamada	endorheic depression	37	27	12	2	no	28	3 = 26-50	black/red	shaped	campsite	surface
32	07/34	-	MCMP	25,9897	11,9880	hamada	endorheic depression	47	28	7	1	no	21	2 = 10-26	black/red	shaped	campsite	surface
33	07/34	-	MCMP	25,9897	11,9880	hamada	endorheic depression	44	22	23	2	no	51	4 = 50-85	black/red	opportunistic	campsite	surface
34	07/34	-	MCMP	25,9897	11,9882	hamada	endorheic depression	36	22	10	2	no	18	2 = 10-26	black/red	shaped	campsite	surface
35	07/34	-	MCMP	25,9896	11,9882	hamada	endorheic depression	55	29	7	2	no	26	2 = 10-26	black/red	opportunistic	campsite	surface
36	07/34	-	MCMP	25,9896	11,9881	hamada	endorheic depression	44	26	10	2	no	26	3 = 26-50	black/red	shaped	campsite	surface
37	07/34	-	MCMP	25,9896	11,9881	hamada	endorheic depression	55	20	18	1	no	46	3 = 26-50	black/red	opportunistic	campsite	surface
38	07/34	-	MCMP	25,9896	11,9875	hamada	endorheic depression	40	38	7	2	no	24	2 = 10-26	black/red	shaped	campsite	surface
39	07/34	-	MCMP	25,9895	11,9872	hamada	endorheic depression	31	16	10	1	no	11	2 = 10-26	black/red	shaped	campsite	surface
40	07/34	-	MCMP	25,9895	11,9876	hamada	endorheic depression	28	31	22	2	no	44	3 = 26-50	nd	shaped	campsite	surface
41	07/34	-	MCMP	25,9895	11,9876	hamada	endorheic depression	41	26	12	2	no	29	3 = 26-50	black/red	opportunistic	campsite	surface
42	07/34	-	MCMP	25,9895	11,9883	hamada	endorheic depression	50	28	12	2	no	39	3 = 26-50	black/red	shaped	campsite	surface
43	07/34	-	MCMP	25,9894	11,9875	hamada	endorheic depression	48	22	7	1	no	17	2 = 10-26	black/red	shaped	campsite	surface
44	07/34	-	MCMP	25,9894	11,9879	hamada	endorheic depression	33	18	15	2	no	20	2 = 10-26	black/red	opportunistic	campsite	surface
45	07/34	-	MCMP	25,9894	11,9883	hamada	endorheic depression	50	30	12	2	no	41	3 = 26-50	black/red	shaped	campsite	surface
46	07/34	-	MCMP	25,9894	11,9879	hamada	endorheic depression	42	20	22	1	no	43	3 = 26-50	black/red	opportunistic	campsite	surface
47	07/34	-	MCMP	25,9894	11,9884	hamada	endorheic depression	37	25	7	1	no	15	2 = 10-26	black/red	opportunistic	campsite	surface
48	07/34	-	MCMP	25,9894	11,9884	hamada	endorheic depression	47	21	14	2	no	32	3 = 26-50	black/red	shaped	campsite	surface
49	07/34	-	MCMP	25,9894	11,9883	hamada	endorheic depression	67	23	20	1	no	71	4 = 50-85	black/red	opportunistic	campsite	surface
50	07/34	-	MCMP	25,9894	11,9884	hamada	endorheic depression	45	21	15	1	no	33	3 = 26-50	black/red	shaped	campsite	surface
51	07/34	-	MCMP	25,9894	11,9879	hamada	endorheic depression	48	23	19	2	no	48	3 = 26-50	black/red	opportunistic	campsite	surface
52	07/34	-	MCMP	25,9894	11,9882	hamada	endorheic depression	43	31	12	2	no	37	3 = 26-50	black/red	shaped	campsite	surface
53	07/34	-	MCMP	25,9893	11,9882	hamada	endorheic depression	40	27	11	2	no	27	3 = 26-50	black/red	shaped	campsite	surface
54	07/34	-	MCMP	25,9892	11,9882	hamada	endorheic depression	73	28,5	13	2	no	62	4 = 50-85	black/red	shaped	campsite	surface
55	07/34	-	MCMP	25,9892	11,9877	hamada	endorheic depression	34	17	8	2	no	11	2 = 10-26	black/red	shaped	campsite	surface
56	07/34	-	MCMP	25,9891	11,9881	hamada	endorheic depression	34	27	10	1	no	21	2 = 10-26	black/red	opportunistic	campsite	surface
57	07/34	-	MCMP	25,9891	11,9883	hamada	endorheic depression	48	37	10	2	no	41	3 = 26-50	black/red	shaped	campsite	surface
58	07/34	-	MCMP	25,9887	11,9882	hamada	endorheic depression	48	23	8	1	no	20	2 = 10-26	black/red	shaped	campsite	surface
59	07/34	-	MCMP	25,9886	11,9881	hamada	endorheic depression	59	29	10	2	no	39	3 = 26-50	black/red	shaped	campsite	surface
60	07/35	-	MCMP	25,9906	11,9853	hamada	endorheic depression	48	37	17	2	circular	69	4 = 50-85	nd	heavy grooved	campsite	surface

61	07/35	-	MCMP	25,9906	11,9853	hamada	endorheic depression	0	0	0	2	no	0	nd	nd	nd	campsite	surface
62	07/35	-	MCMP	25,9906	11,9853	hamada	endorheic depression	0	0	0	2	no	0	nd	nd	nd	campsite	surface
63	07/35	-	MCMP	25,9906	11,9853	hamada	endorheic depression	0	0	0	2	no	0	nd	nd	nd	campsite	surface
64	07/35	-	MCMP	25,9906	11,9853	hamada	endorheic depression	0	0	0	2	no	0	nd	nd	nd	campsite	surface
65	07/35	-	MCMP	25,9906	11,9853	hamada	endorheic depression	0	0	0	2	no	0	nd	nd	nd	campsite	surface
66	07/35	-	MCMP	25,9906	11,9853	hamada	endorheic depression	0	0	0	2	no	0	nd	nd	nd	campsite	surface
67	07/35	-	MCMP	25,9906	11,9853	hamada	endorheic depression	0	0	0	2	no	0	nd	nd	nd	campsite	surface
68	07/35	-	MCMP	25,9906	11,9853	hamada	endorheic depression	0	0	0	2	no	0	nd	nd	nd	campsite	surface
69	07/35	-	MCMP	25,9906	11,9853	hamada	endorheic depression	0	0	0	2	no	0	nd	nd	nd	campsite	surface
70	07/35	-	MCMP	25,9906	11,9853	hamada	endorheic depression	0	0	0	2	no	0	nd	nd	nd	campsite	surface
71	07/35	-	MCMP	25,9906	11,9853	hamada	endorheic depression	0	0	0	2	no	0	nd	nd	nd	campsite	surface
72	07/35	-	MCMP	25,9906	11,9853	hamada	endorheic depression	0	0	0	2	no	0	nd	nd	nd	campsite	surface
73	07/35	-	MCMP	25,9906	11,9853	hamada	endorheic depression	0	0	0	2	no	0	nd	nd	nd	campsite	surface
74	07/35	-	MCMP	25,9906	11,9853	hamada	endorheic depression	0	0	0	2	no	0	nd	nd	nd	campsite	surface
75	07/35	-	MCMP	25,9906	11,9853	hamada	endorheic depression	0	0	0	2	no	0	nd	nd	nd	campsite	surface
76	07/35	-	MCMP	25,9906	11,9853	hamada	endorheic depression	0	0	0	2	no	0	nd	nd	nd	campsite	surface
77	07/35	-	MCMP	25,9906	11,9853	hamada	endorheic depression	0	0	0	2	no	0	nd	nd	nd	campsite	surface
78	07/36	-	MCMP	25,9658	11,9418	hamada	endorheic depression	0	0	0	2	circular	0	nd	black/red	heavy grooved	isolated	isolated
79	09/37	-	MCMP	25,9783	11,9419	hamada	desert pavement	40	27	20	2	no	50	3 = 26-50	black/red	nd	campsite	surface
80	09/37	-	MCMP	25,9782	11,9419	hamada	desert pavement	105	40	15	2	no	145	5 = 85-146	black/red	shaped	campsite	surface
81	09/37	-	MCMP	25,9782	11,9418	hamada	desert pavement	58	37	0	2	no	0	nd	nd	nd	campsite	surface
82	09/37	-	MCMP	25,9781	11,9418	hamada	desert pavement	60	19	6	1	no	16	2 = 10-26	black/red	nd	campsite	surface
83	09/37	-	MCMP	25,9781	11,9419	hamada	desert pavement	42	40	10	2	no	39	3 = 26-50	black/red	shaped	campsite	surface
84	09/37	-	MCMP	25,9781	11,9419	hamada	desert pavement	50	40	16	2	no	74	4 = 50-85	black/red	shaped	campsite	surface
85	09/37	-	MCMP	25,9781	11,9420	hamada	desert pavement	50	20	12	2	no	28	3 = 26-50	black/black	opportunistic	campsite	surface
86	09/37	-	MCMP	25,9781	11,9421	hamada	desert pavement	52	35	18	2	no	75	4 = 50-85	black/red	shaped	campsite	surface
87	09/37	-	MCMP	25,9781	11,9419	hamada	desert pavement	57	23	13	2	no	39	3 = 26-50	black/black	shaped	campsite	surface
88	09/37	-	MCMP	25,9780	11,9422	hamada	desert pavement	60	15	15	2	no	31	3 = 26-50	black/red	opportunistic	campsite	surface
89	09/37	-	MCMP	25,9780	11,9421	hamada	desert pavement	60	37	10	2	no	51	4 = 50-85	black/red	shaped	campsite	surface
90	09/37	-	MCMP	25,9779	11,9421	hamada	desert pavement	50	35	5	2	no	20	2 = 10-26	black/red	shaped	campsite	surface
91	09/37	-	MCMP	25,9778	11,9418	hamada	desert pavement	50	15	16	2	no	28	3 = 26-50	black/red	shaped	campsite	surface
92	09/37	-	MCMP	25,9778	11,9422	hamada	desert pavement	50	30	15	2	no	52	4 = 50-85	black/red	opportunistic	campsite	surface
93	09/37	-	MCMP	25,9778	11,9423	hamada	desert pavement	65	20	15	2	no	45	3 = 26-50	black/red	shaped	campsite	surface
94	09/37	-	MCMP	25,9778	11,9422	hamada	desert pavement	64	38	15	2	no	84	4 = 50-85	black/red	opportunistic	campsite	surface
95	09/37	-	MCMP	25,9778	11,9422	hamada	desert pavement	65	20	10	2	no	30	3 = 26-50	black/red	shaped	campsite	surface
96	09/37	-	MCMP	25,9778	11,9421	hamada	desert pavement	83	35	13	2	no	87	5 = 85-146	black/red	shaped	campsite	surface
97	09/37	-	MCMP	25,9778	11,9418	hamada	desert pavement	60	30	10	1	no	41	3 = 26-50	black/black	opportunistic	campsite	surface
98	09/37	-	MCMP	25,9778	11,9418	hamada	desert pavement	44	28	12	2	no	34	3 = 26-50	black/red	nd	campsite	surface
99	09/37	-	MCMP	25,9778	11,9418	hamada	desert pavement	33	21	18	2	no	29	3 = 26-50	black/black	shaped	campsite	surface
100	09/37	-	MCMP	25,9777	11,9421	hamada	desert pavement	55	35	12	2	no	53	4 = 50-85	black/red	shaped	campsite	surface
101	09/37	-	MCMP	25,9777	11,9422	hamada	desert pavement	60	25	18	2	no	62	4 = 50-85	black/black	opportunistic	campsite	surface
102	09/37	-	MCMP	25,9777	11,9419	hamada	desert pavement	45	32	9	2	no	30	3 = 26-50	black/black	shaped	campsite	surface
103	09/37	-	MCMP	25,9777	11,9418	hamada	desert pavement	60	20	23	2	no	63	4 = 50-85	black/black	shaped	campsite	surface
104	09/37	-	MCMP	25,9777	11,9418	hamada	desert pavement	53	30	18	2	no	66	4 = 50-85	black/black	shaped	campsite	surface
105	09/37	-	MCMP	25,9776	11,9420	hamada	desert pavement	54	28	19	2	no	66	4 = 50-85	black/black	nd	campsite	surface
106	07/39	-	MCMP	25,9987	11,9775	hamada	desert pavement	79	37	15	2	no	101	5 = 85-146	black/black	shaped	ceremonial/funerary context	surface
107	07/39	-	MCMP	25,9985	11,9777	hamada	desert pavement	54	33	7	2	no	29	3 = 26-50	black/black	shaped	ceremonial/funerary context	surface
108	07/39	-	MCMP	25,9984	11,9774	hamada	desert pavement	46	36	8	1	no	30	3 = 26-50	black/red	opportunistic	ceremonial/funerary context	surface
109	07/39	-	MCMP	25,9984	11,9779	hamada	desert pavement	57	30	9	2	no	35	3 = 26-50	black/black	opportunistic	ceremonial/funerary context	surface
110	07/39	-	MCMP	25,9983	11,9774	hamada	desert pavement	61	34	10	2	no	48	3 = 26-50	black/black	opportunistic	ceremonial/funerary context	surface
111	07/39	-	MCMP	25,9983	11,9773	hamada	desert pavement	48	42	9	2	no	42	3 = 26-50	black/red	opportunistic	ceremonial/funerary context	surface
112	07/39	-	MCMP	25,9982	11,9775	hamada	desert pavement	52	43	9	2	no	46	3 = 26-50	black/red	opportunistic	ceremonial/funerary context	surface
113	07/39	-	MCMP	25,9982	11,9779	hamada	desert pavement	0	0	0	3	no	0	nd	black/red	shaped	ceremonial/funerary structure	building material
114	07/39	-	MCMP	25,9982	11,9776	hamada	desert pavement	34	26	12	2	no	24	2 = 10-26	black/red	opportunistic	ceremonial/funerary context	surface
115	07/39	-	MCMP	25,9982	11,9779	hamada	desert pavement	50	42	9	2	no	43	3 = 26-50	black/black	shaped	ceremonial/funerary context	surface
116	07/39	-	MCMP	25,9982	11,9780	hamada	desert pavement	46	34	10	2	no	36	3 = 26-50	black/black	opportunistic	ceremonial/funerary context	surface
117	07/39	-	MCMP	25,9982	11,9782	hamada	desert pavement	60	32	11	2	no	49	3 = 26-50	black/black	opportunistic	ceremonial/funerary context	surface
118	07/39	-	MCMP	25,9982	11,9782	hamada	desert pavement	46	34	12	2	no	43	3 = 26-50	black/black	opportunistic	ceremonial/funerary structure	building material
119	07/39	-	MCMP	25,9982	11,9783	hamada	desert pavement	46	25	12	1	light	32	3 = 26-50	black/black	shaped	ceremonial/funerary context	surface
120	07/39	-	MCMP	25,9982	11,9775	hamada	desert pavement	51	43	7	2	no	35	3 = 26-50	black/red	shaped	ceremonial/funerary context	surface
121	07/39	-	MCMP	25,9982	11,9775	hamada	desert pavement	62	36	17	2	no	87	5 = 85-146	black/red	opportunistic	ceremonial/funerary context	surface
122	07/39	-	MCMP	25,9981	11,9778	hamada	desert pavement	48	25	5	2	no	14	2 = 10-26	black/red	opportunistic	ceremonial/funerary context	surface
123	07/39	-	MCMP	25,9981	11,9778	hamada	desert pavement	63	38	13	2	no	72	4 = 50-85	black/red	shaped	ceremonial/funerary structure	building material

124	07/39	-	MCMP	25,9981	11,9778	hamada	desert pavement	39	0	8	2	no	0	nd	black/red	shaped	ceremonial/funerary structure	building material
125	07/39	-	MCMP	25,9981	11,9774	hamada	desert pavement	62	50	11	2	no	78	4 = 50-85	black/red	shaped	ceremonial/funerary context	surface
126	07/39	-	MCMP	25,9981	11,9778	hamada	desert pavement	0	0	0	2	no	0	nd	black/red	shaped	ceremonial/funerary structure	building material
127	07/39	-	MCMP	25,9981	11,9778	hamada	desert pavement	58	42	7	3	no	39	3 = 26-50	black/red	opportunistic	ceremonial/funerary structure	building material
128	07/39	-	MCMP	25,9981	11,9777	hamada	desert pavement	53	39	19	2	no	90	5 = 85-146	black/red	opportunistic	ceremonial/funerary context	surface
129	07/39	-	MCMP	25,9981	11,9777	hamada	desert pavement	60	29	13	2	no	52	4 = 50-85	black/black	shaped	ceremonial/funerary context	surface
130	07/39	-	MCMP	25,9981	11,9778	hamada	desert pavement	0	0	0	2	no	0	nd	black/red	shaped	ceremonial/funerary structure	building material
131	07/39	-	MCMP	25,9981	11,9778	hamada	desert pavement	80	53	14	3	no	137	5 = 85-146	black/black	shaped	ceremonial/funerary structure	building material
132	07/39	-	MCMP	25,9981	11,9778	hamada	desert pavement	60	31	15	2	no	64	4 = 50-85	black/red	shaped	ceremonial/funerary structure	building material
133	07/39	-	MCMP	25,9981	11,9779	hamada	desert pavement	61	14	12	2	no	24	2 = 10-26	black/black	opportunistic	ceremonial/funerary context	surface
134	07/39	-	MCMP	25,9981	11,9779	hamada	desert pavement	43	35	11	4	no	38	3 = 26-50	black/red	opportunistic	ceremonial/funerary context	surface
135	07/39	-	MCMP	25,9981	11,9778	hamada	desert pavement	60	24	10	4	no	33	3 = 26-50	black/red	shaped	ceremonial/funerary structure	building material
136	07/39	-	MCMP	25,9981	11,9778	hamada	desert pavement	65	0	7	2	no	0	nd	black/red	shaped	ceremonial/funerary structure	building material
137	07/39	-	MCMP	25,9981	11,9778	hamada	desert pavement	63	37	14	4	no	75	4 = 50-85	black/red	opportunistic	ceremonial/funerary structure	building material
138	07/39	-	MCMP	25,9981	11,9778	hamada	desert pavement	0	0	0	2	no	0	nd	black/red	opportunistic	ceremonial/funerary structure	building material
139	07/39	-	MCMP	25,9981	11,9778	hamada	desert pavement	0	0	0	2	no	0	nd	black/red	opportunistic	ceremonial/funerary structure	building material
140	07/39	-	MCMP	25,9981	11,9774	hamada	desert pavement	38	25	9	2	no	20	2 = 10-26	black/black	opportunistic	ceremonial/funerary context	surface
141	07/39	-	MCMP	25,9981	11,9778	hamada	desert pavement	78	0	8	2	no	0	nd	black/red	shaped	ceremonial/funerary structure	building material
142	07/39	-	MCMP	25,9981	11,9778	hamada	desert pavement	54	22	12	3	no	33	3 = 26-50	black/red	shaped	ceremonial/funerary structure	building material
143	07/39	-	MCMP	25,9981	11,9778	hamada	desert pavement	0	0	8	2	no	0	nd	black/red	shaped	ceremonial/funerary structure	building material
144	07/39	-	MCMP	25,9981	11,9777	hamada	desert pavement	0	0	14	2	no	0	nd	black/red	opportunistic	ceremonial/funerary structure	building material
145	07/39	-	MCMP	25,9981	11,9779	hamada	desert pavement	45	29	7	2	no	21	2 = 10-26	black/red	opportunistic	ceremonial/funerary context	surface
146	07/39	-	MCMP	25,9981	11,9778	hamada	desert pavement	0	0	11	2	no	0	nd	black/red	shaped	ceremonial/funerary structure	building material
147	07/39	-	MCMP	25,9981	11,9778	hamada	desert pavement	0	0	0	2	no	0	nd	black/red	opportunistic	ceremonial/funerary structure	building material
148	07/39	-	MCMP	25,9981	11,9777	hamada	desert pavement	0	0	15	2	no	0	nd	black/red	opportunistic	ceremonial/funerary structure	building material
149	07/39	-	MCMP	25,9981	11,9778	hamada	desert pavement	60	34	24	2	no	113	5 = 85-146	black/black	shaped	ceremonial/funerary structure	building material
150	07/39	-	MCMP	25,9981	11,9778	hamada	desert pavement	70	28	9	4	no	41	3 = 26-50	black/black	opportunistic	ceremonial/funerary structure	building material
151	07/39	-	MCMP	25,9981	11,9777	hamada	desert pavement	0	0	9	2	no	0	nd	black/black	shaped	ceremonial/funerary structure	building material
152	07/39	-	MCMP	25,9980	11,9776	hamada	desert pavement	69	31	15	3	no	74	4 = 50-85	black/black	shaped	ceremonial/funerary context	surface
153	07/39	-	MCMP	25,9980	11,9778	hamada	desert pavement	0	0	6	2	no	0	nd	black/red	shaped	ceremonial/funerary structure	building material
154	07/39	-	MCMP	25,9980	11,9778	hamada	desert pavement	62	37	21	2	no	111	5 = 85-146	black/red	shaped	ceremonial/funerary structure	building material
155	07/39	-	MCMP	25,9980	11,9776	hamada	desert pavement	37	34	6	2	light	17	2 = 10-26	black/red	opportunistic	ceremonial/funerary context	surface
156	07/39	-	MCMP	25,9980	11,9777	hamada	desert pavement	61	32	12	4	no	54	4 = 50-85	black/red	shaped	ceremonial/funerary context	surface
157	07/39	-	MCMP	25,9980	11,9777	hamada	desert pavement	53	20	6	2	no	15	2 = 10-26	black/red	shaped	ceremonial/funerary context	surface
158	07/39	-	MCMP	25,9980	11,9777	hamada	desert pavement	59	31	9	2	no	38	3 = 26-50	black/red	shaped	ceremonial/funerary structure	building material
159	07/39	-	MCMP	25,9980	11,9775	hamada	desert pavement	48	23	16	2	no	41	3 = 26-50	black/black	shaped	ceremonial/funerary context	surface
160	07/39	-	MCMP	25,9980	11,9777	hamada	desert pavement	69	49	8	2	no	62	4 = 50-85	black/black	opportunistic	ceremonial/funerary structure	building material
161	07/39	-	MCMP	25,9980	11,9776	hamada	desert pavement	62	27	13	4	no	50	3 = 26-50	black/red	shaped	ceremonial/funerary context	surface
162	07/39	-	MCMP	25,9980	11,9776	hamada	desert pavement	52	29	8	2	no	28	3 = 26-50	nd	opportunistic	ceremonial/funerary context	surface
163	07/39	-	MCMP	25,9980	11,9779	hamada	desert pavement	0	0	0	2	no	0	nd	black/black	shaped	ceremonial/funerary context	surface
164	07/39	-	MCMP	25,9980	11,9774	hamada	desert pavement	43	18	5	2	light	9	1 =1-10	black/black	opportunistic	ceremonial/funerary structure	building material
165	07/39	-	MCMP	25,9980	11,9774	hamada	desert pavement	62	40	12	2	no	68	4 = 50-85	black/black	opportunistic	ceremonial/funerary structure	building material
166	07/39	-	MCMP	25,9980	11,9775	hamada	desert pavement	60	48	13	2	no	86	5 = 85-146	black/red	shaped	campsite	building material
167	07/39	-	MCMP	25,9980	11,9776	hamada	desert pavement	52	22	11	2	no	29	3 = 26-50	black/red	shaped	ceremonial/funerary context	surface
168	07/39	-	MCMP	25,9980	11,9779	hamada	desert pavement	51	31	20	2	no	73	4 = 50-85	black/red	opportunistic	ceremonial/funerary structure	building material
169	07/39	-	MCMP	25,9980	11,9779	hamada	desert pavement	71	32	13	2	no	68	4 = 50-85	black/black	opportunistic	ceremonial/funerary structure	building material
170	07/39	-	MCMP	25,9980	11,9777	hamada	desert pavement	52	27	9	3	no	29	3 = 26-50	black/red	opportunistic	ceremonial/funerary context	surface
171	07/39	-	MCMP	25,9980	11,9779	hamada	desert pavement	60	29	13	2	no	52	4 = 50-85	black/black	shaped	ceremonial/funerary structure	building material
172	07/39	-	MCMP	25,9980	11,9779	hamada	desert pavement	87	46	10	2	no	92	5 = 85-146	black/red	shaped	ceremonial/funerary structure	building material
173	07/39	-	MCMP	25,9980	11,9779	hamada	desert pavement	59	33	17	3	no	76	4 = 50-85	black/red	shaped	ceremonial/funerary structure	building material
174	07/39	-	MCMP	25,9980	11,9779	hamada	desert pavement	72	30	5	2	no	25	2 = 10-26	black/red	shaped	ceremonial/funerary structure	building material
175	07/39	-	MCMP	25,9980	11,9779	hamada	desert pavement	56	18	15	2	no	35	3 = 26-50	black/black	shaped	ceremonial/funerary structure	building material
176	07/39	-	MCMP	25,9980	11,9779	hamada	desert pavement	51	31	10	2	no	36	3 = 26-50	black/red	shaped	ceremonial/funerary structure	building material
177	07/39	-	MCMP	25,9980	11,9779	hamada	desert pavement	59	33	17	2	no	76	4 = 50-85	black/red	opportunistic	ceremonial/funerary structure	building material
178	07/39	-	MCMP	25,9980	11,9779	hamada	desert pavement	75	36	13	2	no	81	4 = 50-85	black/red	shaped	ceremonial/funerary structure	building material
179	07/39	-	MCMP	25,9980	11,9779	hamada	desert pavement	0	0	0	2	no	0	nd	black/red	opportunistic	ceremonial/funerary structure	building material
180	07/39	-	MCMP	25,9980	11,9779	hamada	desert pavement	76	49	9	2	no	77	4 = 50-85	black/red	opportunistic	ceremonial/funerary structure	building material
181	07/39	-	MCMP	25,9980	11,9779	hamada	desert pavement	94	50	11	2	no	119	5 = 85-146	black/black	opportunistic	ceremonial/funerary structure	building material
182	07/39	-	MCMP	25,9980	11,9779	hamada	desert pavement	0	0	0	2	no	0	nd	black/red	opportunistic	ceremonial/funerary structure	building material
183	07/39	-	MCMP	25,9979	11,9780	hamada	desert pavement	78	36	15	2	no	97	5 = 85-146	black/red	shaped	ceremonial/funerary context	surface
184	07/39	-	MCMP	25,9979	11,9777	hamada	desert pavement	54	40	8	2	no	40	3 = 26-50	nd	opportunistic	ceremonial/funerary context	surface
185	07/39	-	MCMP	25,9979	11,9779	hamada	desert pavement	55	39	11	2	no	54	4 = 50-85	black/red	opportunistic	ceremonial/funerary context	surface
186	07/39	-	MCMP	25,9979	11,9777	hamada	desert pavement	33	27	9	1	no	18	2 = 10-26	black/black	opportunistic	ceremonial/funerary context	surface

187	07/39	-	MCMP	25,9979	11,9777	hamada	desert pavement	40	30	10	2	no	28	3 = 26-50	black/red	shaped	ceremonial/funerary context	surface
188	07/39	-	MCMP	25,9979	11,9777	hamada	desert pavement	23	22	8	4	no	9	1 =1-10	black/red	shaped	ceremonial/funerary context	surface
189	07/39	-	MCMP	25,9979	11,9777	hamada	desert pavement	60	18	15	2	no	37	3 = 26-50	black/red	opportunistic	ceremonial/funerary context	surface
190	07/39	-	MCMP	25,9979	11,9778	hamada	desert pavement	73	53	7	1	no	62	4 = 50-85	nd	opportunistic	ceremonial/funerary context	surface
191	07/39	-	MCMP	25,9979	11,9777	hamada	desert pavement	63	34	13	2	no	64	4 = 50-85	black/red	opportunistic	ceremonial/funerary context	surface
192	07/39	-	MCMP	25,9979	11,9777	hamada	desert pavement	63	38	14	2	no	77	4 = 50-85	black/red	opportunistic	ceremonial/funerary context	surface
193	07/39	-	MCMP	25,9979	11,9777	hamada	desert pavement	57	35	10	3	no	46	3 = 26-50	black/red	shaped	ceremonial/funerary context	surface
194	07/39	-	MCMP	25,9979	11,9777	hamada	desert pavement	57	33	12	2	no	52	4 = 50-85	black/red	shaped	ceremonial/funerary context	surface
195	07/39	-	MCMP	25,9979	11,9777	hamada	desert pavement	54	32	13	2	no	52	4 = 50-85	black/red	opportunistic	ceremonial/funerary context	surface
196	07/39	-	MCMP	25,9979	11,9776	hamada	desert pavement	40	30	9	2	no	25	2 = 10-26	black/red	shaped	ceremonial/funerary context	surface
197	07/39	-	MCMP	25,9979	11,9777	hamada	desert pavement	42	37	20	2	no	71	4 = 50-85	black/red	opportunistic	ceremonial/funerary context	surface
198	07/39	-	MCMP	25,9979	11,9777	hamada	desert pavement	45	25	9	2	no	23	2 = 10-26	black/red	shaped	ceremonial/funerary context	surface
199	07/39	-	MCMP	25,9979	11,9777	hamada	desert pavement	59	58	12	2	no	94	5 = 85-146	black/red	shaped	ceremonial/funerary context	surface
200	07/39	-	MCMP	25,9979	11,9776	hamada	desert pavement	67	23	13	2	no	46	3 = 26-50	black/red	shaped	ceremonial/funerary context	surface
201	07/39	-	MCMP	25,9979	11,9784	hamada	desert pavement	57	25	10	2	light	33	3 = 26-50	black/red	shaped	ceremonial/funerary context	surface
202	07/39	-	MCMP	25,9979	11,9782	hamada	desert pavement	63	37	10	2	no	54	4 = 50-85	black/red	shaped	ceremonial/funerary context	surface
203	07/39	-	MCMP	25,9979	11,9776	hamada	desert pavement	58	35	10	2	no	47	3 = 26-50	black/red	shaped	ceremonial/funerary context	surface
204	07/39	-	MCMP	25,9979	11,9785	hamada	desert pavement	56	29	9	2	no	34	3 = 26-50	black/black	opportunistic	ceremonial/funerary context	surface
205	07/39	-	MCMP	25,9978	11,9779	hamada	desert pavement	91	38	15	2	circular	119	5 = 85-146	nd	heavy grooved	ceremonial/funerary context	surface
206	07/39	-	MCMP	25,9978	11,9783	hamada	desert pavement	53	38	13	1	no	60	4 = 50-85	black/black	shaped	ceremonial/funerary context	surface
207	07/39	-	MCMP	25,9978	11,9785	hamada	desert pavement	71	24	10	2	no	39	3 = 26-50	nd	opportunistic	ceremonial/funerary context	surface
208	07/39	-	MCMP	25,9978	11,9786	hamada	desert pavement	48	34	7	3	no	26	3 = 26-50	black/red	shaped	ceremonial/funerary context	surface
209	07/39	-	MCMP	25,9978	11,9784	hamada	desert pavement	66	35	0	4	no	0	nd	black/red	shaped	ceremonial/funerary context	surface
210	07/39	-	MCMP	25,9978	11,9786	hamada	desert pavement	68	38	12	2	no	71	4 = 50-85	nd	shaped	ceremonial/funerary context	surface
211	07/39	-	MCMP	25,9978	11,9785	hamada	desert pavement	69	27	10	5	no	43	3 = 26-50	black/red	shaped	ceremonial/funerary context	surface
212	07/39	-	MCMP	25,9978	11,9785	hamada	desert pavement	48	32	9	2	no	32	3 = 26-50	black/black	shaped	ceremonial/funerary context	surface
213	07/39	-	MCMP	25,9978	11,9777	hamada	desert pavement	45	29	14	4	no	42	3 = 26-50	black/red	shaped	ceremonial/funerary context	surface
214	07/39	-	MCMP	25,9978	11,9777	hamada	desert pavement	44	22	9	2	no	20	2 = 10-26	black/red	shaped	ceremonial/funerary context	surface
215	07/39	-	MCMP	25,9978	11,9785	hamada	desert pavement	58	44	9	3	no	53	4 = 50-85	black/red	shaped	ceremonial/funerary context	surface
216	07/39	-	MCMP	25,9978	11,9778	hamada	desert pavement	60	34	24	2	no	113	5 = 85-146	black/red	shaped	ceremonial/funerary context	surface
217	07/39	-	MCMP	25,9978	11,9783	hamada	desert pavement	66	43	14	4	no	91	5 = 85-146	black/black	shaped	ceremonial/funerary context	surface
218	07/39	-	MCMP	25,9978	11,9781	hamada	desert pavement	60	35	13	2	no	63	4 = 50-85	black/red	shaped	ceremonial/funerary context	surface
219	07/39	-	MCMP	25,9978	11,9781	hamada	desert pavement	66	50	8	2	no	61	4 = 50-85	black/black	shaped	ceremonial/funerary context	surface
220	07/39	-	MCMP	25,9978	11,9777	hamada	desert pavement	41	32	8	2	no	24	2 = 10-26	black/red	opportunistic	ceremonial/funerary context	surface
221	07/39	-	MCMP	25,9978	11,9781	hamada	desert pavement	81	26	11	2	light	53	4 = 50-85	black/red	shaped	ceremonial/funerary context	surface
222	07/39	-	MCMP	25,9978	11,9778	hamada	desert pavement	79	53	14	4	no	135	5 = 85-146	black/red	shaped	ceremonial/funerary context	surface
223	07/39	-	MCMP	25,9978	11,9781	hamada	desert pavement	46	24	14	2	circular	36	3 = 26-50	black/red	heavy grooved	ceremonial/funerary context	surface
224	07/39	-	MCMP	25,9978	11,9778	hamada	desert pavement	43	28	22	2	no	61	4 = 50-85	black/red	shaped	ceremonial/funerary context	surface
225	07/39	-	MCMP	25,9978	11,9778	hamada	desert pavement	59	48	6	4	no	39	3 = 26-50	black/black	shaped	ceremonial/funerary context	surface
226	07/39	-	MCMP	25,9978	11,9782	hamada	desert pavement	62	31	23	2	circular	102	5 = 85-146	black/red	heavy grooved	ceremonial/funerary context	surface
227	07/39	-	MCMP	25,9978	11,9781	hamada	desert pavement	85	41	9	2	no	72	4 = 50-85	black/red	shaped	ceremonial/funerary context	surface
228	07/39	-	MCMP	25,9978	11,9777	hamada	desert pavement	53	28	12	2	no	41	3 = 26-50	black/black	opportunistic	ceremonial/funerary context	surface
229	07/39	-	MCMP	25,9978	11,9777	hamada	desert pavement	35	30	12	2	no	29	3 = 26-50	black/red	opportunistic	ceremonial/funerary context	surface
230	07/39	-	MCMP	25,9978	11,9781	hamada	desert pavement	36	28	12	2	no	28	3 = 26-50	black/red	shaped	ceremonial/funerary context	surface
231	07/39	-	MCMP	25,9977	11,9782	hamada	desert pavement	53	41	9,5	2	no	47	3 = 26-50	black/red	shaped	ceremonial/funerary context	surface
232	07/39	-	MCMP	25,9977	11,9783	hamada	desert pavement	50	30	13	2	no	45	3 = 26-50	black/red	shaped	ceremonial/funerary context	surface
233	07/39	-	MCMP	25,9977	11,9777	hamada	desert pavement	66	36	9	4	no	49	3 = 26-50	black/black	opportunistic	ceremonial/funerary context	surface
234	07/39	-	MCMP	25,9977	11,9778	hamada	desert pavement	57	46	14	2	no	84	4 = 50-85	black/red	shaped	ceremonial/funerary context	surface
235	07/39	-	MCMP	25,9977	11,9779	hamada	desert pavement	38	28	11	2	no	27	3 = 26-50	black/red	shaped	ceremonial/funerary context	surface
236	07/40	-	MCMP	25,9984	11,9770	hamada	desert pavement	76	46	12	2	no	96	5 = 85-146	black/red	opportunistic	campsite	surface
237	07/40	-	MCMP	25,9983	11,9768	hamada	desert pavement	43	26	7	1	no	18	2 = 10-26	black/black	opportunistic	campsite	surface
238	07/40	-	MCMP	25,9983	11,9771	hamada	desert pavement	33	10	5	1	light	4	1 =1-10	black/black	opportunistic	campsite	surface
239	07/40	-	MCMP	25,9983	11,9769	hamada	desert pavement	36	20	8	2	no	13	2 = 10-26	black/black	opportunistic	campsite	surface
240	07/40	-	MCMP	25,9983	11,9769	hamada	desert pavement	23	17	17	1	no	15	2 = 10-26	black/black	shaped	campsite	building material
241	07/40	-	MCMP	25,9982	11,9771	hamada	desert pavement	54	26	5	2	no	16	2 = 10-26	black/black	shaped	campsite	surface
242	07/40	-	MCMP	25,9982	11,9771	hamada	desert pavement	53	27	8	1	no	26	3 = 26-50	black/black	opportunistic	campsite	surface
243	07/40	-	MCMP	25,9982	11,9766	hamada	desert pavement	101	49	10	2	no	114	5 = 85-146	black/black	shaped	campsite	surface
244	07/40	-	MCMP	25,9982	11,9770	hamada	desert pavement	41	34	10	1	no	32	3 = 26-50	black/black	opportunistic	campsite	building material
245	07/40	-	MCMP	25,9982	11,9770	hamada	desert pavement	57	48	17	3	no	107	5 = 85-146	black/black	opportunistic	campsite	building material
246	07/40	-	MCMP	25,9982	11,9771	hamada	desert pavement	48	25	11	2	light	30	3 = 26-50	black/black	shaped	campsite	building material
247	07/40	-	MCMP	25,9982	11,9768	hamada	desert pavement	40	26	10	2	no	24	2 = 10-26	black/black	opportunistic	campsite	building material
248	07/40	-	MCMP	25,9982	11,9771	hamada	desert pavement	69	25	9	2	no	36	3 = 26-50	nd	shaped	campsite	building material
249	07/40	-	MCMP	25,9982	11,9773	hamada	desert pavement	53	28	7	2	no	24	2 = 10-26	black/black	opportunistic	campsite	surface

250	07/40	-	MCMP	25,9981	11,9773	hamada	desert pavement	57	15	10	1	no	20	2 = 10-26	black/black	opportunistic	campsite	surface
251	07/40	-	MCMP	25,9981	11,9773	hamada	desert pavement	43	34	10	2	no	34	3 = 26-50	black/red	shaped	campsite	building material
252	07/40	-	MCMP	25,9981	11,9767	hamada	desert pavement	27	17	4	2	no	4	1 =1-10	black/black	shaped	campsite	surface
253	07/40	-	MCMP	25,9981	11,9770	hamada	desert pavement	43	25	16	4	no	40	3 = 26-50	black/black	opportunistic	campsite	building material
254	07/40	-	MCMP	25,9981	11,9773	hamada	desert pavement	69	31	11	3	no	54	4 = 50-85	black/red	opportunistic	campsite	building material
255	07/40	-	MCMP	25,9981	11,9773	hamada	desert pavement	35	30	9	4	no	22	2 = 10-26	black/red	shaped	campsite	surface
256	07/40	-	MCMP	25,9981	11,9771	hamada	desert pavement	56	40	10	1	no	52	4 = 50-85	black/black	opportunistic	campsite	surface
257	07/40	-	MCMP	25,9981	11,9771	hamada	desert pavement	48	30	9	2	no	30	3 = 26-50	black/black	opportunistic	campsite	surface
258	07/40	-	MCMP	25,9981	11,9771	hamada	desert pavement	42	20	10	2	no	19	2 = 10-26	black/black	shaped	campsite	building material
259	07/40	-	MCMP	25,9981	11,9771	hamada	desert pavement	50	25	14	2	no	40	3 = 26-50	black/black	shaped	campsite	building material
260	07/40	-	MCMP	25,9980	11,9770	hamada	desert pavement	59	27	13	1	no	48	3 = 26-50	black/black	opportunistic	campsite	surface
261	07/40	-	MCMP	25,9980	11,9771	hamada	desert pavement	74	35	14	2	no	83	4 = 50-85	black/black	opportunistic	campsite	building material
262	07/40	-	MCMP	25,9980	11,9771	hamada	desert pavement	46	13	6	2	no	8	1 =1-10	black/black	opportunistic	campsite	building material
263	07/40	-	MCMP	25,9980	11,9773	hamada	desert pavement	62	33	11	2	no	52	4 = 50-85	black/black	opportunistic	campsite	building material
264	07/40	-	MCMP	25,9980	11,9773	hamada	desert pavement	67	40	10	4	no	62	4 = 50-85	black/black	shaped	campsite	building material
265	07/40	-	MCMP	25,9980	11,9773	hamada	desert pavement	30	22	8	2	no	12	2 = 10-26	black/black	shaped	campsite	building material
266	07/40	-	MCMP	25,9980	11,9774	hamada	desert pavement	50	45	16	2	no	83	4 = 50-85	black/black	opportunistic	campsite	building material
267	07/40	-	MCMP	25,9980	11,9774	hamada	desert pavement	57	40	9	1	no	47	3 = 26-50	black/black	shaped	campsite	building material
268	07/40	-	MCMP	25,9980	11,9771	hamada	desert pavement	59	30	10	2	light	41	3 = 26-50	nd	opportunistic	campsite	building material
269	07/40	-	MCMP	25,9980	11,9774	hamada	desert pavement	38	27	7	2	no	17	2 = 10-26	black/black	opportunistic	campsite	building material
270	07/40	-	MCMP	25,9980	11,9771	hamada	desert pavement	58	37	18	3	no	89	5 = 85-146	black/black	opportunistic	campsite	building material
271	07/40	-	MCMP	25,9980	11,9771	hamada	desert pavement	58	43	5	3	no	29	3 = 26-50	black/black	opportunistic	campsite	surface
272	07/40	-	MCMP	25,9980	11,9772	hamada	desert pavement	55	41	8	2	no	41	3 = 26-50	black/black	opportunistic	campsite	building material
273	07/40	-	MCMP	25,9980	11,9772	hamada	desert pavement	43	25	10	2	no	25	2 = 10-26	black/black	opportunistic	campsite	building material
274	07/40	-	MCMP	25,9980	11,9772	hamada	desert pavement	48	34	10	2	no	38	3 = 26-50	black/black	opportunistic	campsite	building material
275	07/40	-	MCMP	25,9980	11,9773	hamada	desert pavement	72	36	16	2	circular	95	5 = 85-146	black/black	heavy grooved	campsite	surface
276	07/40	-	MCMP	25,9979	11,9774	hamada	desert pavement	61	15	5	2	no	11	2 = 10-26	black/black	shaped	campsite	building material
277	07/40	-	MCMP	25,9979	11,9767	hamada	desert pavement	55	30	11	1	no	42	3 = 26-50	black/black	shaped	campsite	building material
278	07/40	-	MCMP	25,9979	11,9774	hamada	desert pavement	57	43	10	2	no	56	4 = 50-85	black/black	shaped	campsite	surface
279	07/40	-	MCMP	25,9979	11,9771	hamada	desert pavement	60	40	15	1	no	83	4 = 50-85	black/black	opportunistic	campsite	surface
280	07/40	-	MCMP	25,9979	11,9773	hamada	desert pavement	48	19	7	2	no	15	2 = 10-26	black/black	opportunistic	campsite	surface
281	07/40	-	MCMP	25,9979	11,9769	hamada	desert pavement	63	43	13	4	circular	81	4 = 50-85	black/black	heavy grooved	campsite	surface
282	07/40	-	MCMP	25,9979	11,9767	hamada	desert pavement	60	29	12	2	no	48	3 = 26-50	black/black	opportunistic	campsite	building material
283	07/40	-	MCMP	25,9979	11,9773	hamada	desert pavement	47	30	6	2	no	19	2 = 10-26	black/black	shaped	campsite	building material
284	07/40	-	MCMP	25,9979	11,9775	hamada	desert pavement	49	28	12	2	no	38	3 = 26-50	black/black	opportunistic	campsite	building material
285	07/40	-	MCMP	25,9979	11,9773	hamada	desert pavement	71	40	10	2	no	65	4 = 50-85	black/black	shaped	campsite	building material
286	07/40	-	MCMP	25,9979	11,9773	hamada	desert pavement	45	21	11	2	no	24	2 = 10-26	black/black	opportunistic	campsite	building material
287	07/40	-	MCMP	25,9979	11,9773	hamada	desert pavement	60	30	11	3	no	46	3 = 26-50	black/black	opportunistic	campsite	building material
288	07/40	-	MCMP	25,9979	11,9775	hamada	desert pavement	48	35	5	2	no	19	2 = 10-26	black/black	shaped	campsite	building material
289	07/40	-	MCMP	25,9979	11,9767	hamada	desert pavement	45	42	8	2	no	35	3 = 26-50	black/black	opportunistic	campsite	building material
290	07/40	-	MCMP	25,9978	11,9775	hamada	desert pavement	50	29	7	2	no	23	2 = 10-26	black/black	opportunistic	campsite	building material
291	07/40	-	MCMP	25,9978	11,9770	hamada	desert pavement	44	35	13	2	no	46	3 = 26-50	black/black	opportunistic	campsite	surface
292	07/40	-	MCMP	25,9978	11,9775	hamada	desert pavement	51	31	6	2	no	22	2 = 10-26	black/black	opportunistic	campsite	building material
293	07/40	-	MCMP	25,9978	11,9775	hamada	desert pavement	40	25	8	2	no	18	2 = 10-26	black/black	shaped	campsite	building material
294	07/40	-	MCMP	25,9978	11,9775	hamada	desert pavement	64	0	14	2	no	0	nd	black/black	opportunistic	campsite	building material
295	07/40	-	MCMP	25,9978	11,9775	hamada	desert pavement	55	31	8	2	no	31	3 = 26-50	black/black	opportunistic	campsite	building material
296	07/40	-	MCMP	25,9978	11,9775	hamada	desert pavement	45	22	9	1	no	20	2 = 10-26	black/black	opportunistic	campsite	building material
297	07/40	-	MCMP	25,9978	11,9775	hamada	desert pavement	49	24	9	2	no	24	2 = 10-26	black/black	opportunistic	campsite	building material
298	07/40	-	MCMP	25,9978	11,9775	hamada	desert pavement	56	24	8	1	no	25	2 = 10-26	black/black	opportunistic	campsite	building material
299	07/40	-	MCMP	25,9978	11,9775	hamada	desert pavement	59	16	8	2	no	17	2 = 10-26	black/black	opportunistic	campsite	building material
300	07/40	-	MCMP	25,9978	11,9768	hamada	desert pavement	75	37	11	4	no	70	4 = 50-85	black/black	shaped	campsite	building material
301	07/40	-	MCMP	25,9978	11,9769	hamada	desert pavement	41	20	8	1	no	15	2 = 10-26	black/black	opportunistic	campsite	surface
302	07/40	-	MCMP	25,9977	11,9773	hamada	desert pavement	54	26	10	2	no	32	3 = 26-50	black/red	shaped	campsite	surface
303	07/40	-	MCMP	25,9977	11,9776	hamada	desert pavement	49	24	12	2	no	32	3 = 26-50	black/red	shaped	campsite	building material
304	07/40	-	MCMP	25,9977	11,9772	hamada	desert pavement	48	38	5	2	no	21	2 = 10-26	black/black	shaped	campsite	surface
305	07/40	-	MCMP	25,9977	11,9772	hamada	desert pavement	72	45	13	2	no	97	5 = 85-146	black/black	shaped	campsite	surface
306	07/40	-	MCMP	25,9977	11,9776	hamada	desert pavement	68	37	14	3	no	81	4 = 50-85	black/black	shaped	campsite	building material
307	07/40	-	MCMP	25,9977	11,9771	hamada	desert pavement	53	42	5	2	no	26	2 = 10-26	black/black	opportunistic	campsite	surface
308	07/40	-	MCMP	25,9977	11,9776	hamada	desert pavement	50	35	9	3	no	36	3 = 26-50	black/black	shaped	campsite	building material
309	07/40	-	MCMP	25,9977	11,9776	hamada	desert pavement	80	30	9	2	no	50	3 = 26-50	black/black	shaped	campsite	building material
310	09/45	-	MCMP	25,9685	11,9463	hamada	endorheic depression	50	30	5	2	no	17	2 = 10-26	black/red	shaped	campsite	surface
311	09/47	-	MCMP	25,9693	11,9488	hamada	endorheic depression	72	30	7	1	no	35	3 = 26-50	black/red	shaped	campsite	surface
312	09/47	-	MCMP	25,9692	11,9488	hamada	endorheic depression	65	40	15	1	no	90	5 = 85-146	black/black	shaped	campsite	surface

313	09/47	-	MCMP	25,9692	11,9488	hamada	endorheic depression	60	40	12	2	no	66	4 = 50-85	black/black	shaped	campsite	surface
314	09/51	-	MCMP	25,9753	11,9560	hamada	endorheic depression	40	25	18	2	no	41	3 = 26-50	black/red	shaped	isolated	isolated
315	09/51	-	MCMP	25,9752	11,9560	hamada	endorheic depression	65	35	15	2	no	78	4 = 50-85	black/red	shaped	isolated	isolated
316	09/52	-	MCMP	25,9754	11,9561	hamada	endorheic depression	60	0	8	2	no	0	nd	nd	shaped	isolated	isolated
317	07/68	-	MCMP	26,0008	11,9782	hamada edge	desert pavement	37	18	6	2	no	9	1 =1-10	black/black	shaped	ceremonial/funerary context	surface
318	07/68	-	MCMP	26,0008	11,9781	hamada edge	desert pavement	54	53	7	1	no	46	3 = 26-50	black/red	shaped	ceremonial/funerary context	surface
319	07/68	-	MCMP	26,0008	11,9781	hamada edge	desert pavement	60	24	11	1	no	36	3 = 26-50	black/red	opportunistic	ceremonial/funerary context	surface
320	07/68	-	MCMP	26,0007	11,9783	hamada edge	desert pavement	48	24	6	2	no	16	2 = 10-26	black/red	shaped	ceremonial/funerary context	surface
321	07/68	-	MCMP	26,0007	11,9782	hamada edge	desert pavement	48	32	12	2	no	42	3 = 26-50	black/black	opportunistic	ceremonial/funerary context	surface
322	07/68	-	MCMP	26,0007	11,9783	hamada edge	desert pavement	53	38	7	2	no	32	3 = 26-50	black/black	opportunistic	ceremonial/funerary context	surface
323	07/68	-	MCMP	26,0007	11,9781	hamada edge	desert pavement	55	34	7	2	no	30	3 = 26-50	black/black	shaped	ceremonial/funerary context	surface
324	07/68	-	MCMP	26,0005	11,9784	hamada edge	desert pavement	36	37	8	2	no	25	2 = 10-26	black/black	opportunistic	ceremonial/funerary context	surface
325	07/68	-	MCMP	26,0005	11,9785	hamada edge	desert pavement	41	21	7	2	no	14	2 = 10-26	black/red	opportunistic	ceremonial/funerary context	surface
326	07/68	-	MCMP	26,0005	11,9784	hamada edge	desert pavement	53	30	4	2	no	15	2 = 10-26	black/red	shaped	ceremonial/funerary context	surface
327	07/68	-	MCMP	26,0005	11,9783	hamada edge	desert pavement	48	29	10	2	no	32	3 = 26-50	black/black	shaped	ceremonial/funerary context	surface
328	07/68	-	MCMP	26,0004	11,9780	hamada edge	desert pavement	62	28	6	1	no	24	2 = 10-26	black/black	shaped	ceremonial/funerary context	surface
329	07/68	-	MCMP	26,0004	11,9785	hamada edge	desert pavement	67	44	8	2	light	54	4 = 50-85	black/black	shaped	ceremonial/funerary context	surface
330	07/68	-	MCMP	26,0004	11,9778	hamada edge	desert pavement	34	17	9	2	no	12	2 = 10-26	black/black	shaped	ceremonial/funerary context	surface
331	07/68	-	MCMP	26,0004	11,9777	hamada edge	desert pavement	32	18	6	2	no	8	1 =1-10	black/red	shaped	ceremonial/funerary context	surface
332	07/68	-	MCMP	26,0004	11,9774	hamada edge	desert pavement	57	29	13	1	light	49	3 = 26-50	black/red	shaped	ceremonial/funerary context	surface
333	07/68	-	MCMP	26,0003	11,9778	hamada edge	desert pavement	42	26	5	1	no	13	2 = 10-26	black/red	shaped	ceremonial/funerary context	surface
334	07/68	-	MCMP	26,0003	11,9784	hamada edge	desert pavement	39	15	6	2	no	8	1 =1-10	black/black	opportunistic	ceremonial/funerary context	surface
335	07/68	-	MCMP	26,0003	11,9781	hamada edge	desert pavement	36	21	14	1	no	24	2 = 10-26	black/red	opportunistic	ceremonial/funerary context	surface
336	07/68	-	MCMP	26,0003	11,9785	hamada edge	desert pavement	45	24	5	1	no	12	2 = 10-26	black/black	shaped	ceremonial/funerary context	surface
337	07/68	-	MCMP	26,0003	11,9783	hamada edge	desert pavement	61	20	7	2	no	20	2 = 10-26	black/red	opportunistic	ceremonial/funerary context	surface
338	07/68	-	MCMP	26,0003	11,9785	hamada edge	desert pavement	54	26	18	2	no	58	4 = 50-85	black/red	opportunistic	ceremonial/funerary context	surface
339	07/68	-	MCMP	26,0003	11,9780	hamada edge	desert pavement	89	25	12	2	no	61	4 = 50-85	black/red	opportunistic	ceremonial/funerary context	surface
340	07/68	-	MCMP	26,0003	11,9785	hamada edge	desert pavement	37	39	7	1	no	23	2 = 10-26	black/red	opportunistic	ceremonial/funerary context	surface
341	07/68	-	MCMP	26,0002	11,9777	hamada edge	desert pavement	56	40	10	1	no	52	4 = 50-85	black/red	opportunistic	ceremonial/funerary context	surface
342	07/68	-	MCMP	26,0002	11,9786	hamada edge	desert pavement	64	34	28	2	no	140	5 = 85-146	black/red	shaped	ceremonial/funerary context	surface
343	07/68	-	MCMP	26,0001	11,9778	hamada edge	desert pavement	33	34	5	2	no	13	2 = 10-26	black/red	opportunistic	ceremonial/funerary context	surface
344	07/68	-	MCMP	26,0001	11,9782	hamada edge	desert pavement	35	25	9	2	no	18	2 = 10-26	black/red	shaped	ceremonial/funerary context	surface
345	07/68	-	MCMP	26,0001	11,9778	hamada edge	desert pavement	72	46	9	1	no	69	4 = 50-85	black/red	shaped	ceremonial/funerary context	surface
346	07/68	-	MCMP	26,0001	11,9779	hamada edge	desert pavement	73	27	10	2	no	45	3 = 26-50	black/red	shaped	ceremonial/funerary context	surface
347	07/68	-	MCMP	26,0001	11,9780	hamada edge	desert pavement	43	21	11	1	no	23	2 = 10-26	black/black	shaped	ceremonial/funerary context	surface
348	07/68	-	MCMP	26,0001	11,9781	hamada edge	desert pavement	83	20	7	2	no	27	3 = 26-50	black/black	shaped	ceremonial/funerary context	surface
349	07/68	-	MCMP	26,0001	11,9778	hamada edge	desert pavement	36	24	11	1	no	22	2 = 10-26	black/red	shaped	ceremonial/funerary context	surface
350	07/68	-	MCMP	26,0001	11,9783	hamada edge	desert pavement	90	46	13	2	light	124	5 = 85-146	black/black	shaped	ceremonial/funerary context	surface
351	07/68	-	MCMP	26,0001	11,9783	hamada edge	desert pavement	64	32	9	2	no	42	3 = 26-50	black/red	shaped	ceremonial/funerary context	surface
352	07/68	-	MCMP	26,0001	11,9783	hamada edge	desert pavement	66	34	28	2	no	145	5 = 85-146	black/red	shaped	ceremonial/funerary context	surface
353	07/68	-	MCMP	26,0001	11,9777	hamada edge	desert pavement	58	34	19	1	no	86	5 = 85-146	black/black	shaped	ceremonial/funerary context	surface
354	07/68	-	MCMP	26,0001	11,9787	hamada edge	desert pavement	69	47	28	2	no	209	6 = 146-295	black/black	shaped	ceremonial/funerary context	surface
355	07/68	-	MCMP	26,0001	11,9781	hamada edge	desert pavement	40	20	8	2	no	15	2 = 10-26	black/black	shaped	ceremonial/funerary context	surface
356	07/68	-	MCMP	26,0000	11,9783	hamada edge	desert pavement	55	48	13	1	no	79	4 = 50-85	black/red	opportunistic	ceremonial/funerary context	surface
357	07/68	-	MCMP	26,0000	11,9784	hamada edge	desert pavement	77	42	25	2	no	186	6 = 146-295	black/red	opportunistic	ceremonial/funerary context	surface
358	07/68	-	MCMP	26,0000	11,9784	hamada edge	desert pavement	62	38	13	3	no	70	4 = 50-85	black/red	opportunistic	ceremonial/funerary context	surface
359	07/68	-	MCMP	26,0000	11,9784	hamada edge	desert pavement	67	55	21	1	no	178	6 = 146-295	black/black	opportunistic	ceremonial/funerary context	surface
360	07/68	-	MCMP	26,0000	11,9782	hamada edge	desert pavement	78	47	10	2	no	84	4 = 50-85	black/black	opportunistic	ceremonial/funerary context	surface
361	07/68	-	MCMP	25,9999	11,9788	hamada edge	desert pavement	74	37	13	2	no	82	4 = 50-85	black/black	opportunistic	ceremonial/funerary context	surface
362	07/68	-	MCMP	25,9999	11,9782	hamada edge	desert pavement	62	29	20	2	no	83	4 = 50-85	black/red	opportunistic	ceremonial/funerary context	surface
363	07/68	-	MCMP	25,9999	11,9787	hamada edge	desert pavement	87	52	17	2	no	177	6 = 146-295	black/red	shaped	ceremonial/funerary context	surface
364	07/68	-	MCMP	25,9999	11,9786	hamada edge	desert pavement	55	33	19	2	no	79	4 = 50-85	black/black	opportunistic	ceremonial/funerary context	surface
365	07/68	-	MCMP	25,9999	11,9786	hamada edge	desert pavement	70	26	17	1	no	71	4 = 50-85	black/black	nd	ceremonial/funerary context	surface
366	07/68	-	MCMP	25,9999	11,9786	hamada edge	desert pavement	41	30	6	2	no	17	2 = 10-26	black/red	shaped	ceremonial/funerary context	surface
367	07/68	-	MCMP	25,9999	11,9788	hamada edge	desert pavement	74	30	11	2	no	56	4 = 50-85	black/red	opportunistic	ceremonial/funerary context	surface
368	07/68	-	MCMP	25,9999	11,9788	hamada edge	desert pavement	76	54	12	2	no	113	5 = 85-146	black/black	shaped	ceremonial/funerary context	surface
369	07/68	-	MCMP	25,9999	11,9779	hamada edge	desert pavement	59	32	9	2	no	39	3 = 26-50	black/black	shaped	ceremonial/funerary context	surface
370	07/68	-	MCMP	25,9999	11,9783	hamada edge	desert pavement	49	30	18	2	no	61	4 = 50-85	black/red	opportunistic	ceremonial/funerary context	surface
371	07/68	-	MCMP	25,9999	11,9782	hamada edge	desert pavement	60	41	14	2	no	79	4 = 50-85	black/red	shaped	ceremonial/funerary context	surface
372	07/68	-	MCMP	25,9999	11,9788	hamada edge	desert pavement	65	27	23	2	light	93	5 = 85-146	black/black	shaped	ceremonial/funerary context	surface
373	07/68	-	MCMP	25,9998	11,9787	hamada edge	desert pavement	62	42	22	2	light	132	5 = 85-146	black/black	shaped	ceremonial/funerary context	surface
374	07/68	-	MCMP	25,9998	11,9787	hamada edge	desert pavement	62	39	23	2	no	128	5 = 85-146	black/red	opportunistic	ceremonial/funerary context	surface
375	07/68	-	MCMP	25,9998	11,9788	hamada edge	desert pavement	59	28	14	2	no	53	4 = 50-85	black/black	opportunistic	ceremonial/funerary context	surface

376	07/68	-	MCMP	25,9998	11,9788	hamada edge	desert pavement	42	47	17	2	no	77	4 = 50-85	black/black	shaped	ceremonial/funerary context	surface
377	07/68	-	MCMP	25,9998	11,9788	hamada edge	desert pavement	62	40	16	2	no	91	5 = 85-146	black/red	opportunistic	ceremonial/funerary context	surface
378	07/68	-	MCMP	25,9998	11,9788	hamada edge	desert pavement	46	28	9	1	no	27	3 = 26-50	black/red	opportunistic	ceremonial/funerary context	surface
379	07/68	-	MCMP	25,9998	11,9788	hamada edge	desert pavement	44	24	9	2	no	22	2 = 10-26	black/black	shaped	ceremonial/funerary context	surface
380	07/68	-	MCMP	25,9998	11,9784	hamada edge	desert pavement	53	46	24	2	no	135	5 = 85-146	black/black	opportunistic	ceremonial/funerary context	surface
381	07/68	-	MCMP	25,9998	11,9785	hamada edge	desert pavement	59	31	25	2	no	105	5 = 85-146	black/red	shaped	ceremonial/funerary context	surface
382	07/68	-	MCMP	25,9998	11,9789	hamada edge	desert pavement	45	39	18	2	no	73	4 = 50-85	black/red	shaped	ceremonial/funerary context	surface
383	07/68	-	MCMP	25,9998	11,9785	hamada edge	desert pavement	48	38	10	2	light	42	3 = 26-50	black/red	shaped	ceremonial/funerary context	surface
384	07/68	-	MCMP	25,9998	11,9784	hamada edge	desert pavement	60	39	10	2	no	54	4 = 50-85	black/black	shaped	ceremonial/funerary context	surface
385	07/68	-	MCMP	25,9998	11,9788	hamada edge	desert pavement	54	48	18	2	no	107	5 = 85-146	black/red	shaped	ceremonial/funerary context	surface
386	07/68	-	MCMP	25,9998	11,9784	hamada edge	desert pavement	55	37	15	2	no	70	4 = 50-85	black/red	shaped	ceremonial/funerary context	surface
387	07/68	-	MCMP	25,9998	11,9786	hamada edge	desert pavement	75	39	24	1	no	161	6 = 146-295	black/black	opportunistic	ceremonial/funerary context	surface
388	07/68	-	MCMP	25,9998	11,9788	hamada edge	desert pavement	76	42	18	2	no	132	5 = 85-146	black/red	shaped	ceremonial/funerary context	surface
389	07/68	-	MCMP	25,9998	11,9784	hamada edge	desert pavement	47	22	15	2	no	36	3 = 26-50	black/red	opportunistic	ceremonial/funerary context	surface
390	07/68	-	MCMP	25,9998	11,9789	hamada edge	desert pavement	79	37	6	2	no	40	3 = 26-50	black/red	opportunistic	ceremonial/funerary context	surface
391	07/68	-	MCMP	25,9998	11,9784	hamada edge	desert pavement	47	24	22	1	no	53	4 = 50-85	black/red	opportunistic	ceremonial/funerary context	surface
392	07/68	-	MCMP	25,9997	11,9783	hamada edge	desert pavement	48	29	20	1	no	64	4 = 50-85	black/black	opportunistic	ceremonial/funerary context	surface
393	07/68	-	MCMP	25,9997	11,9788	hamada edge	desert pavement	48	48	6	2	no	32	3 = 26-50	black/red	opportunistic	ceremonial/funerary context	surface
394	07/68	-	MCMP	25,9997	11,9782	hamada edge	desert pavement	74	39	9	1	no	60	4 = 50-85	black/red	opportunistic	ceremonial/funerary context	surface
395	07/68	-	MCMP	25,9997	11,9788	hamada edge	desert pavement	81	37	19	1	no	131	5 = 85-146	black/red	opportunistic	ceremonial/funerary context	surface
396	07/68	-	MCMP	25,9997	11,9787	hamada edge	desert pavement	72	36	13	2	no	78	4 = 50-85	black/black	shaped	ceremonial/funerary context	surface
397	07/68	-	MCMP	25,9997	11,9788	hamada edge	desert pavement	60	27	13	1	no	48	3 = 26-50	black/red	opportunistic	ceremonial/funerary context	surface
398	07/68	-	MCMP	25,9997	11,9788	hamada edge	desert pavement	50	36	13	2	no	54	4 = 50-85	black/red	shaped	ceremonial/funerary context	surface
399	07/68	-	MCMP	25,9997	11,9786	hamada edge	desert pavement	60	53	22	2	no	161	6 = 146-295	black/red	shaped	ceremonial/funerary context	surface
400	07/68	-	MCMP	25,9997	11,9786	hamada edge	desert pavement	58	36	6	2	no	29	3 = 26-50	black/red	shaped	ceremonial/funerary context	surface
401	07/68	-	MCMP	25,9997	11,9785	hamada edge	desert pavement	47	43	10	1	no	46	3 = 26-50	black/red	opportunistic	ceremonial/funerary context	surface
402	07/68	-	MCMP	25,9997	11,9786	hamada edge	desert pavement	70	45	31	1	no	225	6 = 146-295	black/red	shaped	ceremonial/funerary context	surface
403	07/68	-	MCMP	25,9997	11,9787	hamada edge	desert pavement	35	18	7	1	no	10	2 = 10-26	black/red	opportunistic	ceremonial/funerary context	surface
404	07/68	-	MCMP	25,9997	11,9784	hamada edge	desert pavement	63	55	9	2	no	72	4 = 50-85	black/red	shaped	ceremonial/funerary context	surface
405	07/68	-	MCMP	25,9997	11,9786	hamada edge	desert pavement	63	34	26	2	no	128	5 = 85-146	black/red	shaped	ceremonial/funerary context	surface
406	07/68	-	MCMP	25,9997	11,9784	hamada edge	desert pavement	54	34	18	1	no	76	4 = 50-85	black/red	opportunistic	ceremonial/funerary context	surface
407	07/68	-	MCMP	25,9997	11,9786	hamada edge	desert pavement	72	30	18	2	no	89	5 = 85-146	black/black	shaped	ceremonial/funerary context	surface
408	07/68	-	MCMP	25,9997	11,9783	hamada edge	desert pavement	66	36	19	1	no	104	5 = 85-146	black/black	opportunistic	ceremonial/funerary context	surface
409	07/68	-	MCMP	25,9997	11,9788	hamada edge	desert pavement	70	35	12	2	no	68	4 = 50-85	black/black	opportunistic	ceremonial/funerary context	surface
410	07/68	-	MCMP	25,9997	11,9788	hamada edge	desert pavement	57	38	13	2	no	65	4 = 50-85	black/red	shaped	ceremonial/funerary context	surface
411	07/68	-	MCMP	25,9997	11,9787	hamada edge	desert pavement	39	22	6	2	no	12	2 = 10-26	black/red	shaped	ceremonial/funerary context	surface
412	07/68	-	MCMP	25,9997	11,9787	hamada edge	desert pavement	52	33	8	2	no	32	3 = 26-50	black/black	opportunistic	ceremonial/funerary context	surface
413	07/68	-	MCMP	25,9997	11,9784	hamada edge	desert pavement	52	41	10	2	no	49	3 = 26-50	black/red	shaped	ceremonial/funerary context	surface
414	07/68	-	MCMP	25,9997	11,9789	hamada edge	desert pavement	48	30	5	1	no	17	2 = 10-26	black/black	opportunistic	ceremonial/funerary context	surface
415	07/68	-	MCMP	25,9996	11,9783	hamada edge	slope	51	33	7	1	no	27	3 = 26-50	black/red	opportunistic	ceremonial/funerary context	surface
416	07/68	-	MCMP	25,9996	11,9788	hamada edge	desert pavement	63	41	5	2	no	30	3 = 26-50	black/red	shaped	ceremonial/funerary context	surface
417	07/68	-	MCMP	25,9996	11,9783	hamada edge	slope	43	21	12	2	no	25	2 = 10-26	black/red	opportunistic	ceremonial/funerary context	surface
418	07/68	-	MCMP	25,9996	11,9787	hamada edge	slope	47	35	12	1	no	45	3 = 26-50	black/red	opportunistic	ceremonial/funerary context	surface
419	07/68	-	MCMP	25,9996	11,9786	hamada edge	slope	79	37	11	1	light	74	4 = 50-85	black/red	shaped	ceremonial/funerary context	surface
420	07/68	-	MCMP	25,9996	11,9785	hamada edge	slope	58	46	12	2	no	74	4 = 50-85	black/black	opportunistic	ceremonial/funerary context	surface
421	07/68	-	MCMP	25,9996	11,9785	hamada edge	slope	72	36	12	1	no	72	4 = 50-85	black/red	shaped	ceremonial/funerary context	surface
422	07/68	-	MCMP	25,9996	11,9785	hamada edge	slope	44	25	12	2	no	30	3 = 26-50	black/red	opportunistic	ceremonial/funerary context	surface
423	07/68	-	MCMP	25,9996	11,9786	hamada edge	slope	95	42	9	1	no	83	4 = 50-85	black/red	opportunistic	ceremonial/funerary context	surface
424	07/68	-	MCMP	25,9995	11,9785	hamada edge	slope	89	57	18	2	no	210	6 = 146-295	black/black	shaped	ceremonial/funerary context	surface
425	07/68	-	MCMP	25,9995	11,9784	hamada edge	slope	73	36	9	1	no	54	4 = 50-85	black/black	opportunistic	ceremonial/funerary context	surface
426	07/68	-	MCMP	25,9995	11,9785	hamada edge	slope	52	39	19	1	no	89	5 = 85-146	black/red	shaped	ceremonial/funerary context	surface
427	07/68	-	MCMP	25,9995	11,9786	hamada edge	slope	33	15	5	1	no	6	1 =1-10	black/black	opportunistic	ceremonial/funerary context	surface
428	07/68	-	MCMP	25,9995	11,9793	hamada edge	slope	33	17	5	1	no	6	1 =1-10	black/black	shaped	ceremonial/funerary context	surface
429	07/68	-	MCMP	25,9994	11,9786	hamada edge	slope	50	33	8	1	no	30	3 = 26-50	black/red	opportunistic	ceremonial/funerary context	surface
430	07/68	-	MCMP	25,9994	11,9786	hamada edge	slope	41	30	7	1	no	20	2 = 10-26	black/black	opportunistic	ceremonial/funerary context	surface
431	07/68	-	MCMP	25,9994	11,9790	hamada edge	slope	39	29	10	1	no	26	2 = 10-26	black/black	shaped	ceremonial/funerary context	surface
432	07/68	-	MCMP	25,9993	11,9790	hamada edge	slope	50	22	12	1	no	30	3 = 26-50	black/black	opportunistic	ceremonial/funerary context	surface
433	07/68	-	MCMP	25,9992	11,9795	hamada edge	slope	26	11	5	2	no	3	1 =1-10	black/black	shaped	ceremonial/funerary context	surface
434	07/68	-	MCMP	25,9991	11,9795	hamada edge	slope	34	14	3	2	no	3	1 =1-10	black/red	opportunistic	ceremonial/funerary context	surface
435	09/69	-	MCMP	26,0069	12,0475	hamada	slope	50	33	10	2	no	38	3 = 26-50	nd	shaped	ceremonial/funerary context	surface
436	09/69	-	MCMP	26,0069	12,0475	hamada	slope	43	24	8	1	no	19	2 = 10-26	nd	opportunistic	ceremonial/funerary context	surface
437	09/69	-	MCMP	26,0067	12,0476	hamada	slope	44	30	8	2	no	24	2 = 10-26	nd	nd	ceremonial/funerary context	surface
438	09/69	-	MCMP	26,0066	12,0476	hamada	slope	80	50	32	2	circular	294	6 = 146-295	black/black	heavy grooved	ceremonial/funerary context	surface

439	09/73	-	MCMP	26,0063	11,9907	hamada	desert pavement	40	30	10	#NULL!	no	28	3 = 26-50	black/black	nd	campsite	building material
440	09/82	-	MCMP	25,9877	11,9850	hamada	endorheic depression	40	25	20	2	no	46	3 = 26-50	black/red	shaped	isolated	isolated
441	09/16	-	MCMP	25,8363	12,1860	hamada	desert pavement	45	28	15	2	no	43	3 = 26-50	black/red	shaped	campsite	surface
442	09/16	-	MCMP	25,8363	12,1861	hamada	desert pavement	35	20	15	2	no	24	2 = 10-26	black/black	shaped	campsite	surface
443	09/16	-	MCMP	25,8362	12,1861	hamada	desert pavement	50	25	10	1	no	29	3 = 26-50	black/black	opportunistic	campsite	surface
444	09/16	-	MCMP	25,8362	12,1861	hamada	desert pavement	45	30	20	2	no	62	4 = 50-85	black/black	nd	campsite	surface
445	09/16	-	MCMP	25,8362	12,1862	hamada	desert pavement	55	25	20	2	no	63	4 = 50-85	black/black	shaped	campsite	surface
446	09/16	-	MCMP	25,8361	12,1861	hamada	desert pavement	50	30	20	2	no	69	4 = 50-85	black/red	nd	campsite	surface
447	09/16	-	MCMP	25,8361	12,1861	hamada	desert pavement	40	25	15	2	no	35	3 = 26-50	black/black	shaped	campsite	surface
448	09/16	-	MCMP	25,8361	12,1861	hamada	desert pavement	40	30	12	1	no	33	3 = 26-50	black/black	shaped	campsite	surface
449	09/16	-	MCMP	25,8361	12,1860	hamada	desert pavement	40	20	15	2	no	28	3 = 26-50	black/red	nd	campsite	surface
450	09/16	-	MCMP	25,8361	12,1862	hamada	desert pavement	45	25	15	2	no	39	3 = 26-50	black/red	nd	campsite	surface
451	09/16	-	MCMP	25,8361	12,1862	hamada	desert pavement	55	25	25	1	no	79	4 = 50-85	black/red	shaped	campsite	surface
452	09/16	-	MCMP	25,8360	12,1862	hamada	desert pavement	65	30	10	2	no	45	3 = 26-50	black/red	nd	campsite	surface
453	09/16	-	MCMP	25,8359	12,1862	hamada	desert pavement	60	30	17	2	no	70	4 = 50-85	black/red	shaped	campsite	surface
454	09/16	-	MCMP	25,8359	12,1863	hamada	desert pavement	60	30	17	2	no	70	4 = 50-85	black/red	shaped	campsite	surface
455	09/16	-	MCMP	25,8359	12,1863	hamada	desert pavement	45	25	24	2	no	62	4 = 50-85	black/black	shaped	campsite	surface
456	09/17	-	MCMP	25,8359	12,1920	hamada	desert pavement	30	20	12	2	no	17	2 = 10-26	black/red	shaped	campsite	surface
457	09/17	-	MCMP	25,8357	12,1918	hamada	desert pavement	50	25	15	2	no	43	3 = 26-50	black/red	shaped	campsite	surface
458	09/17	-	MCMP	25,8356	12,1917	hamada	desert pavement	40	25	15	2	no	35	3 = 26-50	black/red	shaped	campsite	surface
459	09/17	-	MCMP	25,8356	12,1919	hamada	desert pavement	70	25	25	2	no	101	5 = 85-146	black/red	nd	campsite	surface
460	09/17	-	MCMP	25,8356	12,1920	hamada	desert pavement	60	30	20	2	no	83	4 = 50-85	black/red	shaped	campsite	surface
461	09/17	-	MCMP	25,8355	12,1920	hamada	desert pavement	55	28	14	2	no	50	3 = 26-50	black/red	shaped	campsite	surface
462	09/17	-	MCMP	25,8352	12,1916	hamada	desert pavement	70	35	20	2	no	113	5 = 85-146	black/red	shaped	campsite	surface
463	09/18	-	MCMP	25,8345	12,1977	hamada	endorheic depression	100	45	28	2	circular	290	6 = 146-295	black/red	heavy grooved	campsite	surface
464	09/20	-	MCMP	25,8365	12,2008	hamada	desert pavement	40	25	15	2	no	35	3 = 26-50	nd	nd	campsite	surface
465	09/23	-	MCMP	25,9807	11,9592	hamada	desert pavement	75	25	20	2	circular	86	5 = 85-146	nd	heavy grooved	isolated	isolated
466	09/25	-	MCMP	25,9807	11,9592	hamada	desert pavement	65	45	20	2	no	135	5 = 85-146	black/black	shaped	isolated	isolated
467	09/28	-	MCMP	25,9791	11,9575	hamada	desert pavement	47	20	17	2	no	37	3 = 26-50	black/red	shaped	isolated	isolated
468	MP10/111	-	MP	24,3089	11,4919	hamada	desert pavement	50	26	10	2	no	30	3 = 26-50	black/red	shaped	other	surface
469	MP10/114	-	MP	24,3089	11,4921	hamada	slope	0	0	0	2	no	0	nd	nd	nd	other	surface
470	MP10/114	-	MP	24,3089	11,4921	hamada	slope	0	0	0	2	no	0	nd	nd	nd	other	surface
471	MP10/114	-	MP	24,3089	11,4921	hamada	slope	0	0	0	2	no	0	nd	nd	nd	other	surface
472	MP10/114	-	MP	24,3089	11,4921	hamada	slope	0	0	0	2	no	0	nd	nd	nd	other	surface
473	MP10/114	-	MP	24,3089	11,4921	hamada	slope	0	0	0	2	no	0	nd	nd	nd	other	surface
474	MP10/117	-	MP	24,3014	11,5064	hamada	desert pavement	100	25	10	2	no	58	4 = 50-85	black/black	shaped	ceremonial/funerary context	surface
475	MP10/117	-	MP	24,3014	11,5064	hamada	desert pavement	52	20	8	2	no	19	2 = 10-26	black/black	shaped	ceremonial/funerary context	surface
476	MP10/166	3	MP	24,4835	11,4715	hamada	slope	45	28	16	2	circular	46	3 = 26-50	black/black	heavy grooved	isolated	isolated
477	MP10/177	3	MP	24,4766	11,4489	hamada	slope	50	40	15	2	light	69	4 = 50-85	black/black	opportunistic	campsite	surface
478	MP10/183	3	MP	24,4814	11,4735	hamada	slope	87	50	20	2	circular	200	6 = 146-295	nd	heavy grooved	campsite	surface
479	MP10/242	-	MP	24,4986	11,6479	hamada	endorheic depression	60	50	15	1	no	104	5 = 85-146	black/black	shaped	isolated	isolated
480	MP10/244-b	-	MP	24,4469	11,5832	hamada	slope	0	0	0	2	circular	0	nd	nd	heavy grooved	ceremonial/funerary structure (n	building material
481	MP10/247	-	MP	24,4823	11,5473	hamada	slope	40	20	20	2	circular	37	3 = 26-50	black/black	heavy grooved	isolated	isolated
482	MP10/327	-	MP	24,5651	11,6078	hamada	desert pavement	50	21	14	2	circular	34	3 = 26-50	black/red	heavy grooved	isolated	isolated
483	MP10/376	4	MP	24,5567	11,5813	hamada	slope	50	30	15	2	no	52	4 = 50-85	nd	nd	other	surface
484	MP10/555	8	MP	24,9654	11,7367	hamada	endorheic depression	55	25	20	2	circular	63	4 = 50-85	black/red	heavy grooved	campsite	surface
485	MP10/555	8	MP	24,9654	11,7367	hamada	endorheic depression	40	40	7	2	no	26	2 = 10-26	black/red	shaped	campsite	surface
486	MP10/660	10	MP	25,1142	11,6340	hamada	endorheic depression	33	21	10	2	circular	16	2 = 10-26	nd	heavy grooved	campsite	surface
487	MP10/666	10	MP	25,1142	11,6562	hamada	endorheic depression	45	32	10	2	no	33	3 = 26-50	nd	shaped	campsite	surface
488	MP10/675	10	MP	25,1137	11,6464	hamada	endorheic depression	30	22	15	2	light	23	2 = 10-26	black/black	shaped	campsite	surface
489	MP10/675	10	MP	25,1137	11,6464	hamada	endorheic depression	45	25	20	2	no	52	4 = 50-85	black/black	shaped	campsite	surface
490	MP10/699	10	MP	25,1240	11,6429	hamada	endorheic depression	35	26	8	2	no	17	2 = 10-26	nd	shaped	campsite	surface
491	MP10/699	10	MP	25,1240	11,6429	hamada	endorheic depression	37	21	10	2	no	18	2 = 10-26	nd	shaped	campsite	surface
492	MP10/712	10	MP	25,1192	11,6494	hamada	endorheic depression	30	20	13	2	circular	18	2 = 10-26	black/red	heavy grooved	campsite	surface
493	MP10/714	10	MP	25,1159	11,6455	hamada	endorheic depression	30	30	17	2	no	35	3 = 26-50	black/black	shaped	campsite	surface
494	MP11/005	42	MP	26,2016	12,5360	hamada	endorheic depression	54	35	13	2	circular	57	4 = 50-85	black/red	heavy grooved	isolated	isolated
495	MP11/010	42	MP	26,2086	12,5470	hamada	endorheic depression	43	25	12	2	no	30	3 = 26-50	nd	shaped	isolated	isolated
496	MP11/065	43	MP	26,0872	12,5708	hamada	endorheic depression	80	37	0	2	no	0	nd	nd	shaped	campsite	surface
497	MP11/067	43	MP	26,0876	12,5678	hamada	endorheic depression	80	30	10	2	light	55	4 = 50-85	nd	shaped	campsite	surface
498	MP11/067	43	MP	26,0876	12,5678	hamada	endorheic depression	40	17	10	2	no	16	2 = 10-26	nd	shaped	campsite	surface
499	MP11/1031	19	MP	25,5586	11,8236	hamada	desert pavement	37	22	8	2	no	15	2 = 10-26	black/red	opportunistic	isolated	isolated
500	MP11/1032	19	MP	25,5583	11,8312	hamada	desert pavement	40	27	17	2	circular	42	3 = 26-50	black/red	heavy grooved	isolated	isolated
501	MP11/1035	19	MP	25,5628	11,8400	hamada	desert pavement	40	23	10	2	no	21	2 = 10-26	black/black	shaped	isolated	isolated

502	MP11/1037	19	MP	25,5633	11,8123	hamada	endorheic depression	22	13	15	2	light	10	1 =1-10	black/black	shaped	campsite	surface
503	MP11/1037	19	MP	25,5633	11,8123	hamada	endorheic depression	30	15	13	2	circular	13	2 = 10-26	black/black	heavy grooved	campsite	surface
504	MP11/1037	19	MP	25,5633	11,8123	hamada	endorheic depression	25	12	10	2	no	7	1 =1-10	black/black	opportunistic	campsite	surface
505	MP11/1068	20	MP	25,7056	11,6406	hamada	desert pavement	47	23	10	2	circular	25	2 = 10-26	black/red	heavy grooved	isolated	isolated
506	MP11/1070	20	MP	25,7053	11,6440	hamada	desert pavement	37	31	12	2	circular	32	3 = 26-50	black/black	heavy grooved	isolated	isolated
507	MP11/1089	16	MP	25,4965	11,6403	hamada	desert pavement	57	32	8	2	circular	34	3 = 26-50	nd	heavy grooved	isolated	isolated
508	MP11/1093	16	MP	25,5035	11,6405	hamada	desert pavement	30	15	10	2	circular	10	2 = 10-26	black/black	heavy grooved	isolated	isolated
509	MP11/1095	16	MP	25,5080	11,6544	hamada	endorheic depression	33	21	8	2	light	13	2 = 10-26	black/red	shaped	other	surface
510	MP11/1095	16	MP	25,5080	11,6544	hamada	endorheic depression	70	20	5	2	no	16	2 = 10-26	black/red	shaped	other	surface
511	MP11/1103	16	MP	25,4979	11,6681	hamada	endorheic depression	40	40	10	2	no	37	3 = 26-50	black/black	shaped	campsite	surface
512	MP11/1106	16	MP	25,5026	11,6588	hamada	desert pavement	25	17	12	2	no	12	2 = 10-26	black/red	shaped	isolated	isolated
513	MP11/1107	16	MP	25,4974	11,6466	hamada	endorheic depression	63	40	22	2	circular	128	5 = 85-146	black/red	heavy grooved	isolated	isolated
514	MP11/1109	16	MP	25,4974	11,6563	hamada	desert pavement	40	25	10	2	circular	23	2 = 10-26	nd	heavy grooved	ceremonial/funerary structure (n	building material
515	MP11/114	34	MP	26,1962	11,9366	hamada	endorheic depression	57	30	10	2	no	39	3 = 26-50	nd	shaped	isolated	isolated
516	MP11/1143	18	MP	25,6130	11,6854	hamada	endorheic depression	33	18	14	2	light	19	2 = 10-26	black/red	shaped	campsite	surface
517	MP11/1148	18	MP	25,6184	11,6608	hamada	endorheic depression	26	20	8	2	no	7	1 =1-10	black/red	opportunistic	campsite	surface
518	MP11/115	34	MP	26,1965	11,9328	hamada	endorheic depression	28	15	8	2	circular	8	1 =1-10	black/red	heavy grooved	campsite	surface
519	MP11/115	34	MP	26,1965	11,9328	hamada	endorheic depression	46	26	10	2	circular	28	3 = 26-50	black/red	heavy grooved	campsite	surface
520	MP11/1176	18	MP	25,6167	11,6575	hamada	desert pavement	37	24	9	2	circular	18	2 = 10-26	black/red	heavy grooved	isolated	isolated
521	MP11/1202	25	MP	25,8087	11,9534	hamada	desert pavement	43	0	12	2	circular	0	nd	nd	heavy grooved	isolated	isolated
522	MP11/1202	25	MP	25,8087	11,9534	hamada	desert pavement	48	0	6	2	circular	0	nd	nd	heavy grooved	isolated	isolated
523	MP11/1253	26	MP	25,7536	12,0382	hamada	endorheic depression	42	29	8	2	light	22	2 = 10-26	black/red	shaped	isolated	isolated
524	MP11/1307	23	MP	25,7155	11,8993	hamada	desert pavement	37	20	12	2	no	20	2 = 10-26	black/red	shaped	isolated	isolated
525	MP11/1328	21	MP	25,6030	11,9600	hamada	endorheic depression	0	0	0	2	no	0	nd	nd	nd	campsite	surface
526	MP11/1328	21	MP	25,6030	11,9600	hamada	endorheic depression	0	0	0	2	no	0	nd	nd	nd	campsite	surface
527	MP11/1328	21	MP	25,6030	11,9600	hamada	endorheic depression	0	0	0	2	no	0	nd	nd	nd	campsite	surface
528	MP11/1328	21	MP	25,6030	11,9600	hamada	endorheic depression	0	0	0	2	no	0	nd	nd	nd	campsite	surface
529	MP11/1328	21	MP	25,6030	11,9600	hamada	endorheic depression	0	0	0	2	no	0	nd	nd	nd	campsite	surface
530	MP11/1328	21	MP	25,6030	11,9600	hamada	endorheic depression	0	0	0	2	no	0	nd	nd	nd	campsite	surface
531	MP11/1328	21	MP	25,6030	11,9600	hamada	endorheic depression	0	0	0	2	no	0	nd	nd	nd	campsite	surface
532	MP11/1331	21	MP	25,6030	11,9471	hamada	endorheic depression	34	21	13	2	no	21	2 = 10-26	nd	opportunistic	campsite	surface
533	MP11/1333	21	MP	25,5997	11,9430	hamada	desert pavement	41	22	13	2	no	27	3 = 26-50	nd	shaped	campsite	surface
534	MP11/1334	21	MP	25,5994	11,9498	hamada	endorheic depression	0	0	0	2	no	0	nd	black/black	nd	other	building material
535	MP11/1334	21	MP	25,5994	11,9498	hamada	endorheic depression	0	0	0	2	no	0	nd	black/black	nd	other	building material
536	MP11/1335	21	MP	25,5996	11,9526	hamada	endorheic depression	39	30	17	2	no	46	3 = 26-50	black/red	shaped	campsite	surface
537	MP11/1335	21	MP	25,5996	11,9526	hamada	endorheic depression	34	32	15	2	no	38	3 = 26-50	black/red	shaped	campsite	surface
538	MP11/1335	21	MP	25,5996	11,9526	hamada	endorheic depression	34	23	16	2	no	29	3 = 26-50	black/red	shaped	campsite	surface
539	MP11/1349	21	MP	25,5988	11,9547	hamada	endorheic depression	54	18	11	1	no	25	2 = 10-26	nd	shaped	isolated	isolated
540	MP11/135	32	MP	26,0329	11,8604	hamada	endorheic depression	29	14	9	2	no	8	1 =1-10	black/red	shaped	isolated	isolated
541	MP11/1354	21	MP	25,6012	11,9450	hamada	endorheic depression	30	15	15	2	no	16	2 = 10-26	black/black	shaped	isolated	isolated
542	MP11/1355	21	MP	25,6012	11,9442	hamada	endorheic depression	50	37	10	2	circular	43	3 = 26-50	black/red	heavy grooved	campsite	surface
543	MP11/1361	21	MP	25,5976	11,9653	hamada	endorheic depression	28	10	0	2	circular	0	nd	nd	heavy grooved	isolated	isolated
544	MP11/1367	21	MP	25,5895	11,9407	hamada	desert pavement	44	20	10	2	no	20	2 = 10-26	black/red	shaped	campsite	surface
545	MP11/1368	21	MP	25,5894	11,9445	hamada	endorheic depression	42	20	10	2	circular	19	2 = 10-26	black/red	heavy grooved	campsite	surface
546	MP11/1368	21	MP	25,5894	11,9445	hamada	endorheic depression	62	21	10	2	no	30	3 = 26-50	black/red	shaped	campsite	surface
547	MP11/1368	21	MP	25,5894	11,9445	hamada	endorheic depression	30	16	5	2	no	6	1 =1-10	black/red	shaped	campsite	surface
548	MP11/137	32	MP	26,0275	11,8651	hamada	endorheic depression	45	35	15	2	circular	54	4 = 50-85	black/red	heavy grooved	isolated	isolated
549	MP11/1371	21	MP	25,6006	11,9530	hamada	endorheic depression	30	15	9	2	no	9	1 =1-10	black/red	shaped	isolated	isolated
550	MP11/1372	21	MP	25,6004	11,9526	hamada	endorheic depression	38	20	15	2	no	26	3 = 26-50	black/red	shaped	isolated	isolated
551	MP11/1372	21	MP	25,6004	11,9526	hamada	endorheic depression	25	15	10	2	circular	9	1 =1-10	black/red	heavy grooved	isolated	isolated
552	MP11/1373	21	MP	25,6005	11,9495	hamada	endorheic depression	75	20	10	2	no	35	3 = 26-50	black/red	shaped	other	surface
553	MP11/1373	21	MP	25,6005	11,9495	hamada	endorheic depression	45	27	25	2	circular	70	4 = 50-85	black/red	heavy grooved	other	surface
554	MP11/1373	21	MP	25,6005	11,9495	hamada	endorheic depression	45	22	13	2	no	30	3 = 26-50	black/red	shaped	other	surface
555	MP11/1373	21	MP	25,6005	11,9495	hamada	endorheic depression	37	20	10	2	no	17	2 = 10-26	black/red	opportunistic	other	surface
556	MP11/1373	21	MP	25,6005	11,9495	hamada	endorheic depression	38	35	12	2	no	37	3 = 26-50	black/red	shaped	other	surface
557	MP11/1374	21	MP	25,6005	11,9443	hamada	endorheic depression	45	15	10	1	no	16	2 = 10-26	black/red	shaped	isolated	isolated
558	MP11/1375	21	MP	25,5971	11,9265	hamada	endorheic depression	33	22	15	2	no	25	2 = 10-26	black/red	shaped	isolated	isolated
559	MP11/1376	21	MP	25,5971	11,9358	hamada	endorheic depression	50	20	15	2	no	35	3 = 26-50	black/red	opportunistic	isolated	isolated
560	MP11/1379	21	MP	25,5956	11,9642	hamada	endorheic depression	40	15	10	2	no	14	2 = 10-26	black/black	shaped	isolated	isolated
561	MP11/1394	40	MP	25,9766	12,4096	hamada	endorheic depression	44	20	10	2	no	20	2 = 10-26	black/black	opportunistic	isolated	isolated
562	MP11/1397	40	MP	25,9759	12,4463	hamada	endorheic depression	37	17	12	2	no	17	2 = 10-26	black/red	shaped	other	surface
563	MP11/1397	40	MP	25,9759	12,4463	hamada	endorheic depression	25	20	10	2	no	12	2 = 10-26	black/red	shaped	other	surface
564	MP11/1397	40	MP	25,9759	12,4463	hamada	endorheic depression	25	13	10	1	no	7	1 =1-10	black/red	opportunistic	other	surface

565	MP11/1397	40	MP	25,9759	12,4463	hamada	endorheic depression	26	14	9	2	no	8	1 =1-10	black/red	shaped	other	surface
566	MP11/1407	40	MP	25,9709	12,4168	hamada	endorheic depression	30	18	7	2	no	9	1 =1-10	black/red	opportunistic	isolated	isolated
567	MP11/1414	40	MP	25,9714	12,4420	hamada	slope	42	28	12	2	light	32	3 = 26-50	black/black	shaped	other	building material
568	MP11/1443	40	MP	25,9705	12,4150	hamada	endorheic depression	40	25	10	2	no	23	2 = 10-26	black/red	shaped	isolated	isolated
569	MP11/1450	36	MP	26,0160	12,2852	hamada	endorheic depression	40	20	10	2	circular	18	2 = 10-26	black/red	heavy grooved	isolated	isolated
570	MP11/1453	36	MP	26,0151	12,3111	hamada	endorheic depression	50	21	13	2	no	31	3 = 26-50	black/red	opportunistic	isolated	isolated
571	MP11/1454	36	MP	26,0189	12,3165	hamada	endorheic depression	51	30	11	2	no	39	3 = 26-50	black/black	shaped	isolated	isolated
572	MP11/1455	36	MP	26,0187	12,3057	hamada	endorheic depression	43	23	20	2	no	45	3 = 26-50	black/black	shaped	isolated	isolated
573	MP11/1456	36	MP	26,0190	12,3016	hamada	endorheic depression	31	18	15	2	no	19	2 = 10-26	black/black	shaped	isolated	isolated
574	MP11/1466	36	MP	26,0147	12,3071	hamada	endorheic depression	26	12	5	1	no	4	1 =1-10	black/black	opportunistic	campsite	surface
575	MP11/1477	36	MP	26,0160	12,3063	hamada	endorheic depression	0	0	0	2	no	0	nd	black/black	nd	campsite	surface
576	MP11/1477	36	MP	26,0160	12,3063	hamada	endorheic depression	0	0	0	2	no	0	nd	black/black	nd	campsite	surface
577	MP11/1477	36	MP	26,0160	12,3063	hamada	endorheic depression	0	0	0	2	no	0	nd	black/black	nd	campsite	surface
578	MP11/1477	36	MP	26,0160	12,3063	hamada	endorheic depression	0	0	0	2	no	0	nd	black/black	nd	campsite	surface
579	MP11/1477	36	MP	26,0160	12,3063	hamada	endorheic depression	0	0	0	2	no	0	nd	black/black	nd	campsite	surface
580	MP11/1479	36	MP	26,0163	12,2911	hamada	endorheic depression	0	0	0	2	no	0	nd	black/black	nd	campsite	surface
581	MP11/1479	36	MP	26,0163	12,2911	hamada	endorheic depression	0	0	0	2	no	0	nd	black/black	nd	campsite	surface
582	MP11/1479	36	MP	26,0163	12,2911	hamada	endorheic depression	0	0	0	2	no	0	nd	black/black	nd	campsite	surface
583	MP11/1479	36	MP	26,0163	12,2911	hamada	endorheic depression	0	0	0	2	no	0	nd	black/black	nd	campsite	surface
584	MP11/1479	36	MP	26,0163	12,2911	hamada	endorheic depression	0	0	0	2	no	0	nd	black/black	nd	campsite	surface
585	MP11/1482	36	MP	26,0160	12,3145	hamada	endorheic depression	0	0	0	2	no	0	nd	black/black	nd	other	surface
586	MP11/1482	36	MP	26,0160	12,3145	hamada	endorheic depression	0	0	0	2	no	0	nd	black/black	nd	other	surface
587	MP11/1482	36	MP	26,0160	12,3145	hamada	endorheic depression	0	0	0	2	no	0	nd	black/black	nd	other	surface
588	MP11/1482	36	MP	26,0160	12,3145	hamada	endorheic depression	0	0	0	2	no	0	nd	black/black	nd	other	surface
589	MP11/1482	36	MP	26,0160	12,3145	hamada	endorheic depression	0	0	0	2	no	0	nd	black/black	nd	other	surface
590	MP11/1486	36	MP	26,0203	12,3171	hamada	endorheic depression	58	47	7	2	no	44	3 = 26-50	black/black	shaped	campsite	surface
591	MP11/1489	36	MP	26,0210	12,3012	hamada	endorheic depression	28	15	15	2	light	14	2 = 10-26	black/black	shaped	campsite	surface
592	MP11/1489	36	MP	26,0210	12,3012	hamada	endorheic depression	27	17	7	2	no	7	1 =1-10	black/black	shaped	campsite	surface
593	MP11/1489	36	MP	26,0210	12,3012	hamada	endorheic depression	47	29	8	2	no	25	2 = 10-26	black/black	shaped	campsite	surface
594	MP11/1489	36	MP	26,0210	12,3012	hamada	endorheic depression	30	10	10	2	no	7	1 =1-10	black/black	shaped	campsite	surface
595	MP11/1489	36	MP	26,0210	12,3012	hamada	endorheic depression	30	0	10	2	circular	0	nd	nd	heavy grooved	campsite	surface
596	MP11/1501	36	MP	26,0058	12,3031	hamada	endorheic depression	40	18	10	2	no	17	2 = 10-26	black/black	opportunistic	isolated	isolated
597	MP11/1530	36	MP	26,0195	12,3040	hamada	endorheic depression	30	22	11	2	no	17	2 = 10-26	nd	shaped	campsite	surface
598	MP11/1533	36	MP	26,0201	12,2935	hamada	slope	65	36	12	2	circular	65	4 = 50-85	nd	heavy grooved	isolated	isolated
599	MP11/1540	17	MP	25,4115	11,9377	hamada	endorheic depression	43	15	15	2	no	22	2 = 10-26	black/red	shaped	other	surface
600	MP11/1540	17	MP	25,4115	11,9377	hamada	endorheic depression	38	18	13	2	no	20	2 = 10-26	black/red	shaped	other	surface
601	MP11/1540	17	MP	25,4115	11,9377	hamada	endorheic depression	25	15	18	2	no	16	2 = 10-26	black/red	shaped	other	surface
602	MP11/1541	17	MP	25,4115	11,9266	hamada	endorheic depression	40	15	17	2	no	23	2 = 10-26	black/red	shaped	isolated	isolated
603	MP11/1541	17	MP	25,4115	11,9266	hamada	endorheic depression	35	15	10	2	no	12	2 = 10-26	black/red	shaped	isolated	isolated
604	MP11/159	32	MP	26,0431	11,8820	hamada	endorheic depression	0	0	0	2	no	0	nd	black/black	nd	campsite	surface
605	MP11/159	32	MP	26,0431	11,8820	hamada	endorheic depression	0	0	0	2	no	0	nd	black/black	nd	campsite	surface
606	MP11/1590	17	MP	25,4053	11,9162	hamada	endorheic depression	40	27	16	2	no	40	3 = 26-50	black/red	shaped	isolated	isolated
607	MP11/1590	17	MP	25,4053	11,9162	hamada	endorheic depression	32	25	10	2	no	18	2 = 10-26	black/red	shaped	isolated	isolated
608	MP11/1596	12	MP	25,4749	11,5329	hamada	endorheic depression	45	15	8	2	no	12	2 = 10-26	black/red	shaped	isolated	isolated
609	MP11/160	32	MP	26,0436	11,8792	hamada	endorheic depression	45	20	15	2	no	31	3 = 26-50	black/black	shaped	campsite	surface
610	MP11/1628	12	MP	25,4745	11,5376	hamada	endorheic depression	30	15	8	2	no	8	1 =1-10	black/red	shaped	campsite	surface
611	MP11/1628	12	MP	25,4745	11,5376	hamada	endorheic depression	43	20	15	2	no	30	3 = 26-50	black/red	shaped	campsite	surface
612	MP11/1640	14	MP	25,3230	11,6755	hamada	endorheic depression	28	14	14	2	no	13	2 = 10-26	black/red	opportunistic	other	surface
613	MP11/1640	14	MP	25,3230	11,6755	hamada	endorheic depression	24	14	13	2	no	10	2 = 10-26	nd	opportunistic	other	surface
614	MP11/1640	14	MP	25,3230	11,6755	hamada	endorheic depression	66	23	13	2	circular	45	3 = 26-50	black/red	heavy grooved	other	surface
615	MP11/1640	14	MP	25,3230	11,6755	hamada	endorheic depression	48	21	7	1	no	16	2 = 10-26	nd	opportunistic	other	surface
616	MP11/1643	14	MP	25,3207	11,7073	hamada	endorheic depression	0	0	0	2	no	0	nd	nd	nd	campsite	building material
617	MP11/1656	14	MP	25,3235	11,7031	hamada	endorheic depression	75	30	10	2	circular	52	4 = 50-85	black/red	heavy grooved	isolated	isolated
618	MP11/1664	14	MP	25,3224	11,7082	hamada	endorheic depression	35	15	10	2	no	12	2 = 10-26	black/red	nd	isolated	isolated
619	MP11/1665	14	MP	25,3226	11,7069	hamada	endorheic depression	45	20	10	2	no	21	2 = 10-26	black/red	opportunistic	isolated	isolated
620	MP11/167	-	MP	26,0461	11,8667	hamada	desert pavement	27	17	7	2	light	7	1 =1-10	black/black	shaped	other	surface
621	MP11/167	-	MP	26,0461	11,8667	hamada	desert pavement	43	12	7	2	no	8	1 =1-10	black/black	shaped	other	surface
622	MP11/167	-	MP	26,0461	11,8667	hamada	desert pavement	40	19	8	2	no	14	2 = 10-26	black/black	shaped	other	surface
623	MP11/167	-	MP	26,0461	11,8667	hamada	desert pavement	25	16	10	2	no	9	1 =1-10	black/black	shaped	other	surface
624	MP11/167	-	MP	26,0461	11,8667	hamada	desert pavement	40	17	12	2	circular	19	2 = 10-26	black/black	heavy grooved	other	surface
625	MP11/1670	14	MP	25,3249	11,6782	hamada	desert pavement	27	27	12	2	no	20	2 = 10-26	black/red	shaped	campsite	surface
626	MP11/1670	14	MP	25,3249	11,6782	hamada	desert pavement	80	40	30	2	circular	221	6 = 146-295	black/red	heavy grooved	campsite	surface
627	MP11/1670	14	MP	25,3249	11,6782	hamada	desert pavement	40	20	15	2	no	28	3 = 26-50	black/red	shaped	campsite	surface

628	MP11/1679	13	MP	25,4100	11,6270	hamada	slope	53	23	10	2	no	28	3 = 26-50	black/black	shaped	isolated	isolated
629	MP11/1684	13	MP	25,4079	11,6365	hamada	desert pavement	35	25	10	2	circular	20	2 = 10-26	black/red	heavy grooved	campsite	surface
630	MP11/1684	13	MP	25,4079	11,6365	hamada	desert pavement	35	25	8	2	no	16	2 = 10-26	black/black	shaped	campsite	surface
631	MP11/1684	13	MP	25,4079	11,6365	hamada	desert pavement	25	20	8	2	no	9	1 =1-10	black/red	shaped	campsite	surface
632	MP11/1714	13	MP	25,4030	11,6378	hamada	endorheic depression	47	17	12	2	no	22	2 = 10-26	black/red	opportunistic	campsite	surface
633	MP11/1718	41	MP	26,2499	12,3491	hamada	endorheic depression	50	25	18	2	circular	52	4 = 50-85	black/black	heavy grooved	campsite	surface
634	MP11/1725	41	MP	26,2541	12,3646	hamada	desert pavement	55	31	18	2	circular	71	4 = 50-85	black/red	heavy grooved	isolated	isolated
635	MP11/1734	41	MP	26,2521	12,3326	hamada	endorheic depression	30	15	10	2	no	10	2 = 10-26	black/red	shaped	isolated	isolated
636	MP11/1735	41	MP	26,2521	12,3352	hamada	endorheic depression	35	10	10	2	no	8	1 =1-10	black/black	opportunistic	isolated	isolated
637	MP11/174	32	MP	26,0299	11,8626	hamada	endorheic depression	30	23	8	2	no	13	2 = 10-26	black/red	shaped	campsite	surface
638	MP11/174	32	MP	26,0299	11,8626	hamada	endorheic depression	49	20	10	2	circular	23	2 = 10-26	black/red	heavy grooved	campsite	surface
639	MP11/174	32	MP	26,0299	11,8626	hamada	endorheic depression	33	21	8	2	no	13	2 = 10-26	black/red	shaped	campsite	surface
640	MP11/174	32	MP	26,0299	11,8626	hamada	endorheic depression	49	18	10	2	no	20	2 = 10-26	black/red	shaped	campsite	surface
641	MP11/1740	41	MP	26,2510	12,3689	hamada	endorheic depression	30	20	10	2	no	14	2 = 10-26	black/black	shaped	campsite	surface
642	MP11/176	32	MP	26,0291	11,8806	hamada	desert pavement	30	20	10	2	no	14	2 = 10-26	nd	opportunistic	other	building material
643	MP11/176	32	MP	26,0291	11,8806	hamada	desert pavement	37	12	0	2	circular	0	nd	nd	heavy grooved	campsite	surface
644	MP11/1771	29	MP	25,9557	12,0565	hamada	endorheic depression	36	18	21	2	no	31	3 = 26-50	black/red	shaped	isolated	isolated
645	MP11/1779	29	MP	25,9579	12,0626	hamada	endorheic depression	35	15	9	2	no	11	2 = 10-26	black/red	shaped	campsite	surface
646	MP11/1787	29	MP	25,9468	12,0657	hamada	desert pavement	40	21	8	2	light	15	2 = 10-26	black/black	shaped	campsite	surface
647	MP11/1811	44	MP	26,3322	12,5420	hamada	endorheic depression	45	20	25	2	no	52	4 = 50-85	black/red	shaped	isolated	isolated
648	MP11/1813	44	MP	26,3281	12,5464	hamada	desert pavement	35	25	8	2	no	16	2 = 10-26	black/red	shaped	isolated	isolated
649	MP11/1849	47	MP	26,3773	12,8647	hamada	endorheic depression	47	20	14	2	no	30	3 = 26-50	black/red	shaped	isolated	isolated
650	MP11/1852	47	MP	26,3793	12,8660	hamada	endorheic depression	33	18	12	2	no	16	2 = 10-26	black/red	opportunistic	isolated	isolated
651	MP11/1853	47	MP	26,3752	12,8757	hamada	desert pavement	51	18	8	2	no	17	2 = 10-26	black/red	shaped	isolated	isolated
652	MP11/1854	47	MP	26,3755	12,8682	hamada	endorheic depression	20	15	8	2	no	6	1 =1-10	black/red	shaped	isolated	surface
653	MP11/1882	47	MP	26,3784	12,8747	hamada	endorheic depression	30	25	15	2	no	26	2 = 10-26	black/black	shaped	isolated	isolated
654	MP11/1910	46	MP	26,3125	12,7293	hamada	endorheic depression	20	15	8	2	no	6	1 =1-10	nd	nd	campsite	surface
655	MP11/1910	46	MP	26,3125	12,7293	hamada	endorheic depression	25	10	8	2	no	5	1 =1-10	nd	nd	campsite	surface
656	MP11/1911	46	MP	26,3123	12,7359	hamada	endorheic depression	35	20	10	2	no	16	2 = 10-26	black/red	shaped	isolated	isolated
657	MP11/1919	46	MP	26,3258	12,7135	hamada	endorheic depression	44	22	10	2	no	22	2 = 10-26	nd	opportunistic	isolated	isolated
658	MP11/1920	46	MP	26,3256	12,7263	hamada	desert pavement	70	67	0	2	circular	0	nd	nd	heavy grooved	isolated	isolated
659	MP11/1930	46	MP	26,3282	12,7226	hamada	endorheic depression	96	20	10	2	no	44	3 = 26-50	nd	opportunistic	campsite	surface
660	MP11/1930	46	MP	26,3282	12,7226	hamada	endorheic depression	44	37	5	2	no	19	2 = 10-26	nd	shaped	campsite	surface
661	MP11/1930	46	MP	26,3282	12,7226	hamada	endorheic depression	49	14	7	1	no	11	2 = 10-26	nd	shaped	campsite	surface
662	MP11/1930	46	MP	26,3282	12,7226	hamada	endorheic depression	63	17	7	2	no	17	2 = 10-26	nd	shaped	campsite	surface
663	MP11/195	32	MP	26,0302	11,8735	hamada	endorheic depression	46	18	15	2	no	29	3 = 26-50	nd	shaped	isolated	isolated
664	MP11/240	28	MP	25,9135	11,8855	hamada	endorheic depression	51	23	10	2	circular	27	3 = 26-50	black/black	heavy grooved	campsite	surface
665	MP11/255	28	MP	25,9169	11,8883	hamada	endorheic depression	35	14	7	2	no	8	1 =1-10	black/red	opportunistic	isolated	isolated
666	MP11/261	28	MP	25,9072	11,9066	hamada	desert pavement	37	20	10	2	no	17	2 = 10-26	black/black	shaped	campsite	surface
667	MP11/267	28	MP	25,9004	11,8785	hamada	endorheic depression	32	17	10	2	circular	13	2 = 10-26	black/black	heavy grooved	isolated	isolated
668	MP11/268	28	MP	25,9004	11,9099	hamada	endorheic depression	33	20	8	2	circular	12	2 = 10-26	black/black	heavy grooved	isolated	isolated
669	MP11/268	28	MP	25,9004	11,9099	hamada	endorheic depression	42	17	5	1	no	8	1 =1-10	black/black	shaped	isolated	isolated
670	MP11/273	28	MP	25,9095	11,8810	hamada	endorheic depression	40	20	18	2	circular	33	3 = 26-50	black/black	heavy grooved	isolated	isolated
671	MP11/366	39	MP	26,1235	12,2792	hamada	endorheic depression	37	19	8	2	no	13	2 = 10-26	black/red	shaped	isolated	isolated
672	MP11/369	39	MP	26,1231	12,2965	hamada	endorheic depression	30	22	8	2	no	12	2 = 10-26	black/red	shaped	isolated	isolated
673	MP11/370	39	MP	26,1230	12,3059	hamada	endorheic depression	48	22	15	2	circular	36	3 = 26-50	nd	heavy grooved	campsite	surface
674	MP11/371	39	MP	26,1182	12,3025	hamada	endorheic depression	25	18	10	2	no	10	2 = 10-26	black/red	shaped	campsite	surface
675	MP11/372	39	MP	26,1185	12,2990	hamada	endorheic depression	42	19	8	2	no	15	2 = 10-26	black/red	shaped	isolated	isolated
676	MP11/373	39	MP	26,1185	12,2985	hamada	endorheic depression	32	17	8	2	circular	10	2 = 10-26	black/black	heavy grooved	isolated	isolated
677	MP11/373	39	MP	26,1185	12,2985	hamada	endorheic depression	50	25	15	2	circular	43	3 = 26-50	nd	heavy grooved	isolated	isolated
678	MP11/374	39	MP	26,1187	12,2942	hamada	endorheic depression	32	20	10	2	circular	15	2 = 10-26	black/red	heavy grooved	campsite	surface
679	MP11/393	39	MP	26,1226	12,2779	hamada	endorheic depression	52	28	14	2	light	47	3 = 26-50	black/black	shaped	campsite	surface
680	MP11/395	39	MP	26,1222	12,2931	hamada	endorheic depression	28	13	13	2	no	11	2 = 10-26	black/black	shaped	isolated	isolated
681	MP11/395	39	MP	26,1222	12,2931	hamada	endorheic depression	25	15	10	2	circular	9	1 =1-10	nd	heavy grooved	isolated	isolated
682	MP11/396	39	MP	26,1222	12,3028	hamada	desert pavement	42	31	19	2	no	57	4 = 50-85	nd	shaped	campsite	surface
683	MP11/399	39	MP	26,1166	12,3034	hamada	endorheic depression	28	14	13	2	no	12	2 = 10-26	black/red	shaped	isolated	isolated
684	MP11/400	39	MP	26,1165	12,2981	hamada	endorheic depression	34	17	18	2	no	24	2 = 10-26	black/red	shaped	campsite	surface
685	MP11/403	39	MP	26,1205	12,2876	hamada	endorheic depression	45	20	8	2	no	17	2 = 10-26	black/red	shaped	campsite	surface
686	MP11/406	39	MP	26,1208	12,2923	hamada	endorheic depression	30	13	10	2	no	9	1 =1-10	black/black	shaped	campsite	surface
687	MP11/407	39	MP	26,1209	12,2943	hamada	endorheic depression	25	14	10	2	no	8	1 =1-10	black/black	shaped	campsite	surface
688	MP11/409	39	MP	26,1203	12,3134	hamada	endorheic depression	31	19	10	2	no	14	2 = 10-26	black/red	shaped	isolated	isolated
689	MP11/410	39	MP	26,1155	12,3051	hamada	endorheic depression	32	17	10	2	no	13	2 = 10-26	black/black	opportunistic	campsite	surface
690	MP11/411	39	MP	26,1152	12,3048	hamada	endorheic depression	40	19	10	2	no	17	2 = 10-26	black/black	shaped	campsite	surface

691	MP11/412	39	MP	26,1163	12,2801	hamada	endorheic depression	32	15	15	2	circular	17	2 = 10-26	black/red	heavy grooved	isolated	isolated
692	MP11/412	39	MP	26,1163	12,2801	hamada	endorheic depression	42	30	10	2	circular	29	3 = 26-50	black/red	heavy grooved	isolated	isolated
693	MP11/416	39	MP	26,1146	12,2887	hamada	endorheic depression	30	10	10	2	light	7	1 =1-10	black/red	nd	campsite	surface
694	MP11/416	39	MP	26,1146	12,2887	hamada	endorheic depression	40	20	10	2	no	18	2 = 10-26	black/red	shaped	campsite	surface
695	MP11/417	39	MP	26,1126	12,3123	hamada	endorheic depression	35	15	10	2	no	12	2 = 10-26	black/red	shaped	campsite	surface
696	MP11/417	39	MP	26,1126	12,3123	hamada	endorheic depression	16	11	10	2	no	4	1 =1-10	black/red	shaped	campsite	surface
697	MP11/420	-	MP	26,1055	12,2972	hamada	endorheic depression	31	20	10	2	no	14	2 = 10-26	black/black	shaped	campsite	surface
698	MP11/420	-	MP	26,1055	12,2972	hamada	endorheic depression	30	17	20	2	no	23	2 = 10-26	black/black	shaped	campsite	surface
699	MP11/421	-	MP	26,1051	12,2895	hamada	endorheic depression	28	14	14	2	no	13	2 = 10-26	black/black	shaped	other	surface
700	MP11/421	-	MP	26,1051	12,2895	hamada	endorheic depression	42	18	16	2	no	28	3 = 26-50	black/black	shaped	other	surface
701	MP11/421	-	MP	26,1051	12,2895	hamada	endorheic depression	40	24	14	2	no	31	3 = 26-50	black/black	shaped	other	surface
702	MP11/444	30	MP	25,7985	12,1426	hamada	endorheic depression	50	20	10	2	no	23	2 = 10-26	black/black	shaped	isolated	isolated
703	MP11/451	30	MP	25,8066	12,1399	hamada	desert pavement	47	33	9	2	no	32	3 = 26-50	nd	opportunistic	isolated	isolated
704	MP11/453	30	MP	25,7994	12,1433	hamada	desert pavement	23	15	9	2	no	7	1 =1-10	black/red	shaped	campsite	surface
705	MP11/524	31	MP	26,0636	11,6893	hamada	slope	37	17	6	2	no	9	1 =1-10	black/black	shaped	isolated	isolated
706	MP11/547	31	MP	26,0565	11,7241	hamada	desert pavement	64	30	10	2	circular	44	3 = 26-50	black/red	heavy grooved	isolated	isolated
707	MP11/547	31	MP	26,0565	11,7241	hamada	desert pavement	58	30	10	2	no	40	3 = 26-50	black/red	opportunistic	isolated	isolated
708	MP11/570	31	MP	26,0605	11,7212	hamada	endorheic depression	40	15	7	2	circular	10	1 =1-10	black/black	heavy grooved	isolated	isolated
709	MP11/583	31	MP	26,0592	11,7257	hamada	endorheic depression	38	34	14	2	no	42	3 = 26-50	black/red	shaped	isolated	isolated
710	MP11/588	27	MP	25,9741	11,7301	hamada	slope	20	18	8	2	light	7	1 =1-10	black/black	shaped	isolated	isolated
711	MP11/599	27	MP	25,9721	11,7341	hamada	desert pavement	45	31	17	2	no	55	4 = 50-85	black/red	shaped	isolated	isolated
712	MP11/600	27	MP	25,9721	11,7309	hamada	endorheic depression	32	12	8	2	no	7	1 =1-10	black/red	shaped	isolated	isolated
713	MP11/603	27	MP	25,9684	11,7343	hamada	endorheic depression	45	26	15	2	no	40	3 = 26-50	black/red	shaped	other	surface
714	MP11/603	27	MP	25,9684	11,7343	hamada	endorheic depression	46	29	12	2	no	37	3 = 26-50	black/red	shaped	other	surface
715	MP11/603	27	MP	25,9684	11,7343	hamada	endorheic depression	30	30	15	2	no	31	3 = 26-50	black/red	shaped	other	surface
716	MP11/605	27	MP	25,9686	11,7408	hamada	endorheic depression	40	20	16	2	no	29	3 = 26-50	black/red	shaped	isolated	isolated
717	MP11/605	27	MP	25,9686	11,7408	hamada	endorheic depression	25	15	10	2	no	9	1 =1-10	black/red	shaped	isolated	isolated
718	MP11/607	27	MP	25,9683	11,7519	hamada	endorheic depression	33	20	15	2	no	23	2 = 10-26	black/red	shaped	isolated	isolated
719	MP11/609	27	MP	25,9712	11,7340	hamada	desert pavement	30	17	9	2	no	11	2 = 10-26	black/black	opportunistic	isolated	isolated
720	MP11/610	27	MP	25,9714	11,7315	hamada	endorheic depression	46	17	10	2	no	18	2 = 10-26	black/red	shaped	campsite	surface
721	MP11/610	27	MP	25,9714	11,7315	hamada	endorheic depression	40	19	10	2	no	17	2 = 10-26	black/red	shaped	campsite	surface
722	MP11/610	27	MP	25,9714	11,7315	hamada	endorheic depression	63	22	15	2	no	48	3 = 26-50	black/red	shaped	campsite	surface
723	MP11/634	27	MP	25,9670	11,7354	hamada	endorheic depression	50	24	10	2	no	28	3 = 26-50	nd	opportunistic	isolated	isolated
724	MP11/635	27	MP	25,9668	11,7380	hamada	endorheic depression	34	25	8	2	no	16	2 = 10-26	nd	shaped	isolated	isolated
725	MP11/635	27	MP	25,9668	11,7380	hamada	endorheic depression	50	19	10	2	no	22	2 = 10-26	nd	shaped	isolated	isolated
726	MP11/640	27	MP	25,9630	11,7435	hamada	desert pavement	30	15	20	2	circular	21	2 = 10-26	black/red	heavy grooved	isolated	isolated
727	MP11/647	27	MP	25,9623	11,7184	hamada	slope	45	15	10	2	circular	16	2 = 10-26	nd	heavy grooved	campsite	building material
728	MP11/653	24	MP	25,8778	11,7662	hamada	desert pavement	54	40	0	2	circular	0	nd	black/red	heavy grooved	isolated	isolated
729	MP11/654	24	MP	25,8784	11,7614	hamada	endorheic depression	35	17	11	2	no	15	2 = 10-26	black/black	shaped	campsite	surface
730	MP11/655	24	MP	25,8780	11,7592	hamada	desert pavement	70	30	5	2	no	24	2 = 10-26	black/red	shaped	campsite	surface
731	MP11/656	24	MP	25,8783	11,7470	hamada	desert pavement	55	40	30	2	circular	152	6 = 146-295	black/red	heavy grooved	isolated	isolated
732	MP11/658	24	MP	25,8834	11,7420	hamada	endorheic depression	40	22	10	2	no	20	2 = 10-26	black/red	shaped	isolated	isolated
733	MP11/658	24	MP	25,8834	11,7420	hamada	endorheic depression	40	30	18	2	no	50	3 = 26-50	black/red	shaped	isolated	isolated
734	MP11/671	24	MP	25,8847	11,7462	hamada	desert pavement	53	26	10	2	light	32	3 = 26-50	black/black	shaped	campsite	building material
735	MP11/679	24	MP	25,8793	11,7604	hamada	endorheic depression	50	20	10	2	circular	23	2 = 10-26	black/black	heavy grooved	campsite	surface
736	MP11/684	24	MP	25,8828	11,7491	hamada	endorheic depression	34	19	12	2	no	18	2 = 10-26	black/black	shaped	isolated	isolated
737	MP11/684	24	MP	25,8828	11,7491	hamada	endorheic depression	0	0	0	2	circular	0	nd	nd	heavy grooved	isolated	isolated
738	MP11/684	24	MP	25,8828	11,7491	hamada	endorheic depression	37	20	12	2	no	20	2 = 10-26	nd	shaped	isolated	isolated
739	MP11/696	24	MP	25,8693	11,7772	hamada	endorheic depression	58	35	20	2	circular	93	5 = 85-146	black/red	heavy grooved	campsite	surface
740	MP11/696	24	MP	25,8693	11,7772	hamada	endorheic depression	42	32	12	2	light	37	3 = 26-50	black/red	shaped	campsite	surface
741	MP11/720	24	MP	25,8797	11,7611	hamada	endorheic depression	40	20	10	2	no	18	2 = 10-26	black/red	shaped	campsite	surface
742	MP11/720	24	MP	25,8797	11,7611	hamada	endorheic depression	50	25	21	2	no	60	4 = 50-85	black/red	shaped	campsite	surface
743	MP11/720	24	MP	25,8797	11,7611	hamada	endorheic depression	43	15	12	2	no	18	2 = 10-26	black/red	shaped	campsite	surface
744	MP11/721	24	MP	25,8798	11,7567	hamada	endorheic depression	32	26	16	2	no	31	3 = 26-50	black/red	shaped	isolated	isolated
745	MP11/721	24	MP	25,8798	11,7567	hamada	endorheic depression	60	30	16	2	no	66	4 = 50-85	nd	shaped	isolated	isolated
746	MP11/726	24	MP	25,8854	11,7591	hamada	endorheic depression	30	21	9	2	no	13	2 = 10-26	black/red	shaped	isolated	isolated
747	MP11/727	24	MP	25,8852	11,7633	hamada	endorheic depression	35	20	10	2	no	16	2 = 10-26	black/black	shaped	isolated	isolated
748	MP11/743	33	MP	25,8991	12,1504	hamada	endorheic depression	35	25	10	2	no	20	2 = 10-26	black/red	shaped	isolated	isolated
749	MP11/767	33	MP	25,8891	12,1463	hamada	endorheic depression	45	30	19	2	no	59	4 = 50-85	black/red	shaped	campsite	surface
750	MP11/767	33	MP	25,8891	12,1463	hamada	endorheic depression	0	0	0	2	no	0	nd	black/red	nd	campsite	surface
751	MP11/767	33	MP	25,8891	12,1463	hamada	endorheic depression	0	0	0	2	no	0	nd	black/red	nd	campsite	surface
752	MP11/767	33	MP	25,8891	12,1463	hamada	endorheic depression	0	0	0	2	no	0	nd	black/red	nd	campsite	surface
753	MP11/767	33	MP	25,8891	12,1463	hamada	endorheic depression	0	0	0	2	no	0	nd	black/red	nd	campsite	surface

754	MP11/767	33	MP	25,8891	12,1463	hamada	endorheic depression	0	0	0	2	no	0	nd	black/red	nd	campsite	surface
755	MP11/767	33	MP	25,8891	12,1463	hamada	endorheic depression	0	0	0	2	no	0	nd	black/red	nd	campsite	surface
756	MP11/767	33	MP	25,8891	12,1463	hamada	endorheic depression	0	0	0	2	no	0	nd	black/red	nd	campsite	surface
757	MP11/767	33	MP	25,8891	12,1463	hamada	endorheic depression	0	0	0	2	no	0	nd	black/red	nd	campsite	surface
758	MP11/767	33	MP	25,8891	12,1463	hamada	endorheic depression	0	0	0	2	no	0	nd	black/red	nd	campsite	surface
759	MP11/768	33	MP	25,8884	12,1540	hamada	endorheic depression	35	18	12	2	no	17	2 = 10-26	nd	opportunistic	campsite	surface
760	MP11/768	33	MP	25,8884	12,1540	hamada	endorheic depression	35	25	13	2	circular	26	3 = 26-50	nd	heavy grooved	campsite	surface
761	MP11/768	33	MP	25,8884	12,1540	hamada	endorheic depression	45	23	10	2	no	24	2 = 10-26	nd	opportunistic	campsite	surface
762	MP11/768	33	MP	25,8884	12,1540	hamada	endorheic depression	40	10	12	2	circular	11	2 = 10-26	nd	heavy grooved	campsite	surface
763	MP11/768	33	MP	25,8884	12,1540	hamada	endorheic depression	30	15	15	2	no	16	2 = 10-26	nd	opportunistic	campsite	surface
764	MP11/770	33	MP	25,8847	12,1622	hamada	endorheic depression	35	22	15	2	no	27	3 = 26-50	black/red	shaped	campsite	surface
765	MP11/770	33	MP	25,8847	12,1622	hamada	endorheic depression	0	0	0	2	no	0	nd	nd	nd	campsite	surface
766	MP11/770	33	MP	25,8847	12,1622	hamada	endorheic depression	0	0	0	2	no	0	nd	nd	nd	campsite	surface
767	MP11/771	33	MP	25,8844	12,1580	hamada	endorheic depression	45	27	9	2	circular	25	2 = 10-26	black/red	heavy grooved	campsite	surface
768	MP11/771	33	MP	25,8844	12,1580	hamada	endorheic depression	0	0	0	2	no	0	nd	black/red	nd	campsite	surface
769	MP11/771	33	MP	25,8844	12,1580	hamada	endorheic depression	0	0	0	2	no	0	nd	black/red	nd	campsite	surface
770	MP11/773	33	MP	25,9000	12,1328	hamada	endorheic depression	29	18	11	2	no	13	2 = 10-26	black/red	shaped	isolated	isolated
771	MP11/774	33	MP	25,9003	12,1405	hamada	endorheic depression	35	15	10	2	no	12	2 = 10-26	black/black	shaped	campsite	surface
772	MP11/782	38	MP	26,2375	12,1745	hamada	endorheic depression	45	24	18	1	no	45	3 = 26-50	black/black	shaped	isolated	isolated
773	MP11/783	38	MP	26,2366	12,1819	hamada	endorheic depression	35	18	14	2	no	20	2 = 10-26	black/black	nd	isolated	isolated
774	MP11/784	38	MP	26,2370	12,1832	hamada	endorheic depression	40	22	17	2	no	34	3 = 26-50	black/black	shaped	isolated	isolated
775	MP11/786	38	MP	26,2434	12,1816	hamada	desert pavement	0	0	0	2	no	0	nd	black/red	shaped	campsite	building material
776	MP11/787	38	MP	26,2430	12,1780	hamada	endorheic depression	48	35	15	2	circular	58	4 = 50-85	black/red	heavy grooved	isolated	isolated
777	MP11/788	38	MP	26,2434	12,1684	hamada	desert pavement	35	16	13	2	circular	17	2 = 10-26	black/red	heavy grooved	isolated	isolated
778	MP11/789	38	MP	26,2435	12,1681	hamada	endorheic depression	35	23	10	2	no	19	2 = 10-26	black/red	shaped	isolated	isolated
779	MP11/796	38	MP	26,2416	12,1724	hamada	endorheic depression	23	13	6	2	no	4	1 =1-10	black/black	shaped	isolated	isolated
780	MP11/798	38	MP	26,2419	12,1710	hamada	endorheic depression	83	45	0	2	circular	0	nd	nd	heavy grooved	campsite	surface
781	MP11/800	38	MP	26,2417	12,1614	hamada	endorheic depression	40	20	8	2	no	15	2 = 10-26	nd	shaped	isolated	isolated
782	MP11/800	38	MP	26,2417	12,1614	hamada	endorheic depression	37	22	15	2	no	28	3 = 26-50	nd	shaped	isolated	isolated
783	MP11/801	38	MP	26,2514	12,1616	hamada	endorheic depression	33	22	7	2	no	12	2 = 10-26	black/black	shaped	campsite	surface
784	MP11/812	-	MP	26,2496	12,1615	hamada	endorheic depression	44	17	5	2	no	9	1 =1-10	nd	shaped	campsite	surface
785	MP11/819	38	MP	26,2418	12,1781	hamada	desert pavement	0	0	0	2	no	0	nd	nd	shaped	isolated	isolated
786	MP11/820	38	MP	26,2422	12,1721	hamada	endorheic depression	40	18	8	2	no	13	2 = 10-26	nd	shaped	campsite	surface
787	MP11/846	38	MP	26,2394	12,1712	hamada	endorheic depression	18	12	10	2	no	5	1 =1-10	black/red	shaped	other	surface
788	MP11/846	38	MP	26,2394	12,1712	hamada	endorheic depression	33	16	12	2	no	15	2 = 10-26	black/red	opportunistic	other	surface
789	MP11/846	38	MP	26,2394	12,1712	hamada	endorheic depression	35	16	5	2	circular	6	1 =1-10	black/red	heavy grooved	other	surface
790	MP11/846	38	MP	26,2394	12,1712	hamada	endorheic depression	40	17	5	2	no	8	1 =1-10	black/red	shaped	other	surface
791	MP11/846	38	MP	26,2394	12,1712	hamada	endorheic depression	24	15	7	2	no	6	1 =1-10	black/red	shaped	other	surface
792	MP11/849	38	MP	26,2386	12,1859	hamada	endorheic depression	27	12	8	2	no	6	1 =1-10	nd	shaped	campsite	surface
793	MP11/849	38	MP	26,2386	12,1859	hamada	endorheic depression	42	22	10	2	no	21	2 = 10-26	nd	shaped	campsite	surface
794	MP11/849	38	MP	26,2386	12,1859	hamada	endorheic depression	63	40	10	2	no	58	4 = 50-85	nd	shaped	campsite	surface
795	MP11/849	38	MP	26,2386	12,1859	hamada	endorheic depression	40	25	10	2	circular	23	2 = 10-26	black/red	heavy grooved	campsite	surface
796	MP11/849	38	MP	26,2386	12,1859	hamada	endorheic depression	55	35	0	2	circular	0	nd	nd	heavy grooved	campsite	surface
797	MP11/849	38	MP	26,2386	12,1859	hamada	endorheic depression	35	15	10	2	no	12	2 = 10-26	black/red	shaped	campsite	surface
798	MP11/849	38	MP	26,2386	12,1859	hamada	endorheic depression	45	20	10	2	circular	21	2 = 10-26	nd	heavy grooved	campsite	surface
799	MP11/849	38	MP	26,2386	12,1859	hamada	endorheic depression	35	18	10	2	no	14	2 = 10-26	nd	shaped	campsite	surface
800	MP11/851	38	MP	26,2440	12,1741	hamada	endorheic depression	27	19	14	2	no	17	2 = 10-26	black/red	shaped	isolated	surface
801	MP11/851	38	MP	26,2440	12,1741	hamada	endorheic depression	60	20	10	2	no	28	3 = 26-50	black/red	shaped	isolated	surface
802	MP11/855	38	MP	26,2377	12,1624	hamada	endorheic depression	60	32	15	2	no	66	4 = 50-85	black/black	shaped	other	surface
803	MP11/855	38	MP	26,2377	12,1624	hamada	endorheic depression	48	23	8	2	no	20	2 = 10-26	black/black	shaped	other	surface
804	MP11/855	38	MP	26,2377	12,1624	hamada	endorheic depression	25	18	16	2	no	17	2 = 10-26	black/black	shaped	other	surface
805	MP11/857	37	MP	25,9040	12,3142	hamada	endorheic depression	39	9	10	2	no	8	1 =1-10	black/red	shaped	other	surface
806	MP11/857	37	MP	25,9040	12,3142	hamada	endorheic depression	30	25	10	2	no	17	2 = 10-26	black/red	shaped	other	surface
807	MP11/860	37	MP	25,9035	12,3333	hamada	slope	43	13	10	2	light	13	2 = 10-26	black/red	shaped	isolated	isolated
808	MP11/861	37	MP	25,9086	12,3269	hamada	endorheic depression	47	16	10	2	circular	17	2 = 10-26	black/red	heavy grooved	isolated	isolated
809	MP11/862	37	MP	25,9086	12,3198	hamada	endorheic depression	37	13	8	2	circular	9	1 =1-10	black/black	heavy grooved	isolated	isolated
810	MP11/894	37	MP	25,9062	12,3236	hamada	endorheic depression	27	16	13	1	no	13	2 = 10-26	nd	shaped	isolated	isolated
811	MP11/897	37	MP	25,9029	12,3136	hamada	endorheic depression	37	28	10	2	no	24	2 = 10-26	black/red	shaped	isolated	isolated
812	MP11/899	37	MP	25,9030	12,3216	hamada	endorheic depression	30	21	9	2	no	13	2 = 10-26	black/black	shaped	isolated	isolated
813	MP11/901	37	MP	25,9029	12,3264	hamada	desert pavement	30	10	7	2	no	5	1 =1-10	black/black	shaped	campsite	building material
814	MP11/919	37	MP	25,9011	12,3153	hamada	endorheic depression	45	25	10	2	no	26	2 = 10-26	black/black	shaped	campsite	surface
815	MP11/921	37	MP	25,9009	12,3198	hamada	endorheic depression	0	0	0	2	no	0	nd	nd	nd	campsite	surface

816	MP11/921	37	MP	25,9009	12,3198	hamada	endorheic depression	0	0	0	2	no	0	nd	nd	nd	campsite	surface
817	MP11/921	37	MP	25,9009	12,3198	hamada	endorheic depression	0	0	0	2	no	0	nd	nd	nd	campsite	surface
818	MP11/921	37	MP	25,9009	12,3198	hamada	endorheic depression	0	0	0	2	no	0	nd	nd	nd	campsite	surface
819	MP11/921	37	MP	25,9009	12,3198	hamada	endorheic depression	0	0	0	2	no	0	nd	nd	nd	campsite	surface
820	MP11/924	37	MP	25,9007	12,3301	hamada	endorheic depression	33	14	12	2	no	13	2 = 10-26	black/black	shaped	campsite	surface
821	MP11/929	37	MP	25,9073	12,3159	hamada	endorheic depression	46	24	22	2	no	56	4 = 50-85	black/black	opportunistic	campsite	surface
822	MP11/929	37	MP	25,9073	12,3159	hamada	endorheic depression	0	0	0	2	no	0	nd	nd	nd	campsite	building material
823	MP11/929	37	MP	25,9073	12,3159	hamada	endorheic depression	0	0	0	2	no	0	nd	nd	nd	campsite	building material
824	MP11/929	37	MP	25,9073	12,3159	hamada	endorheic depression	0	0	0	2	no	0	nd	nd	nd	campsite	building material
825	MP11/929	37	MP	25,9073	12,3159	hamada	endorheic depression	0	0	0	2	no	0	nd	nd	nd	campsite	building material
826	MP11/933	15	MP	25,5686	11,4681	hamada	endorheic depression	25	17	10	2	no	10	1 =1-10	black/black	shaped	other	building material
827	MP11/938	15	MP	25,5713	11,4669	hamada	endorheic depression	39	23	10	2	circular	21	2 = 10-26	nd	heavy grooved	isolated	isolated
828	MP11/946	-	MP	25,5701	11,4931	hamada	desert pavement	55	40	15	2	no	76	4 = 50-85	nd	shaped	isolated	isolated
829	MP11/948	15	MP	25,5701	11,4535	hamada	endorheic depression	40	16	8	2	no	12	2 = 10-26	black/black	opportunistic	isolated	isolated
830	MP11/949	15	MP	25,5701	11,4579	hamada	endorheic depression	25	18	12	2	no	12	2 = 10-26	black/black	shaped	isolated	isolated
831	MP11/953	15	MP	25,5694	11,4816	hamada	desert pavement	35	21	11	2	circular	19	2 = 10-26	black/black	heavy grooved	isolated	isolated
832	MP11/974	15	MP	25,5675	11,4635	hamada	endorheic depression	30	16	7	2	no	8	1 =1-10	black/black	shaped	isolated	isolated
833	MP11/978	15	MP	25,5739	11,4629	hamada	endorheic depression	30	21	10	2	no	14	2 = 10-26	black/black	opportunistic	isolated	isolated
834	MP11/981	15	MP	25,5704	11,4646	hamada	desert pavement	42	23	5	2	circular	11	2 = 10-26	black/red	heavy grooved	isolated	isolated
835	MP11/985	-	MP	25,7179	11,6252	hamada	endorheic depression	40	22	15	2	circular	30	3 = 26-50	black/red	heavy grooved	campsite	surface
836	MP11/986	-	MP	25,7198	11,6342	hamada	endorheic depression	43	25	15	2	circular	37	3 = 26-50	black/red	heavy grooved	isolated	isolated
837	MP11/989	20	MP	25,7037	11,6491	hamada	endorheic depression	48	24	11	2	no	29	3 = 26-50	black/red	shaped	isolated	isolated

* the weight has been calculated using size and sandstone specific gravity
